# Analgesic effects of platelet‐rich fibrin (PRF): A systematic review

**DOI:** 10.1111/prd.70014

**Published:** 2025-10-13

**Authors:** Nathan E. Estrin, Troy B. Tran, Paras Ahmad, Nima Farshidfar, Georgios E. Romanos, Anton Sculean, Richard J. Miron

**Affiliations:** ^1^ The University of Iowa College of Dentistry and Dental Clinics Iowa City Iowa USA; ^2^ Lake Erie College of Osteopathic Medicine School of Dental Medicine Bradenton Florida USA; ^3^ Department of Research, Advanced PRF Education Jupiter Florida USA; ^4^ Department of Periodontology University of Bern Bern Switzerland; ^5^ Department of Periodontics and Endodontics, Laboratory for Periodontal‐, Implant‐, Phototherapy (La‐PIP), School of Dental Medicine Stony Brook University Stony Brook New York USA

**Keywords:** oral health, pain, patient‐reported outcome measures, platelet‐rich fibrin, PRF, quality of life

## Abstract

**Background:**

Platelet‐rich fibrin (PRF), a second‐generation autologous platelet concentrate, has gained significant interest for its anti‐inflammatory and regenerative characteristics. While its role in tissue healing is well‐recognized, the analgesic potential of PRF remains under‐investigated.

**Aim:**

The primary objective of this systematic review was to critically evaluate any pain‐reported outcome of PRF across all medical and dental procedures in human studies. The secondary objective was to also evaluate outcomes regarding swelling reduction with PRF and other patient‐reported outcomes such as quality of life and analgesic consumption in all included studies.

**Methods:**

A systematic search of PubMed, Scopus, Web of Science, and Google Scholar databases was performed for comparative clinical studies assessing PRF's influence on postoperative pain. Eligible studies included human clinical trials comparing PRF with non‐PRF controls, with pain‐reported outcomes as the primary outcome. Data on swelling and other patient‐reported outcomes, including analgesic use and quality of life, was also evaluated as a secondary objective; however, studies that evaluated these outcomes alone were excluded. A total of 200 comparative clinical studies were included, covering a diverse range of procedures including third molar extractions, palatal wound healing, mucogingival procedures, periodontal/bone procedures, maxillary sinus lifts, endodontic procedures, orthodontic procedures, oral lesions, alveolar osteitis, oroantral communications, medically induced osteonecrosis of the jaw, temporomandibular joint disorders, orthopedic procedures, facial surgery and aesthetics, and other fields of medicine. However, heterogeneity in PRF preparation methods and outcome measures precluded a meta‐analysis.

**Results:**

Almost all studies reported reduced pain levels in the PRF group compared with non‐PRF controls, with additional benefits observed in terms of swelling reduction, decreased analgesic use, and improved patient‐reported outcomes. Importantly, it was observed that procedures that tend to generate the most patient‐reported pain, such as 3rd molar extractions and autogenous soft tissue grafting from the hard palate, generally reported much lower pain scores following PRF use (72%–85% of studies) and significantly reduced postoperative analgesic use (87.5% of studies).

**Conclusions:**

The autologous nature of PRF, along with the sustained release of bioactive factors, likely plays a vital role in modulating inflammation and promoting tissue healing, hence enhancing patient comfort and recovery. As PRF continues to gain traction in clinical practice, integrating well‐designed comparative studies with standardized outcome measures will be necessary to completely understand its therapeutic potential and inform evidence‐based guidelines regarding its application.

## 1 INTRODUCTION

Postoperative pain management remains a critical component of patient care across a wide spectrum of medical and dental surgical procedures. When inadequately addressed, acute postoperative pain not only contributes to patient distress but can also impede tissue healing, increase the risk of complications, prolong recovery time, and reduce patient compliance.^1^, ^2^ While the severity and duration of postoperative pain may vary considerably between individuals, several patient‐ and procedure‐associated factors, such as age, sex, type and duration of surgery, psychological state, and baseline anxiety have been shown to affect pain perception and response significantly.^3^, ^4^, ^5^

Pain following surgical trauma usually evolves through two overlapping phases: an immediate, sharp nociceptive response at the site of tissue injury, followed by a sustained, diffuse pain modulated by inflammatory signaling around the affected area.^6^ It is well‐established that a cascade of pro‐inflammatory cytokines and mediators, including interleukins (IL), bradykinin, and prostaglandins, released at the surgical site activate peripheral nociceptors, propagating the pain response.^6^, ^7^

While pharmacologic analgesics, such as nonsteroidal anti‐inflammatory drugs (NSAIDs) and opioids, remain the mainstay of postoperative pain control, their use is frequently restricted by systemic adverse effects, delayed wound healing, and increasing concerns over dependency and misuse.^8^, ^9^, ^10^, ^11^ Consequently, there is a growing interest in biological adjuncts that can offer both regenerative and analgesic advantages, potentially alleviating pharmacological burden while improving favorable healing outcomes.^8^, ^9^, ^10^, ^11^

Autologous platelet concentrates (APCs) have gained widespread application in regenerative medicine and dentistry due to their ability to deliver high concentrations of platelets, leukocytes, and growth factors at the site of injury.^12^, ^13^, ^14^ While first‐generation platelet‐rich plasma (PRP) has exhibited therapeutic success, second‐generation platelet‐rich fibrin (PRF) has emerged as a promising alternative due to its simplified preparation and absence of anticoagulants, leading to a more sustained long‐term release of bioactive factors.^15^, ^16^, ^17^

In vitro studies have demonstrated that PRF can mediate the immune response by inducing a phenotypic shift of macrophages from a pro‐inflammatory M1 to an anti‐inflammatory M2 profile,^18^ hence exerting not only regenerative effects, but also potential nociceptive actions. These outcomes have been supported by clinical studies of decreased postoperative pain and improved tissue healing following PRF application.^11^, ^19^

Despite growing interest in the clinical application of PRF, no prior systematic review has comprehensively assessed the analgesic effects of PRF across diverse regenerative and surgical contexts. Therefore, the current review aimed to systematically analyze and synthesize existing evidence on the role of PRF in postoperative pain intensity reduction, analgesic use, swelling, and quality of life, comprising both medical and dental procedures.

## 2 METHODOLOGY

The study protocol was registered with the International Prospective Register of Systematic Reviews (PROSPERO) (Registration Number: CRD420251139773). A comprehensive electronic search was conducted on June 4, 2025, utilizing three major biomedical databases: PubMed (via MEDLINE), Elsevier's Scopus, and Clarivate Analytics' Web of Science (All Databases). The search strategy targeted titles and abstracts of published studies utilizing the following Medical Subject Headings (MeSH) terms and keywords: (“Platelet‐Rich Fibrin” OR “PRF” OR “Platelet‐Rich Fibrin” OR “LPRF” OR “L‐PRF” OR “Advanced PRF” OR “APRF” OR “A‐PRF” OR “Horizontal‐PRF” OR “H‐PRF” OR “HPRF” OR “ALB‐PRF” OR “Albumin PRF”) AND (“Pain” OR “Patient‐reported outcomes” OR “quality of life” OR “Visual analog score” OR “VAS” OR “OHQoL” OR “OHRQoL”).

Moreover, gray literature was screened using Google Scholar to identify potentially relevant unpublished or nonindexed studies. A manual hand search of the bibliography from the eligible studies was also conducted to identify any additional studies that met the inclusion criteria.

Eligibility criteria were established using the Participants, Intervention, Comparison, and Outcome (PICO) framework: Population (P): Human patients undergoing medical or dental procedures in which PRF was applied. Intervention (I): Use of PRF, including all forms (L‐PRF, A‐PRF, i‐PRF, Alb‐PRF). Comparator (C): Conventional healing without PRF, or alternative biomaterials and/or adjunctive therapies. Outcomes (O): Primary outcome: Postoperative pain. Secondary outcomes: Other postoperative patient‐reported outcomes, such as analgesic consumption, postoperative swelling/edema, and quality of life (QoL). The present review included cross‐sectional, case‐control, cohort (i.e., prospective/retrospective), and randomized controlled trials (RCTs) utilizing different forms of PRF. To be eligible, studies were required to evaluate postoperative pain. As a secondary objective, data were collected for swelling and other closely related patient‐reported outcomes (i.e., quality of life [QoL], analgesic consumption, and patient satisfaction). Studies that evaluated only a secondary objective and did not report postoperative pain were excluded. Studies were excluded if they utilized other platelet‐derived preparations with anticoagulants, including PRP, plasma rich in growth factors (PRGF), or concentrated growth factor (CGF), without a comparator group involving PRF. Furthermore, studies were excluded if: (1) PRF was used in both the control and experimental groups; or (2) no comparator group was included. Other exclusions included review articles, conference abstracts, protocol papers, technical notes, letters to the editor, book chapters, and studies not published in English owing to language limitations of the research team.

Two investigators (N.E.E. and P.A.) assessed the methodological quality of the included studies. For the RCTs, the risk of bias (RoB) assessment was conducted using the revised Cochrane Risk of Bias tool for randomized trials.^20^ Each of the included RCTs was assessed in five areas, including (1) RoB arising from the randomization procedure; (2) RoB owing to deviations from the intended treatments; (3) missing outcome data; (4) RoB in the measurement of the outcome; and (5) RoB in the selection of the reported outcomes. A RoB judgment (among “low,” “high,” or “some concerns”) was assigned to each domain (based on the descriptions given for each field) or to the entire study.^20^ For the included non‐RCTs, a methodological quality evaluation was conducted as per the RoB in nonrandomized studies of intervention.^21^ Seven main domains for RoB were evaluated: (1) bias due to confounding; (2) bias in the selection of participants into the study; (3) bias in the classification of interventions; (4) bias due to deviations from intended interventions; (5) bias due to missing data; (6) bias in the measurement of outcomes; and (7) bias in the selection of the reported results. A RoB judgment (among “low,” “moderate,” “serious”) was assigned to each domain (based on the descriptions given for each field) or to the entire study.^21^

## 3 RESULTS

**FIGURE 1:**
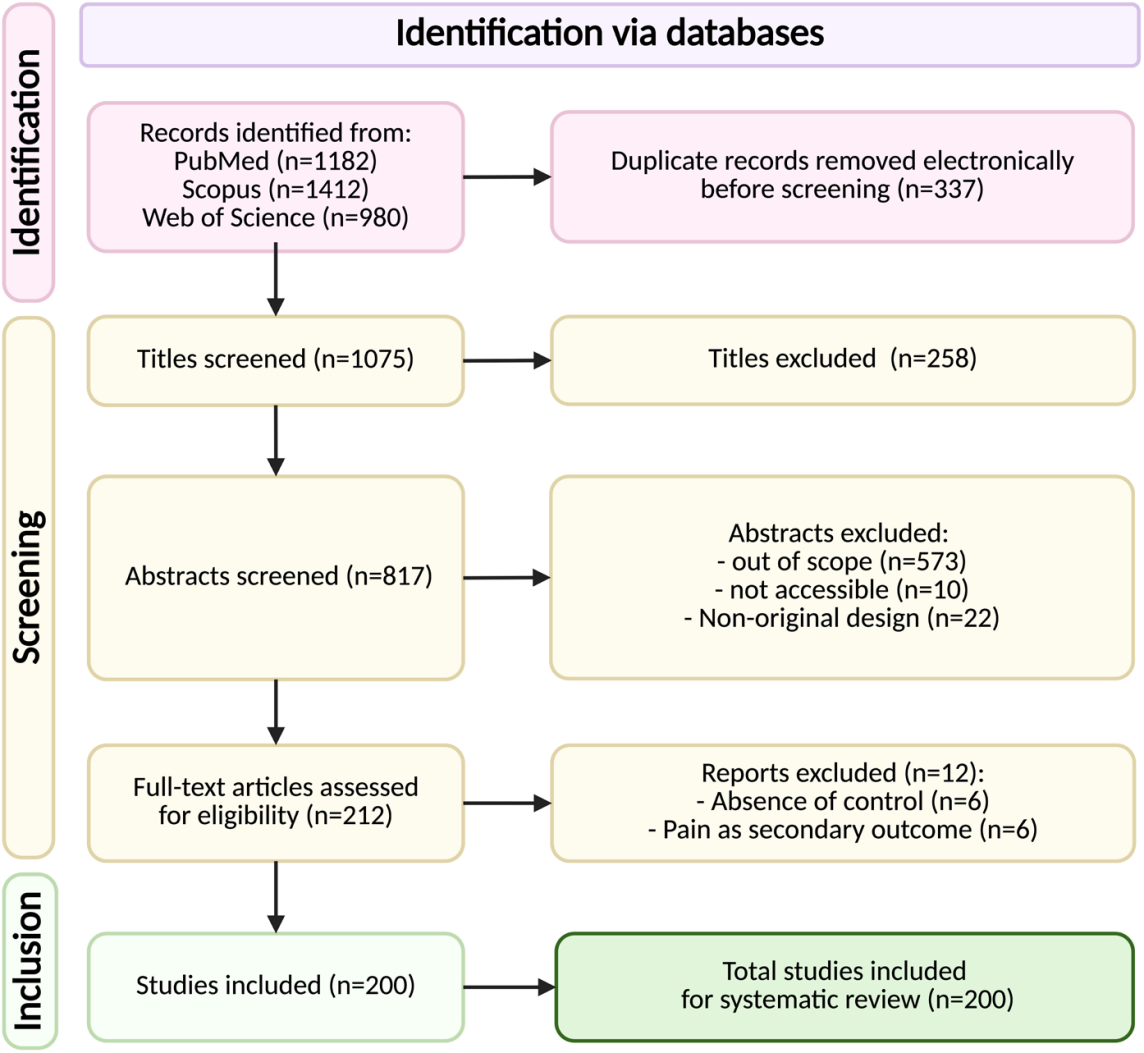
Flow chart for study selection.

A total of 200 articles were included (Figure 1). Sixteen major clinical categories were identified based on the anatomical site and type of surgical intervention in which PRF was used for pain management. These categories included: (1) third molar extractions, (2) dental extractions, (3) palatal wound healing, (4) mucogingival procedures, (5) periodontal/bone procedures, (6) maxillary sinus lifts, (7) endodontic treatments, (8) orthodontic interventions, (9) management of oral lesions, (10) alveolar osteitis, (11) oroantral communications, (12) medication‐related osteonecrosis of the jaw (MRONJ), (13) temporomandibular joint (TMJ) disorders, (14) orthopedic applications, (15) facial surgical and aesthetic procedures, and (16) miscellaneous medical fields.

Of the total included studies, 131 studies (65.5%) reported a statistically significant reduction in postoperative pain in the PRF‐treated groups compared with the control or non‐PRF groups. Contrarily, 62 studies (31.0%) found no significant difference in pain outcomes between PRF and the control, and seven studies (3.5%) reported higher pain levels in the PRF group. Regarding adjunctive postoperative protocols, 74 studies (37.0%) reported prescribing chlorhexidine (CHX), and 117 studies (58.5%) utilized analgesic medications postoperatively.

### 3.1 Third molar extractions

A total of 54 studies were included that assessed the use of PRF in third molar extraction procedures (Table 1). Among these, 46 were RCTs, and eight were prospective non‐RCTs.^22^, ^23^, ^24^, ^25^, ^26^, ^27^, ^28^, ^29^ Thirty‐three studies used a split‐mouth design,^22^, ^23^, ^25^, ^26^, ^27^, ^30^, ^31^, ^32^, ^33^, ^34^, ^35^, ^36^, ^37^, ^38^, ^39^, ^40^, ^41^, ^42^, ^43^, ^44^, ^45^, ^46^, ^47^, ^48^, ^49^, ^50^, ^51^, ^52^, ^53^, ^54^, ^55^, ^56^, ^57^, ^58^ while 21 studies utilized a parallel‐arm design.^24^, ^28^, ^29^, ^59^, ^60^, ^61^, ^62^, ^63^, ^64^, ^65^, ^66^, ^67^, ^68^, ^69^, ^70^, ^71^, ^72^, ^73^, ^74^, ^75^ Among the 54 studies, 39 studies (72.2%) reported significantly reduced postoperative pain in the PRF‐treated group when compared with control or non‐PRF groups.^22^, ^23^, ^24^, ^25^, ^26^, ^27^, ^28^, ^30^, ^34^, ^35^, ^36^, ^37^, ^39^, ^41^, ^42^, ^43^, ^44^, ^45^, ^47^, ^48^, ^50^, ^51^, ^52^, ^54^, ^56^, ^58^, ^59^, ^60^, ^61^, ^64^, ^65^, ^66^, ^67^, ^68^, ^69^, ^72^, ^73^, ^74^, ^75^ Fifteen studies (27.8%) reported no statistically significant difference in pain outcomes between the PRF and control groups.^29^, ^31^, ^32^, ^33^, ^38^, ^40^, ^46^, ^49^, ^53^, ^55^, ^57^, ^62^, ^63^, ^70^, ^71^

**TABLE 1:** Summary of studies evaluating pain reduction with PRF for third molar extractions.

Study and design	Sample size; M/F; mean age (years)	Postoperative medications	Control; test groups	PRF preparation (speed and time; manufacturer)	Parameters assessed	Pain scores	Main findings
Singh et al. (2012)^22^; Split‐mouth prospective	20, 10/10, 32	NR	Surgery (*n* = 10); Surgery + PRF (*n* = 10)	3000 rpm; 10 min; NR	Pain (VAS; 1, 2, 7 d)	*Surgery* = 1 d: 3.8; 3 d: 1.8; 7 d: 0; *Surgery + PRF* = 1 d: 3.6; 3 d: 1.6; 7 d: 0	PRF reduced pain on 1 and 2 d. Both groups had no pain by 7 d
Kumar et al. (2015)^59^; Parallel RCT	31, NR, 26.1	AMOX (500 mg; t.i.d.; 3 d); MTD (400 mg; t.i.d.); Aceclofenac + PCM (b.i.d.); CHX (0.12%; t.i.d.)	Primary closure (*n* = 15); Primary closure + PRF (*n* = 16)	3000 rpm; 10 min; NR	Pain (VAS); Swelling (1 d)	*Primary closure* = 40% mild pain; 40% slight pain; 20% severe pain; *Primary closure + PRF* = 87.5% mild pain; 12.5% slight pain; 0% severe pain	PRF group had less pain and swelling
Uyanik et al. (2015)^23^; Split‐mouth prospective	20, 10/10, 22.5	AMOX (500 mg; t.i.d.; 5 d); ACT (500 mg; PRN)	Surgery (*n* = 20); Surgery + PRF (*n* = 10); Piezosurgery + PRF (*n* = 10)	3000 rpm; 10 min; Elektro‐mag M415P, Turkey	Pain (VAS); analgesics use; Cheek swelling (1, 2, 3, 7 d)	VAS scores (sum of all days) = *Surgery* = 74.6 ± 35.21; *Surgery + PRF* = 25.0 ± 18.99; *Piezosurgery + PRF*:24.45 ± 14.95	PRF reduced pain and analgesic use
Bilginaylar and Uyanik (2016)^24^; Parallel prospective	59, 33/47, 21.9	AMOX (500 mg; t.i.d.; 5 d); PCT (500 mg; PRN); Povidone‐Iodine (7.5% mouthwash; t.i.d.; 7 d)	Surgery (*n* = 20); Surgery + PRF (*n* = 20); Piezosurgery (*n* = 20); Piezosurgery + PRF (*n* = 20)	3000 rpm (400 × *g*); 10 min; Elektro‐mag M415P, Turkey	Pain (VAS); analgesics use; Cheek swelling (1, 2, 3, 7 d)	VAS scores (sum of all days) = *Surgery* = 72.30 ± 38.94; *Surgery + PRF* = 30.28 ± 22.75; *Piezosurgery + PRF* = 32.95 ± 27.30	Lower pain and analgesic use in PRF groups
Kumar et al. (2016)^30^; Split‐mouth RCT	34, NR, NR	NR	Surgery (*n* = 34); Surgery + PRF (*n* = 34)	NR	Pain (NRS; 1, 3 d and 1, 4 w)	*Surgery* = 1 d: 6.0; 3 d: 4.0; 1 w: 1.0, 4 w: 0.0; *Surgery + PRF*: 1 d: 3.0, 3 d: 1.0, 1 w: 0.0, 4 w: 0.0	Significant early pain reduction with PRF
Ozgul et al. (2016)^31^; Split‐mouth RCT	56, 23/33, NR	AMOX (1 g; b.i.d.; 7 d); PCT (500 mg; t.i.d.; 7 d); CHX (t.i.d.; 7 d)	Surgery (*n* = 56); Surgery + PRF (*n* = 56)	3000 rpm; 10 min; NR	Pain (VAS); facial swelling (1, 3, 7 d)	*Surgery* = 1 d: 42.84 ± 29.77; 3 d: 26.48 ± 30.36; 7 d: 9.41 ± 16.57; *Surgery + PRF* = 1 d: 47.16 ± 30.59, 3 d: 25.50 ± 29.95, 7 d: 10.21 ± 19.75	PRF reduced horizontal swelling
Al‐Hamad et al. (2017)^60^; Parallel RCT	47, 13/34, 25.24 ± 7.04	AMOX (500 mg; QID; 5 d); IBU (400 mg; PRN); CHX (b.i.d.; 7 d)	Surgery (*n* = 25); Surgery + PRF (*n* = 25)	3000 rpm; 10 min; 80‐1 Electric Centrifuge, China	Pain (VAS); analgesics use (2–7 d)	*Surgery* = 2 d: 4.24 ± 2.86, 3 d: 2.88 ± 2.36, 4 d: 2.16 ± 2.37, 5 d: 1.28 ± 1.54, 6 d: 0.72 ± 1.40, 7 d: 0.52 ± 1.41; *Surgery + PRF* = 2 d: 3.08 ± 2.75, 3 d: 1.92 ± 2.27, 4 d: 1.20 ± 1.73, 5 d: 0.80 ± 1.55, 6 d: 0.48 ± 1.50, 7 d: 0.00 ± 0.0	Less pain on 5, 6, 7 d and less analgesics on 2, 3, 6, 7 d
Asutay et al. (2017)^32^; Split‐mouth RCT	30, 6/24, 20.32	AMOX/Clav (1 g; b.i.d.; 7 d); PCT (500 mg; b.i.d.; 7 d); CHX (0.12%; t.i.d.; 7 d)	Surgery (*n* = 30); Surgery + PRF (*n* = 30)	2700 rpm; 12 min; NR	Pain (VAS; 6, 12 h, 1–7 d); facial swelling (2, 7 d)	*Surgery* = 6 h: 43.47 ± 32.16, 12 h: 31.00 + 28.83, 1 d: 22.20 + 21.70, 2 d: 18.67 + 22.39, 3 d: 17.73 + 24.90, 4 d: 15.80 + 22.85, 5 d: 13.40 + 22.96, 6 d: 8.27 + 15.59, 7 d: 4.87 + 11.42; *Surgery + PRF* = 6 h: 49.65 ± 31.45, 12 h: 31.24 ± 30.58, 1 d: 27.35 ± 31.70, 2 d: 18.59 ± 19.48, 3 d: 22.00 ± 23.77, 4 d: 14.76 ± 19.00, 5 d: 11.47 ± 16.62, 6 d: 10.47 ± 18.21, 7 d: 8.18 ± 15.52	No significant pain/swelling difference with PRF
Gulsen and Senturk (2017)^33^; Split‐mouth RCT	30, 9/21, 10.03	ACT (500 mg; PRN); AMOX/Clav (1 g; b.i.d.; 5 d); CHX (0.2% t.i.d.; 7 d)	Surgery (*n* = 30); Surgery + PRF (*n* = 30)	3000 rpm; 10 min; NUVE NF 200, Turkey	Pain (VAS, VRS; 6, 12 h, 1, 2, 3, 7 d); facial swelling (2, 7 d)	*Surgery* = 6 h: 40.0 ± 26.3; 12 h: 30.0 ± 28.9; 1 d: 20.9 ± 26.1; 2 d: 13.8 ± 18.4; 3 d: 8.0 ± 12.3; 7 d: 0.8 ± 2.7; *Surgery + PRF* = 6 h: 42.7 ± 27.5; 12 h: 36.1 ± 28.5; 1 d: 25.0 ± 26.3; 2 d: 15.8 ± 20.9; 3 d: 7.9 ± 12.1; 7 d: 1.0 ± 3.0	PRF reduced pain and improved patient satisfaction
Jeyaraj & Chakranarayan (2018)^61^; Parallel RCT	60, 10/20, NR	AMOX (500 mg; t.i.d.; 5 d); IBU (400 mg; t.i.d.; 5 d); PCT (325 mg; t.i.d.; 5 d)	Surgery (*n* = 30); Surgery + PRF (*n* = 30)	2700 rpm; 12 min; NR	Pain (VAS); cheek swelling (3 d)	VAS (average day 3) = *Surgery* = 18.40; *Surgery + PRF* = 14.33	PRF reduced postoperative morbidity compared with control group
Afat et al. (2018)^62^; Parallel RCT	60, 22/38, Control: 22.7 ± 2.94; Test 1: 22.3 ± 1.78; Test 2: 22.15 ± 2.62	NR	Surgery (*n* = 20); Surgery + L‐PRF (*n* = 20); Surgery + L‐PRF + HA (*n* = 20)	3000 rpm; 10 min; NR	Pain (VAS; 6 h, 1–7 d); analgesic use (1–7 d); swelling (2, 7 d)	*Surgery* = 6 h: 3.25 ± 1.77; 1 d: 2.34 ± 1.09; 2 d: 1.5 ± 1.19; 3 d: 1.5 ± 1.73; 4 d: 1.3 ± 1.45; 5 d: 0.85 ± 0.93; 6 d: 0.65 ± 0.93; 7 d: 0.4 ± 0.6; *Surgery + L‐PRF* = 6 h: 3.4 ± 2.23; 1 d: 2.9 ± 1.97; 2 d: 1.85 ± 1.35; 3 d: 1.35 ± 1.39; 4 d: 1.0 ± 1.21; 5 d: 0.6 ± 0.94; 6 d: 0.3 ± 0.66; 7 d: 0.15 ± 0.37; *Surgery + L‐PRF + HA* = 6 h: 4.1 ± 2.45; 1 d: 3.35 ± 2.45; 2 d: 2.45 ± 2.01; 3 d: 1.6 ± 1.6; 4 d: 1.05 ± 1.05; 5 d: 0.6 ± 0.82; 6 d: 0.3 ± 0.47; 7 d: 0.2 ± 0.41	No significant pain difference. L‐PRF + HA had less analgesic use
Dar et al. (2018)^25^; Split‐mouth prospective	30, 13/17, 23.6 ± 4.385	AMOX/Clav (625 mg; b.i.d.; 5 d); Acefenac (100 mg; b.i.d.; 5 d)	Surgery (*n* = 30); Surgery + L‐PRF (*n* = 30)	3000 rpm; 12 min; NR	Pain (VAS); swelling (1, 3, 7, 14 d)	*Surgery* = 1 d: 2.83 ± 1.895; 3 d: 2.20 ± 1.864; 7 d: 1.03 ± 1.542; 14 d: 0.10 ± 0.403; *Surgery + PRF* = 1 d: 0.83 ± 1.020; 3 d: 0.50 ± 0.938; 7 d: 0.23 ± 0.679; 14 d: 0.00 ± 0.000	PRF group had lower pain throughout; less swelling at 3 and 7 d
Daugela et al. (2018)^34^; Split‐mouth RCT	34, 14/20, 23.35 ± 1.73	Clindamycin (600 mg; 1 h pre‐ and 6 h post); Lornoxicam (8 mg; PRN); CHX (0.12%; t.i.d.; 14 d)	Surgery (*n* = 34); Surgery + L‐PRF (*n* = 34)	2800 rpm; 12 min; EBA 20, Andreas Hettich	Pain (VAS; 1–7 d); swelling (1, 3, 7 d)	*Surgery* = 1 d: 4.20 ± 1.35; 2 d: 3.53 ± 1.28; 3 d: 3.13 ± 1.28; 4 d: 2.97 ± 1.13; 5 d: 2.57 ± 1.10; 6 d: 1.97 ± 0.85; 7 d: 1.53 ± 0.82; *Surgery + L‐PRF* = 1 d: 2.87 ± 0.97; 2 d: 2.67 ± 1.03; 3 d: 1.67 ± 0.88; 4 d: 1.37 ± 0.72; 5 d: 1.13 ± 0.82; 6 d: 0.67 ± 0.76; 7 d: 0.07 ± 0.25	L‐PRF significantly reduced pain during 1 w and swelling on 1 and 3 d compared with the control group
Revathy et al. (2018)^35^; Split‐mouth RCT	25, 15/10, NR	AMOX (500 mg t.i.d.; 3 d); MTD (400 mg; t.i.d.; 3 d); Aceclofenac + PCT (100/325 mg; b.i.d.; 3 d)	Surgery (*n* = 25); Surgery + PRF (*n* = 25)	3000 rpm; 10 min; NR	Pain and swelling (as a complication)	*Surgery* = mild pain and swelling (*n* = 15) *Surgery + PRF* = diffuse pain and swelling (*n* = 5)	Fewer complications in PRF groups (5 vs. 15)
Unsal and Erbasar (2018)^36^; Split‐mouth RCT	50, 17/33, 23.96	PCT (500 mg; t.i.d.; 7 d); CHX (0.2%; t.i.d.; 7 d)	Surgery (*n* = 50); Surgery + PRF (*n* = 50)	3000 rpm; 10 min; NF 200 centrifuge; Nuve, Turkey	Pain (VRS; 6, 12, 24, 48, 72 h and 7 d)	*Surgery* = 6 h: 2.32; 12 h: 2.10; 24 h: 1.94; 48 h: 1.56; 72 h: 1.40; 7 d: 1.22; *Surgery + PRF* = 6 h: 2.12; 12 h: 1.88; 24 h: 1.38; 48 h: 1.08; 72 h: 0.88; 7 d: 0.74	PRF reduced pain at all timepoints
Kapse et al. (2019)^37^; Split‐mouth RCT	60, 13/17, 25.47 ± 0.90	NR	Surgery (*n* = 60); Surgery + L‐PRF (*n* = 60)	2700 rpm; 12 min; R‐4C DX, REMI, India	Pain (VAS); swelling (1, 3, 7, 14 d)	*Surgery* = 1 d: 30.17 ± 2.28; 3 d: 34.60 ± 2.32; 7 d: 18.90 ± 1.35; 14 d: 9.47 ± 1.12; *Surgery + L‐PRF* = 1 d: 20.77 ± 1.74; 3 d: 11.17 ± 1.70; 7 d: 3.30 ± 0.80; 14 d: 0.73 ± 0.22	PRF group showed lower pain and swelling
Ritto et al. (2019)^38^; Split‐mouth RCT	17, 10/7, 21.8	DEX (8 mg; 1 h pre‐op); IBU (400 mg; every 6 h; 5 d); ACT (750 mg; every 6 h; 5 d)	Surgery (*n* = 60); Surgery + L‐PRF (*n* = 60)	2700 rpm (400 × *g*); 12 min; NR	Pain (VAS; 1, 3, 7 d)	*Surgery* = 1 d: 3.98 ± 2.97; 3 d: 3.11 ± 2.61; 7 d: 2.11 ± 3.04; *Surgery + L‐PRF* = 1 d: 3.00 ± 2.81; 3 d: 2.85 ± 2.17; 7 d: 1.53 ± 2.50	No significance difference in pain
Zahid and Nadershah (2019)^39^; Split‐mouth RCT	10, 0/10, 24	AMOX (500 mg; t.i.d.; 3 d); IBU (600 mg; t.i.d.; 2 d); CHX (0.12%; t.i.d.; 7 d)	Surgery (*n* = 10); Surgery + A‐PRF (*n* = 10)	1300 rpm; 13 min; Duo, Process for PRF Company	Pain (VAS); swelling (7, 15, 90 d)	*Surgery* = 7 d: 5 (4.25–6.75) *Surgery + A‐PRF* = 7 d: 5 (2.0–4.75)	A‐PRF significantly reduced pain and swelling
Malhotra et al. (2020)^40^; Split‐mouth RCT	50, NR, NR	NR	Surgery (*n* = 50); Surgery + PRF (*n* = 50)	3000 rpm (400 × *g*); 10 min; Remi Model C‐854/6	Pain (VAS); swelling (1 w, 1, 2 m)	*Surgery* = 1 w: 3.27; 1 m: 0.47; 2 m: 0.00 *Surgery + A‐PRF* = 1 w: 2.87; 1 m: 0.40; 2 m: 0.06	No statistically significant in outcomes
Miyamoto et al. (2020)^41^; Split‐mouth RCT	32, 14/18, 29.1	AMOX (750 mg; OD; 4 d); Loxoprofen sodium hydrate (60 mg; PRN)	Surgery (*n* = 32); Surgery + PRF (*n* = 32)	400 × *g*; 10 min; NR	Pain (VAS; 1–7 d)	*Surgery* = 1 d: 43.90 ± 4.58; 3 d: 26.69 ± 3.37; 4 d: 25.37 ± 4.08; *Surgery + PRF* = 1 d: 46.25 ± 4.45; 3 d: 16.78 ± 2.94; 4 d: 13.97 ± 2.65	PRF showed benefit only on 3–4 d
Rupawala et al. (2020)^26^; Split‐mouth Prospective	47, 16/31, 26.83 ± 6.58	AMOX (500 mg; t.i.d.; 5 d); Diclofenac sodium (50 mg) + PCT (500 mg; b.i.d.)	Surgery (*n* = 47); Surgery + sticky bone (AFG + Alloplast) (*n* = 47)	2400–2700 rpm; 2 min; REMI R4C, REMI Laboratory Instruments, India	Pain (NRS); swelling (3, 7, 14 d)	*Surgery* = 3 d: 7.75 ± 0.622; 7 d: 4.83 ± 0.835; 14 d: 0.08 ± 0.289; *Surgery + sticky bone* = 3 d: 7.25 ± 0.622; 7 d: 4.25 ± 0.622; 14 d: 0.08 ± 0.289	Sticky bone group had significantly lower pain and swelling
Sybil et al. (2020)^42^; Split‐mouth RCT	25, 14/11, 32.3	NR	Surgery (*n* = 25); Surgery + PRF (*n* = 25)	3000 rpm; 10 min; NR	Pain (VAS); swelling; tenderness (VAS; 1, 3 d, 1 w, 1 m); sensitivity Cold air spray; 1 w, 1, 3, 6 m	*Surgery* = 1 d: 2.08 ± 1.352; 3 d: 1.80 ± 1.041; 1 w; 4.48 ± 1.584; 1 m: 0; *Surgery + PRF* = 1 d: 0.80 ± 0.764; 3 d: 0.56 ± 0.712; 1 w: 3.24 ± 1.422; 1 m: 0	Significant improvement in pain, sensitivity and edema in PRF group
Torul et al. (2020)^63^; Parallel RCT	75, 23/52, 22.31 ± 4.65	NR	Surgery (*n* = 25); Surgery + A‐PRF (*n* = 25); Surgery + CGF (*n* = 25)	1300 rpm; 14 min; DUO centrifuge system, Process for PRF, France	Pain (VAS; 6 h, 7 d); analgesics use; swelling (2, 7 d)	NR	No significant differences in pain, swelling and analgesic use among groups
Da Silva et al. (2021)^45^; Split‐mouth RCT	20, 6/14, 23	PCT (500 mg; QID); IBU (400 mg; t.i.d.); CHX (0.12%; b.i.d.; 7 d)	Surgery + natural blood clot (*n* = 20); Surgery + L‐PRF (*n* = 20)	2700 rpm (708 × *g*); 12 min; IntraSpin™, Biohorizons®, USA	Pain (VAS); analgesics use (1, 2, 3, 7 d)	NR	L‐PRF significantly reduced pain in the first week
Gupta and Agarwal (2021)^43^; Split‐mouth RCT	20, 8/12, NR	NR	Surgery (*n* = 20); Surgery + A‐PRF (*n* = 20)	1500 rpm; 14 min; NR	Pain (VAS); analgesics use; swelling (1, 3, 7 d)	*Surgery* = 1 d: 6.75 ± 0.69; 3 d: 3.80 ± 1.02; 7 d: 0.45 ± 0.66; *Surgery + A‐PRF* = 1 d: 6.55 ± 0.74; 3 d: 2.60 ± 1.62; 7 d: 0.10 ± 0.3	PRF significant reduced pain, swelling and analgesic use on 3 and 7 d
Nourwali (2021)^64^; Parallel RCT	20, NR, NR	AMOX/Clav (625 mg) or Clindamycin (300 mg; t.i.d.; 5 d); IBU (400 mg; t.i.d.; 5 d); Normal saline mouthwash (t.i.d.; 7 d)	Surgery (*n* = 10); Surgery + PRF (*n* = 10)	3000 rpm; 10 min; NR	Pain (VAS; 1, 2, 6 h, evening and morning post‐surgery); swelling (1, 2, 3, 7 d)	*Surgery (pain)* = 1 h: 50% no, 30% mild–moderate; 20% severe; 2 h: 30% no, 50% mild–moderate, 20% severe; 6 h: 10% no, 70% mild–moderate, 20% severe; Evening after: 10% no, 70% mild–moderate, 20% severe; Morning after: 20% no, 40% mild–moderate, 40% severe; *Surgery + PRF (pain)* = 1 h: 60% no, 40% mild–moderate; 2 h: 40% no, 50% mild–moderate, 10% severe; 6 h: 20% no, 60% mild–moderate, 20% severe; Evening after: 50% no, 60% mild–moderate; Morning after: 30% no, 70% mild–moderate	PRF group had significantly less pain, swelling and better sleep
Nowak et al. (2021)^65^; Parallel RCT	60, 38/22, Control: females: 22.64 ± 2.46 Males: 26.58 ± 3.80 Test: females: 25.91 ± 5.61 Males: 25.42 ± 3.55	AMOX (1 g; b.i.d.) NSAIDs	Surgery (*n* = 30); Surgery + A‐PRF (*n* = 30)	1500 rpm; 4 min; Duo Quattro, Process for PRF, France	Pain, burning, dry mouth, swelling, (Questionnaire) 7 d	*Surgery (pain)* = 7 d: 27 (90% patients) *Surgery + A‐PRF* = 7 d – No patients reported pain	A‐PRF reduced symptoms and CRP after 7 d
Sharma et al. (2021)^44^; Split‐mouth RCT	60, 24/36, NR	NR	Surgery (*n* = 30); Surgery + PRF (*n* = 30)	NR	Pain (VAS; 1 d, 1, 4, 8 w, 4, 6 m)	*Surgery* = 1 d: 1.72 ± 0.73; 1 w: 1.00 ± 0.65; 4 w: 0.35 ± 0.49; 8w: 0 ± 0; 12 w: 0 ± 0; 16 w: 0 ± 0; 20 w: 0 ± 0; 24 w: 0 ± 0; *Surgery + PRF*: 1 d: 1.20 ± 0.70; 1 w: 0.60 ± 0.60; 4 w: 0.15 ± 0.37; 8 w: 0 ± 0; 12 w: 0 ± 0; 16 w: 0 ± 0; 20 w: 0 ± 0; 24 w: 0 ± 0	Lower pain in PRF group at 1 w
Starzynska et al. (2021)^66^; Parallel RCT	100, 35/65, Control: 28.5 ± 5.7 Test: 29.3 ± 7.4	AMOX/Clav (875/125 mg; b.i.d.; 7 d); Ketoprofenum (100 mg; PRN)	Surgery (*n* = 50); Surgery + A‐PRF (*n* = 50)	1500 rpm; 14 min; All Centrifuge, Scilogex, LLC, USA	Pain (VAS); analgesics use; swelling (3, 7, 14 d)	*Surgery*: 3 d: 0–3 (22% patients); 4–6 (50% patients); 7–10 (28% patients); 7 d: 0–3 (96% patients); 4–6 (4% patients); 14 d: 0–3 (100% of patients); *Surgery + A‐PRF*: 3 d: 0–3 (84% patients); 4–6 (16% patients); 7 d: 0–3 (98% patients); 4–6 (2% patients); 14 d: 0–3 (100% patients)	PRF reduced pain, analgesic use and swelling on 3 and 7 d
Trybek et al. (2021)^67^; Parallel RCT	90, 28/62, Control: 26.09 ± 7.04; Test: 26.16 ± 5.85	Ketoprofen (100 mg; b.i.d.; 3 d); Clindamycin (600 mg; 1 dose pre‐operatively); CHX (0.1%; t.i.d.; 3 d)	Surgery (*n* = 45); Surgery + PRF (*n* = 45)	2700 rpm; 12 min; EBA‐200, Germany	Pain (NRS; 6 and 12 h, 1–7 d); swelling (1, 2, 7 d)	*Surgery*: 6 h: 5.38 ± 2.16; 12 h: 5.11 ± 1.87; 1 d: 4.49 ± 1.96; 2 d: 3.64 ± 1.98; 3 d: 2.91 ± 1.99; 4 d: 2.47 ± 1.89; 5 d: 1.98 ± 1.84; 6 d: 1.47 ± 1.46; 7 d: 1.11 ± 1.35; *Surgery + PRF*: 6 h: 4.0 ± 2.08; 12 h: 4.31 ± 1.99; 1 d: 3.29 ± 1.87; 2 d: 2.87 ± 1.6; 3 d: 2.13 ± 1.59; 4 d: 1.82 ± 1.56; 5 d: 1.4 ± 1.45; 6 d: 1.07 ± 1.29; 7 d: 0.58 ± 0.75	PRF lowered pain at 6 h, 1 and 3 d. No difference in swelling.
Osagie et al. (2022)^75^; Parallel RCT	50, 17/33, 26.5 ± 7.8	AMOX (500 mg; t.i.d.; 5 d); MTD (400 mg; t.i.d.; 5 d); Sodium diclofenac (50 mg; b.i.d.; 3 d)	Surgery + PRP (*n* = 25); Surgery + PRF (*n* = 25)	2675 rpm (400 × *g*); NR; NR	Pain (VAS); swelling (1, 3, 7 d)	NR	PRF group had significantly less pain vs. PRP. No difference in swelling.
Riaz et al. (2022)^27^; Split‐mouth Prospective	10, 3/7, 26.5	AMOX (500 mg; t.i.d.; 5 d); Combiflam (t.i.d.; 5 d); MTD (400 mg; 5 d); Ranitidine (150 mg; t.i.d.; 5 d)	Surgery + spontaneous healing (*n* = 10); Surgery + PRF (*n* = 5); Surgery + A‐PRF (*n* = 5)	*Standard PRF*: 3000 rpm; 10 min; REMI C‐852, Remielektrotechnik Ltd., India; *A‐PRF*: 1500 rpm; 14 min; NR	Pain (VAS); swelling (1, 3, 7 d)	*Surgery*: 1 d: 3.0; 3 d: 2.4; 7 d: 1.4; *Surgery + PRF*: 1 d: 2.4; 3 d: 1.4; 7 d: 0.6; *Surgery*: 1 d: 2.6; 3 d: 2.0; 7 d: 1.4; *Surgery + A‐PRF*: 1 d: 2.6; 3 d: 1.4; 7 d: 0.4	A‐PRF had reduced pain and swelling vs. standard PRF
Shruthi et al. (2022)^68^; Parallel RCT	44, NR, Control: 28.73 ± 5.17; Test: 30.09 ± 5.32	NR	Surgery (*n* = 22); Surgery + PRF (*n* = 22)	NR	Pain (VAS); swelling (1, 3, 7 d)	*Surgery*: 1 d: 4.91 ± 1.51; 3 d: 5.09 ± 1.15; 7 d: 1.73 ± 0.63; *Surgery + PRF*: 1 d: 4.86 ± 1.78; 3 d: 4.45 ± 1.65; 7 d: 0.68 ± 0.57	Statistically significant lower pain in PRF group on 7 d
Al‐Saadi and Al‐Quisi (2023)^69^; Parallel RCT	66, 29/37, NR	NR	Surgery (*n* = 22); Surgery + HA (*n* = 22); Surgery + A‐PRF (*n* = 22)	1500 rpm; 14 min; NR	Pain (VAS); swelling (1, 3, 7 d)	NR	A‐PRF > HA in reducing pain on 3 d; both > control
Asif et al. (2023)^70^; Parallel RCT	180, 90/90, 41.35 ± 9.87	AMOX (1 g; b.i.d.; 5 d); Naproxen (550 mg; b.i.d.; 5 d)	Surgery (*n* = 90); Surgery + PRF (*n* = 90)	3500 rpm; 10 min; NR	Pain (VAS; 1, 3 d)	Average VAS score = 3.22 ± 1.09	No pain difference, but PRF reduced alveolar osteitis
Kaylani et al. (2023)^58^; Split‐mouth RCT	25, NR, NR	NR	Surgery + Natural blood clot (*n* = 25); Surgery + L‐PRF (*n* = 25)	2800 rpm; 12 min; NR	Pain (VAS; 1, 3, 7 d)	*Surgery* = 1 d: 5.96 ± 0.35; 3 d: 5.86 ± 0.98; 7 d: 2.86 ± 0.84; *Surgery + L‐PRF* = 1 d: 4.10 ± 2.60; 3 d: 2.93 ± 1.05; 7 d: 0.43 ± 0.31	L‐PRF group had significantly less pain on 1 and 7 d
Pereira et al. (2023)^46^; Split‐mouth RCT	16, NR, NR	AMOX (500 mg; 7 d); Sodium diclofenac (50 mg; t.i.d.; 3 d); CHX (0.12% 14 d)	Surgery + natural blood clot (*n* = 12); Surgery + A‐PRF (*n* = 12)	1300 rpm (200 × *g*); 8 min; SpinPlus Titan, SpinLab, Brazil	Pain (VAS; 3, 7, 14, 30, 90 d); swelling (7, 14 d)	*Surgery*: 3 d: 1.50 (0.00–3.75) *Surgery + A‐PRF*: 3 d: 2.00 (0.00–4.75)	No significant differences in pain or swelling
Rodrigues et al. (2023)^47^; Split‐mouth RCT	22, 7/15, 22.41 ± 2.75	PCT (750 mg; PRN); CHX (0.12%; 7 d)	Surgery (*n* = 22); Surgery + PRF (*n* = 22)	2700 rpm (400 × *g*); 12 min; Daiki DT4000 Centrifuge	Pain (VAS; 30 min, 2, 4, 6, 8, 16, 24, 48, 72 h, 7 d); swelling; (immediate postoperatively, 3, 7 d)	*Surgery* = 30 min: 0.0 ± 0.0; 2 h: 0.0 ± 0.0; 4 h: 0.59 ± 0.59; 6 h: 1.86 ± 1.17; 8 h: 2.14 ± 1.58; 12 h: 1.00 ± 1.38; 16 h: 0.95 ± 1.13; 24 h: 3.50 ± 1.19; 48 h: 3.64 ± 1.36; 72 h: 2.55 ± 1.37; 7 d: 0.0 ± 0.0; *Surgery + L‐PRF* = 30 min: 0.0 ± 0.0; 2 h: 0.0 ± 0.0; 4 h: 0.36 ± 0.49; 6 h: 0.55 ± 0.67; 8 h: 0.77 ± 0.75; 12 h: 0.50 ± 0.80; 16 h: 0.36 ± 0.73; 24 h: 1.14 ± 0.83; 48 h: 1.55 ± 0.96; 72 h: 0.68 ± 0.89; 7 d: 0.0 ± 0.0	PRF reduced pain at 4, 6, 8, 16, 24 and 72 h and significantly reduced swelling
Tanan Karaca et al. (2023)^48^; Split‐mouth RCT	48, 17/38, 24.5 ± 4.5	AMOX/Clav (1 g); ACT (500 mg); CHX (0.12%); Benzydamine hydrochloride (0.15%)	Surgery (*n* = 48); Surgery + PRF (*n* = 48)	2700 rpm; 12 min; Intra‐Lock Int. Inc., USA	Pain (VAS); swelling (2 and 7 d)	*Surgery* = 2 d: 4.83 ± 1.0; 7 d: 0.71 ± 0.71 *Surgery + PRF* = 2 d: 2.88 ± 0.79; 7 d: 0.31 ± 0.59	PRF significantly reduced pain, edema, ESR and CRP on 2 d
Akpinar and Ayranci (2024)^57^; Split‐mouth RCT	35, 9/26, 19.97 ± 2.07	AMOX/Clav (1 g; b.i.d.); Arveles (25 mg); Chlorobene (CHX [0.12%] and Benzydamine hydrochloride [0.15%])	Surgery (*n* = 35); Surgery + i‐PRF (*n* = 35)	700 rpm; 3 min; Intra‐Lock Int. Inc., USA	Pain (VAS); analgesics use (6 h and 1–7 d); swelling (2, 7 d); QoL (PoSSe; 7 d)	*Surgery* = 6 h: 68.28; 1 d: 55.71; 2 d: 43.71; 3 d: 27.71; 4 d: 13.14; 5 d: 3.42; 6 d: 1.71; 7 d: 1.14; *Surgery + i‐PRF* = 6 h: 64.85; 1 d: 56; 2 d: 40.28; 3 d: 25.71; 4 d: 14.28; 5 d: 5.42; 6 d: 0; 7 d: 0	No significant pain difference; less analgesic use and swelling in i‐PRF group
Demirok et al. (2024)^49^; Split‐mouth RCT	34, 8/26, 20.24 ± 2.54	AMOX (1 g; b.i.d.; 5 d); Benzydamine hydrochloride; CHX (t.i.d.; 5 d); Etodolac (400 mg; b.i.d.; 5 d)	Surgery + PBM (*n* = 34); Surgery + L‐PRF (*n* = 34)	2700 rpm; 12 min; NR	Pain (VAS); 2, 4, 7 d	*Surgery + PBM* = 2 d: 3.74 ± 2.40; 4 d: 2.56 ± 2.09; 7 d: 1.0 ± 1.37; *Surgery + L‐PRF* = 2 d: 3.65 ± 2.46; 4 d: 2.82 ± 2.17; 7 d: 1.12 ± 1.45	No significant pain difference
El‐Mahmoudy et al. (2024)^50^; Split‐mouth RCT	10, 4/6, 27	NR	Surgery (*n* = 10); Surgery + PRF (*n* = 10)	3000 rpm; 12 min; LC‐04R electric centrifuge. Wincom CO. China	Pain (NR); swelling (1, 3, 7, 14 d)	*Surgery* = 1 d: 9.30 ± 0.79; 3 d: 7.60 ± 1.10; 7 d: 4.30 ± 1.30; 14 d: 0.30 ± 0.42; *Surgery + PRF* = 1 d: 7.10 ± 0.70; 3 d: 5.90 ± 1.43; 7 d: 2.80 ± 1.23; 14 d: 0.0 ± 0.0	PRF significantly reduced pain and swelling except on 14 d
Iftikhar et al. (2024)^51^; Split‐mouth RCT	64, 34/30, 35.82 ± 5.77	NR	Surgery (*n* = 64); Surgery + PRF (*n* = 64)	NR	Pain (VAS); sensitivity; tenderness; swelling (1, 3 d, 1 w, 1, 3, 6 m)	*Surgery* = 1 d: 3.73 ± 1.28; 3 d: 3.64 ± 1.23; 1 w: 1.51 ± 1.26; 1 m: 0.15 ± 0.36; 3 m: 0.0 ± 0.0; 6 m: 0.0 ± 0.0; *Surgery + PRF* = 1 d: 3.18 ± 1.13; 3 d: 3.18 ± 1.24; 1 w: 0.20 ± 0.89; 1 m: 0.0 ± 0.0; 3 m: 0.0 ± 0.0; 6 m: 0.0 ± 0.0	PRF significantly reduced pain and swelling up to 1 m; tenderness improved at 1 w
Kundu et al. (2024)^28^; Parallel Prospective	26, 20/10, 25.4 ± 2.8	Standard Drug Regimen	Surgery (*n* = 13); Surgery + PRF (*n* = 13)	2700 rpm (400 × *g*); 12 min; NR	Pain (VAS; 1, 2, 4, 6, 12, 24, 48, 72 h); analgesics use (1–3 d); swelling (3, 7, 14 d)	*Surgery* = 1 h: 16.5 ± 12.1; 2 h: 78.8 ± 20; 4 h: 51.2 ± 27.1; 6 h: 47.3 ± 23; 12 h: 50.8 ± 15.4; 24 h: 40 ± 17.8; 48 h: 28.5 ± 15.2; 72 h: 16.9 ± 9.47; *Surgery + PRF* = 1 h: 15.4 ± 9.67; 2 h: 51.2 ± 22.2; 4 h: 40.8 ± 16.6; 6 h: 32.7 ± 18.3; 12 h: 26.2 ± 13.3; 24 h: 31.2 ± 19.6; 48 h: 22.7 ± 22; 72 h: 12.3 ± 10.1	PRF group had significantly reduced pain (2 and 12 h), analgesics and swelling (7 and 14 d)
Moraes et al. (2024)^52^; Split‐mouth RCT	34, 12/16, 22.36 ± 4.14	AMOX (500 mg; t.i.d.; 7 d);IBU (600 mg; t.i.d.: 3 d); Dipyrone (500 mg; QID: 3 d)	Surgery (*n* = 34); Surgery + L‐PRF (*n* = 34)	3000 rpm; 10 min; NR	Pain (VAS; 12 h, and 7 d)	*Surgery*: 12 h = 3.36 ± 2.06; 7 d: 1.32 ± 1.81 *Surgery + L‐PRF* = 12 h: 3.11 ± 1.85; 7 d: 0.64 ± 1.06	Pain reduction in L‐PRF group at 7 d
Praganta et al. (2024)^53^; Split‐mouth RCT	70, 23/46, 22.1	ACT (1 g; QID); IBU (400 mg; t.i.d.); Codeine (30 mg; PRN); CHX (0.2%)	2 Gelatin Dressings (*n* = 70); Gelatin dressing + A‐PRF (*n* = 70)	1300 rpm; 14 min; Duo Quattro, Process for PRF, France	Pain (VAS; 1–7 d); swelling (2, 7 d)	*Surgery* = 1 d: 29.5 (23.5, 35.5); 2 d: 29.5 (23.7, 35.3); 3 d: 28.9 (23.4, 34.4); 4 d: 26.9 (21.4, 32.4); 5 d: 21.2 (16.8, 25.6); 6 d: 16.8 (12.3, 21.3); 7 d: 14.2 (10.0, 18.4); *Surgery + A‐PRF* = 1 d: 30.6 (24.5, 36.7); 2 d: 29.6 (23.9, 35.3); 3 d: 32.5 (26.1, 38.9); 4 d: 31.5 (25.5, 37.5); 5 d: 23.1 (17.7, 28.5); 6 d: 20.3 (16.0, 24.6); 7 d: 12.6 (8.7, 16.5)	No statistically significant differences between test and control groups regarding pain and swelling
Selvam et al. (2024)^29^; Prospective Parallel	30, 19/11, 25.67 ± 2.4	AMOX (500 mg; 5 d); PCT and Aceclofenac (5 d)	Surgery (*n* = 15); Surgery + A‐PRF (*n* = 15)	1500 rpm (230 × *g*); 14 min; NR	Pain (VAS; 3, 7 d)	*Surgery (Pain)* = 3 d: 80% no; 20% moderate; 7 d: 6.7% no; 93.3% mild	No significant pain difference
Stefanescu et al. (2024)^54^; Split‐mouth RCT	25, 12/13, 31 ± 2.73	AMOX/Clav (b.i.d.; 7 d);IBU (400 mg; t.i.d.; 5 d); CHX (0.2%; b.i.d. 14 d)	Surgery (*n* = 25); Surgery + A‐PRF (*n* = 25)	1500 rpm; 8 min; Premiere XC‐2000, CandA Scientific, USA	Pain (VAS); swelling (7, 14 d)	*Surgery + A‐PRF* = 3 d: 93.3% no, 6.7% moderate; 7 d: 26.7% no, 73.3% mild	A‐PRF reduced pain and swelling on 7 d; cytokines also decreased
Uranbey and Ayranci (2024)^71^; Parallel RCT	66, 24/44, 21.76 ± 4.43	AMOX/Clav (1 g; b.i.d.); Dexketoprofen (25 mg); CHX (0.15% Oroheks Plus mouthwash)	Surgery (*n* = 15); Surgery + CGF (*n* = 15); Surgery + i‐PRF (*n* = 15)	700 rpm; 3 min; Intra‐Lock Int.	Pain (VAS; 6 h, 1–7 d); analgesics use; swelling (2, 7 d); QoL (PoSSe; 7 d)	*Surgery* = 6 h: 6.73 ± 1.980; 1 d: 3.68 ± 2.495; 2 d: 2.41 ± 1.894; 3 d: 1.64 ± 1.677; 4 d: 1.09 ± 1.411; 5 d: 1.14 ± 1.424; 6 d: 0.82 ± 1.220; 7 d: 0.68 ± 1.211; *Surgery + i‐PRF* = 6 h: 6.18 ± 2.519; 1 d: 4.23 ± 2.245; 2 d: 2.82 ± 2.442; 3 d: 2.36 ± 2.341; 4 d: 2.00 ± 2.268; 5 d: 1.32 ± 1.524; 6 d: 0.77 ± 1.270; 7 d: 0.45 ± 0.671	CGF reduced analgesic use; both CGF and i‐PRF reduced swelling
Yari et al. (2024)^72^; Parallel RCT	64, 28/36, Control: 22.13 ± 3.05 Test 1: 22.19 ± 3.10 Test 2: 22.94 ± 3.54 Test 3: 22.74 ± 3.41	AMOX (500 mg; t.i.d.; 5 d); ACT (500 mg; PRN); CHX (2%; b.i.d.; 7 d)	Surgery (*n* = 16); Surgery + L‐PRF (*n* = 16); Surgery +A‐PRF (*n* = 16); Surgery + A‐PRF+ (*n* = 16)	*L‐PRF*: 2800 rpm; 12 min; *A‐PRF*: 1500 rpm; 14 min; *A‐PRF+*: 1300 rpm 8 min; TD 4, HereXi Instruments Inc.	Pain (VAS); analgesics use; swelling (1, 2, 3, 7 d)	*Surgery* = 1 d: 48.00 ± 11.86; 2 d: 36.13 ± 7.72; 3 d: 17.38 ± 7.27; 7 d: 5.63 ± 4.10; *Surgery + L‐PRF* = 1 d: 38.13 ± 5.43; 2 d: 30.31 ± 3.04; 3 d: 10.63 ± 4.42; 7 d: 2.81 ± 3.15; *Surgery + A‐PRF*: 1 d: 34.38 ± 5.25; 2 d: 23.69 ± 7.72; 3 d: 7.88 ± 4.29; 7 d: 2.56 ± 2.42; *Surgery + A‐PRF+*: 1 d: 34.69 ± 5.62; 2 d: 25.00 ± 6.58; 3 d: 9.13 ± 4.36; 7 d: 2.69 ± 3.60	All test groups reduced pain, swelling, and analgesic use; A‐PRF and A‐PRF + superior to L‐PRF on the second day
Zwittnig et al. (2024)^55^; Split‐mouth RCT	25, 6/19, NR	Methylprednisolone (40 mg); Dexibuprofen (400 mg; PRN)	Surgery (*n* = 25); Surgery + PRF (*n* = 25)	1200 rpm (177 × *g*); 8 min; NR	Pain (VAS; 1–7, 14 d); analgesics use; swelling (1, 3, 7, 14 d)	*Surgery* = 1 d: 4.68 ± 2.80; 2 d: 5.12 ± 2.88; 3 d: 5.00 ± 2.87; 4 d: 4.12 ± 2.68; 5 d: 3.36 ± 2.41; 6 d: 2.56 ± 1.98; 7 d: 1.96 ± 1.79; 14 d: 0.30 ± 0.56; *Surgery + PRF* = 1 d: 4.20 ± 2.16; 2 d: 4.21 ± 2.59; 3 d: 3.84 ± 2.39; 4 d: 3.04 ± 2.07; 5 d: 2.44 ± 2.18; 6 d: 1.92 ± 1.89; 7 d: 1.24 ± 1.23; 14 d: 0.36 ± 0.66	PRF reduced swelling at 14 d, no pain differences but less analgesic use
Agan et al. (2025)^73^; Parallel RCT	60, NR, NR	AMOX/Clav (875/125 mg; b.i.d.; 5 d); PCT (500 mg; QID; 7 d); Povidone‐iodine (7.5%; t.i.d.; 7 d)	Surgery (*n* = 15); Surgery + PRF (*n* = 15); Surgery + PRF + LLLT (*n* = 15); Surgery+LLLT (*n* = 15)	NR	Pain; analgesics use; swelling (1, 2, 3, 7 d)	*Surgery* = 1 d: 49.4 ± 9.2; 2 d: 38.6 ± 12.2; 3 d: 22.2 ± 10.6; *Surgery + PRF* = 1 d: 41.5 ± 8.8; 2 d: 25.4 ± 5.5; 3 d: 10.3 ± 7.2; *Surgery + PRF + LLLT* = 1 d: 31.5 ± 10.2; 2 d: 16.5 ± 6.9; 3 d: 1.7 ± 2.0	All test groups had lower pain, swelling and analgesic use, suggesting LLLT and PRF effectiveness
Barone et al. (2025)^56^; Split‐mouth RCT	32, 9/22, 23.5 ± 3.3	AMOX (1 g) or Clindamycin (600 mg; 1 dose); PCT (1 g) or metamizole (500 mg; PRN); CHX (0.2%; b.i.d.; 7 d)	Surgery (*n* = 32); Surgery + PRF (*n* = 32)	1300 rpm; 8 min; Process for PRF, France	Pain (VAS); swelling (3, 7 d)	*Surgery* = 3 d 4.21 ± 2.55; 7 d: 1.93 ± 1.64 *Surgery + PRF* = 3 d: 4.57 ± 2.87; 7 d: 1.50 ± 2.28	PRF reduced pain and swelling at 3 d
Bilginaylar et al. (2025)^74^; Parallel RCT	36, NR, NR	PCT (500 mg; PRN); Povidone‐iodine (7.5%; t.i.d.; 7 d)	Surgery (*n* = 20); Surgery + PRF + AUG (*n* = 20); Surgery + PRF + Clindamycin (*n* = 20)	3000 rpm; 10 min; Elektro‐mag M415P, Turkey	Pain (VAS); analgesics use; swelling (1, 2, 3, 7 d)	*Surgery* = 1 d: 36.47 ± 1.94; 2 d: 29.77 ± 2.79; 3 d: 17.66 ± 2.94; *Surgery + PRF + AUG* = 1 d: 20.90 ± 1.97; 2 d: 12.25 ± 1.42; 3 d: 4.49 ± 0.53; *Surgery + PRF + Clindamycin* = 1 d: 18.26 ± 2.43; 2 d: 6.81 ± 0.99; 3 d: 1.59 ± 0.46	PRF groups had significantly reduced pain, analgesic use and swelling (1–3 d)

Abbreviations: ACT, acetaminophen; AFG, autologous fibrin glue; Alb‐PRF, albumin platelet‐rich fibrin; AMOX, amoxicillin; A‐PRF, advanced‐platelet‐rich fibrin; A‐PRF+, advanced‐platelet‐rich fibrin plus; AUG, augmentin; b.i.d., bis in die, twice per day; CGF, concentrated growth factor; CHX, chlorhexidine; Clav, clavulanic acid; CRP, C‐reactive protein; d, day; h, hour; HA, hyaluronic acid; IBU, ibuprofen; i‐PRF, injectable platelet‐rich fibrin; LLLT, low‐level laser therapy; L‐PRF, leukocyte‐and platelet‐rich fibrin; m, month; min, minutes; MTD, metronidzaole; NR, not reported; NRS, numeric rating scale; NSAIDs, nonsteroidal anti‐inflammatory drugs; OD, once daily; OHQoL, oral health‐related quality of life; PBM, photobiomodulation; PCT, paracetamol; PoSSe, postoperative symptom severity scale; PRF, platelet‐rich fibrin; PRN, pro re nata, as needed; PRP, platelet‐rich plasma; QID, quater in die, four times a day; RCT, randomized clinical trial; rpm, revolution per minute; t.i.d., ter in die, three times per day; VAS, visual analog scale; VRS, verbal rating scale; w, week.

Postoperative analgesic consumption was reported in 16 studies,^23^, ^24^, ^28^, ^34^, ^43^, ^45^, ^55^, ^57^, ^60^, ^62^, ^63^, ^66^, ^71^, ^72^, ^73^, ^74^ with 14/16 (87.5%) of these reporting reduced analgesic intake in patients treated with PRF.^23^, ^24^, ^28^, ^43^, ^45^, ^55^, ^57^, ^60^, ^62^, ^66^, ^71^, ^72^, ^73^, ^74^ Forty studies reported on postoperative swelling^23^, ^24^, ^25^, ^26^, ^27^, ^28^, ^31^, ^32^, ^33^, ^34^, ^35^, ^37^, ^39^, ^40^, ^42^, ^43^, ^46^, ^47^, ^48^, ^50^, ^51^, ^53^, ^54^, ^55^, ^56^, ^57^, ^59^, ^61^, ^63^, ^64^, ^65^, ^66^, ^67^, ^68^, ^69^, ^71^, ^72^, ^73^, ^74^, ^75^ of which 29 (72.5%) found reduced swelling in the PRF group.^25^, ^26^, ^27^, ^28^, ^31^, ^34^, ^35^, ^37^, ^39^, ^42^, ^43^, ^47^, ^48^, ^50^, ^51^, ^54^, ^55^, ^56^, ^57^, ^59^, ^61^, ^64^, ^65^, ^66^, ^68^, ^69^, ^72^, ^73^, ^74^ Twenty‐one studies prescribed CHX,^31^, ^32^, ^33^, ^34^, ^36^, ^39^, ^45^, ^46^, ^47^, ^48^, ^49^, ^53^, ^54^, ^56^, ^57^, ^59^, ^60^, ^67^, ^71^, ^72^ four studies recommended povidone‐iodine rinses,^23^, ^24^, ^73^, ^74^ and one study recommended saline.^64^ A total of 40 studies reported prescribing analgesic medications as a postoperative regimen.^23^, ^24^, ^25^, ^26^, ^27^, ^29^, ^31^, ^32^, ^33^, ^34^, ^35^, ^36^, ^38^, ^39^, ^41^, ^45^, ^46^, ^47^, ^48^, ^49^, ^52^, ^53^, ^54^, ^55^, ^56^, ^57^, ^59^, ^60^, ^61^, ^64^, ^65^, ^66^, ^67^, ^70^, ^71^, ^72^, ^73^, ^74^, ^75^

### 3.2 Dental extractions

A total of 24 studies assessed the application of PRF in dental extraction procedures (Table 2). All included studies were RCTs. Of these, 20 were designed as parallel‐arm studies,^76^, ^77^, ^78^, ^79^, ^80^, ^81^, ^82^, ^83^, ^84^, ^85^, ^86^, ^87^, ^88^, ^89^, ^90^, ^91^, ^92^, ^93^, ^94^, ^95^ and four used a split‐mouth design.^96^, ^97^, ^98^, ^99^ Thirteen studies reported a statistically significant reduction in postoperative pain in the PRF group compared with the control group.^76^, ^77^, ^78^, ^79^, ^80^, ^82^, ^83^, ^90^, ^91^, ^92^, ^94^, ^96^, ^97^ Ten studies found no significant difference in pain levels between PRF and non‐PRF groups,^81^, ^85^, ^86^, ^87^, ^88^, ^89^, ^93^, ^95^, ^98^, ^99^ and one study reported higher pain scores in the PRF group compared with a chitosan‐based intervention.^84^

**TABLE 2:** Summary of studies evaluating pain reduction with PRF for dental extractions.

Study; Design	Sample size, M/F, Mean Age (years)	Postoperative medications	Control; Test Groups	PRF preparation (speed and time; Manufacturer)	Parameters assessed	Pain scores	Main findings
Marenzi et al. (2015)^1^; Split‐mouth RCT	26, 9/17, 53 ± 4.0	AMOX/clav (875/125 mg; 2 d pre‐ and 3 d post‐op)	Natural healing (*n* = 54); L‐PRF (*n* = 54)	2700 rpm; 12 min; IntraSpin L‐PRF kit, Intra‐Lock, USA	Pain (VAS; 1–4 d)	*Natural healing* = 4.5 ± 0.7 *L‐PRF* = 3.2 ± 0.3	L‐PRF had lower mean pain scores first 3 days
Temmerman et al. (2016)^2^; Split‐mouth RCT	22, 15/7, 54 ± 11.0	IBU (600 mg; t.i.d.; 2 d); CHX (0.12%; b.i.d.; 7 d)	Natural healing (*n* = 11); L‐PRF (*n* = 11)	2700 rpm; 12 min; IntraSpin, Intra‐Lock, USA	Pain (VAS; Every 4 h until 7 d)	*Natural healing* = 1 d: 1.69 (0.27–4.73); 2 d: 2.51 (0.45–4.28); 3 d: 2.45 (0.09–4.64); 4 d: 1.82 (0–4.64); 5 d: 1.25 (0–4.19); 6 d: 0.36 (0–2.68); 7 d: 0.27 (0–4.20) *L‐PRF* = 1 d: 1.69 (0.27–5.53); 2 d: 2.05 (0.45–4.19); 3 d: 1.65 (0.09–3.12); 4 d: 0.89 (0.18–2.58); 5 d: 0.71 (0–3.21); 6 d: 0.09 (0–2.67); 7 d: 0 (0–2.68)	Lower postoperative pain in L‐PRF, significant on day 3,4 and 5
Asmael et al. (2018)^3^; Split‐mouth RCT	20, 20/0, 44.2	NR	Natural healing (*n* = 20); PRF (*n* = 20)	3000 rpm; 10 min (Xiangtian, China)	Pain (VAS; 1, 2, 3 d)	*Natural healing* = 1.8 *PRF* = 0.65	Lower pain in PRF, but not statistically significant
Girish Kumar et al. (2018)^4^; Parallel RCT	48, NR, 44.4 ± 16	Analgesics and antibiotics (5 d)	Natural healing (*n* = 30); PRF (*n* = 30); PRF + POP (*n* = 30)	3000 rpm; 10 min; NR	Pain (VAS; 1, 7 d)	*Natural healing* = 18.1% of patients experienced pain at 24 h, 9.09% of patients experienced pain on 7 d; *PRF* = No patients experienced pain at both time intervals	No pain in PRF groups; pain in control
De Angelis et al. (2019)^5^; Parallel RCT	45, 19/26, Control: 47.7 + 9.1; Test 1: 51.2 + 13.2; Test 2: 52.4 + 16.6	AMOX (1 g; b.i.d.; 6 d); IBU (600 mg; b.i.d.; 3 d); CHX (0.2%; t.i.d.; 7 d)	Xenograft (*n* = 15); PRF (*n* = 15); Xenograft + PRF (*n* = 15)	2700 rpm; 12 min; IntraSpin System; Intra‐Lock Int., USA	Pain (VAS; 1–7 d)	NR	PRF reduced pain vs. xenograft day 2–5
Sarkar et al. (2019)^6^; Parallel RCT	60, 29/31, 58.77 ± 8.88	Routine antibiotics and NSAIDs	Chitosan Hydrogel (*n* = 30); PRF (*n* = 30)	3000 rpm; 10 min; NR	Pain (VAS; 1, 3, 7 d)	*Chitosan Hydrogel* = 1 d: 3.4; 3 d: 1.67; 7 d: 0.53 *PRF* = 1 d: 3.2; 3 d: 1.4; 7 d: 0.37	PRF had lower pain than chitosan on Days 1, 3 and 7
Ustaoglu et al. (2019)^7^; Parallel RCT	57, 28/29, 35.4 ± 5.6	PCT (500 mg; PRN); CHX (0.12%; b.i.d.; 7 d)	Natural Healing (*n* = 19); L‐PRF (*n* = 19); T‐PRF (*n* = 19)	*L‐PRF*: 2700 rpm; 12 min; *T‐PRF*: 2800 rpm; 12 min; IntraSpin System, Intra‐Lock, USA	Pain (VAS); analgesics use (1–3 d)	*Natural healing* = 1 d: 5.11 ± 1.60; 2 d: 1.01 ± 1.44; 3 d: 0; *L‐PRF* = 1 d: 3.30 ± 2.07; 2 d: 0.48 ± 0.92; 3 d: 0; *T‐PRF* = 1 d: 3.29 ± 1.85; 2 d: 0.47 ± 0.62; 3 d: 0	Lower pain day 1 in PRF groups; no significance later
Mourao et al. (2020)^8^; Parallel RCT	32, 13/19, Control: 38.1 ± 10.5; Test: 36.5 ± 11.4	IBU (400 mg; PRN); CHX (0.12%; b.i.d.; 14 d)	Natural healing (*n* = 16); L‐PRF (*n* = 16)	2700 rpm (708 × *g*); 12 min; IntraSpin™, Biohorizons®, USA	Pain (VAS); analgesics use (1 w)	*Natural healing* = 1 w: 5.12 ± 1.08; *L‐PRF* = 1 w: 4 ± 1.15	PRF group had less pain and fewer analgesics
Palma et al. (2020)^9^; Parallel RCT	33, 19/4, Control: 65.5 ± 10.4; Test: 59.2 ± 8.1	Clindamycin (300 mg; t.i.d.; 10 d); Dipyrone‐oral solution (500 mg; QID; 3 d)	Natural healing (*n* = 12); L‐PRF (*n* = 11)	400 × *g*; 12 min; Daiki DT‐4000 Centrifuge™; Ionlab Equipamentos Laboratoriais e Hospitalares Ltda, Brazil	Pain (VAS; 3, 7 d)	*Natural healing* = 3 d: 0 ± 0; 7 d: 0 ± 0; *L‐PRF* = 3 d: 0.2 ± 0.6; 7 d: 0 ± 0	No significant differences in pain
Brazdeikyte et al. (2021)^10^; Parallel RCT	43, 10/33, 28.6	NSAIDs (1 h post‐op)	Hemostatic sponge + Gentamicin (*n* = 21); PRGF (*n* = 11); PRF (*n* = 11)	2800 rpm; 12 min; A‐PRF 12, Advanced PRF, USA	Pain (VAS; 1, 7 d)	*Hemostatic sponge + Gentami in* = 1 d: 5.14 ± 2.762; *PRGF* = 1 d: 4.45 ± 2.583; *PRF* = 1 d: 4.82 ± 3.459	PRGF < PRF < control group in pain
Makki et al. (2021)^11^; Parallel RCT	60, 29/31, NR	NR	Natural healing (*n* = 20); L‐PRF (*n* = 20); A‐ PRF (*n* = 20)	*L‐PRF*: 2700 rpm; 12 min; *A‐PRF*: 1500 rpm; 14 min Medifuge™ Small Benchtop Centrifuge	Pain (VAS; 1, 2 d); analgesics use (6, 12, 18, 24 h)	*Natural healing* = 1 d: 0% had no pain, 64.3% (nonsurgical extractions) had moderate, 14.3% (nonsurgical extractions) had severe; 2 d: 0% had no pain, 64.3% (nonsurgical extractions) had moderate, 7.1% (nonsurgical extractions) had severe; *L‐PRF* = 1 d: 0% had no pain, 66.7% (nonsurgical extractions) had moderate, 16.7% (nonsurgical extractions) had severe; 2 d: 8.3% (nonsurgical extractions) had no pain, 25% (nonsurgical extractions) had moderate, 0% had severe; *A‐ PRF* = 1 d: 27.3% (nonsurgical extractions) had no pain; 27.3% (nonsurgical extractions) had moderate, 0% (nonsurgical extractions) had severe; 2 d: 63.6% (nonsurgical extractions) had no pain, 0% (nonsurgical extractions) had moderate, and 0% had severe.	A‐PRF had lowest pain day 1; control used more medications
Rajendra et al. (2021)^12^; Parallel RCT	300, NR, NR	AMOX (2 g; 1 dose pre‐op)	Chitosan (Axiostat) (*n* = 150); PRF (*n* = 150)	3000 rpm; 10 min; NR	Pain (VAS; 7 d)	*Chitosan (Axiostat)* = 7 d: 1.05 ± 0.87; *PRF* = 7 d: 1.86 ± 0.06	Chitosan had lower pain than PRF
Santhanakrishnan et al. (2021)^13^; Parallel RCT	50, 23/27, Control: 30.8 ± 6.5; Test: 29.8 ± 9.7	AMOX (500 mg; t.i.d.; 5 d); IBU (400 mg; t.i.d.; 5 d); CHX (0.2%; 14 d)	Immediate implant placement + Xeno raft (*n* = 25); Delayed Implant placement + Xenograft + A‐PRF (*n* = 25)	1300 rpm; 14 min; NR	Pain (VAS; Every other day for 1 w)	*Immediate implant placement + Xeno raft* = 20% scored 0, 76% scored 1, 4% scored 2; *Delayed Implant placement + Xenograft + A‐PRF* = 20% scored 0, 68% scored 1, 12% scored 2, 20% scored 3	No statistically significant difference in pain scores
Yewale et al. (2021)^14^; Parallel RCT	20, 11/9, 35.15	AMOX (500 mg; t.i.d.; 5 d); IBU (400 mg; b.i.d.; 3 d); CHX (0.2%; b.i.d.; 14 d)	Alloplast + Collagen sponge (*n* = 10); Alloplast + A‐PRF + Collagen Sponge (*n* = 10)	1300 rpm (208 × *g*); 8 min; NR	Pain (VAS); Swelling (10 d)	*Alloplast + Collagen sponge* = 30% had mild pain, 70% had moderate pain; *Alloplast + A‐PRF + Collagen Sponge* = 20% had mild pain, 80% had moderate pain	No pain difference; less swelling in A‐PRF+
Asoka et al. (2022)^15^; Split‐mouth RCT	100, 44/46, NR	NR	Natural healing (*n* = 100); PRF (*n* = 100)	3000 rpm; 12 min; NR	Pain (VAS; 1, 3, 6 w)	*Natural healing* = 1 w: 1.40; *PRF* = 1 w: 1.45	No significant pain difference
Ghanaati et al. (2022)^16^; Parallel RCT	64, NR, NR	NR	Natural healing (*n* = 31); PRF (*n* = 33)	1200 rpm (177 × *g*); 8 min; Process for PRF, France	Pain (VAS; 1, 4, 7 d)	NR	No significance pain difference; trend favored PRF
Abad et al. (2023)^17^; Parallel RCT	27, 13/14, 57.56 ± 11.17	IBU (600 mg; t.i.d.) or PCT (1 g; t.i.d.; 7 d); CHX (0.12%; 7 d)	Natural healing (*n* = 31); L‐PRF (*n* = 33)	2700 rpm (408 × *g*); 12 min; Vacutainers: IntraSpin™, Intra‐Lock	Pain (VAS); Inflammation (1, 4 w)	*Natural healing* = 1 w: 2.38 ± 2.46; *L‐PRF* = 1 w: 3.78 ± 2.71	Similar pain and inflammation in both groups at 1 and 4 weeks
Baghel et al. (2023)^18^; Parallel RCT	20, NR, NR	NR	IIP + Alloplast (*n* = 10); IIP + i‐PRF (*n* = 10)	700 rpm; 3 min; NR	Pain (VAS; 1, 3, 6 d)	*IIP + Alloplast* = 1 d: 3.76 ± 0.68; 3 d: 2.12 ± 0.69; 6 d: 0.46 ± 0.34; *IIP + i‐PRF* = 1 d: 3.3 ± 0.47; 3 d: 2.2 ± 0.68; 6 d: 0.78 ± 0.34	No significant pain difference
Alasqah et al. (2024)^19^; Parallel RCT	60, NR, NR	NR	Natural healing (*n* = 20); PRF (*n* = 20); PRF + Collagen (*n* = 20)	2700 rpm; 12 min; Hermle Labortechnik, Germany	Pain (NRS; 1, 3, 7 d)	*Natural healing* = 1 d: 5.25 ± 1.91; 3 d: 1.80 ± 1.23; 7 d: 0.10 ± 0.44; *PRF* = 1 d: 2.90 ± 0.96; 3 d: 0.550 ± 0.60; 7 d: 0.0 ± 0.0; *PRF + Collagen* = 1 d: 2.70 ± 0.92; 3 d: 0.550 ± 0.75; 7 d: 0.0 ± 0.0	Both PRF groups had significantly reduced pain
Yu et al. (2024)^20^; Parallel RCT	31, 15/16, NR	Cefixime (100 mg) or Azithromycin (250 mg); 5 d; CHX (0.12%)	Natural healing (*n* = 15); DBBM‐C + PRF + RCM (*n* = 16)	3000 rpm; 10 min; NR	Pain (VAS; 1–3 d); Satisfaction (VAS; 4 m)	NR	PRF group had slightly higher satisfaction
Afifi et al. (2025)^21^; Parallel RCT	26, 10/16, 37.6 ± 3.5	AMOX (500 mg; t.i.d.; 7 d); IBU (600 mg; b.i.d.; 2 d); PRN	FGG (*n* = 13); PRF (*n* = 13)	2700–3000 rpm; 10–12 min; NR	Pain (VAS; 1 w, 6 m)	*FGG* = 1 w: 7.8 ± 0.8; *PRF* = 1 w: 2.4 ± 0.8	PRF had lower pain at week 1; equal at month 6
Aliyev et al. (2025)^22^; Parallel RCT	57, 30/27, 45 ± 5.6	PEN (650 mg; b.i.d.); Etodolac (400 mg; t.i.d.); CHX (0.2%)	Natural healing (*n* = 19); L‐PRF (*n* = 19); A‐PRF (*n* = 10)	*L‐PRF*: 3000 rpm; 10 min; IntraSpin *A‐PRF*: 1500 rpm; 14 min; IntraSpin	Pain (VAS); Swelling (1, 7 d)	*Natural healing* = 1 d: 7.0 ± 1.2; 7 d: 0.0 ± 0.0; *L‐PRF* 1 d: 3.0 ± 0.9; 7 d: 0.0 ± 0.0; *A‐PRF* = 1 d: 5.0 ± 1.0; 7 d: 0.0 ± 0.0	Both PRFs < control in pain and swelling
Amer et al. (2025)^23^; Parallel RCT	22, 10/12, Control: 34.5 ± 4.9; Test: 36.5 ± 10.8	AMOX (500 mg; t.i.d. 10 d or Doxycycline b.i.d.; 100 mg); IBU (600 mg; PRN); CHX (0.12%; b.i.d.; 14 d)	ADDG (*n* = 11); ADDG + i‐PRF (*n* = 11)	700 rpm (60 × *g*); 3 min; NR	Pain (VAS; 2 w); Satisfaction (Immediate postoperatively)	*ADDG* = 3.82 ± 1.33; *ADDG* + *i‐PRF* = 2.18 ± 1.17	i‐PRF had significantly less pain
Macedo et al. (2025)^24^; Parallel RCT	30, 23/7, 57.6 ± 10.2	AMOX (500 mg; t.i.d.; 7 d); Dipyrone (500 mg; QID; 3 d); CHX (0.12%; 14 d)	Natural healing (*n* = 67); A‐PRF (*n* = 67)	1300 rpm (208 × *g*); 14 min; Duo Quattro Choukroun PRF Centrifuge, France	Pain (VAS; 7, 14, 30, 60, 90, 120 d)	*Natural healing* = 7 d: 0.00 (0.00–1.50); 14 d: 0.0; 30 d: 0.0; 60 d: 0.0; 90 d: 0.0; 120 d: 0.0; *A‐PRF* = 7 d: 0.00; 14 d: 0.0; 30 d: 0.0; 60 d: 0.00; 90 d: 0.0; 120 d: 0.0	No significant difference in pain

Abbreviations: ADDG, autogenous demineralized dentin graft; AMOX, amoxicillin; A‐PRF, advanced platelet‐rich fibrin; AUG, augmentin; b.i.d., bis in die, twice per day; CHX, chlorhexidine; Clav, clavulanic acid; d, day; DBBM‐C, deproteinized bovine bone mineral with 10% collagen; FGG, Free gingival graft; h, hour; IBU, Ibuprofen; IIP, immediate implant placement; i‐PRF, injectable platelet‐rich fibrin; L‐PRF, leukocyte‐and platelet‐rich fibrin; m, month; min, minutes; NR, not reported; NRS, Numeric rating scale; NSAIDs, Nonsteroidal anti‐inflammatory drugs; PCT, paracetamol; PEN, penicillin; POP, plaster of paris; PRF, platelet‐rich fibrin; PRGF, plasma rich in growth factors; PRN, pro re nata, as needed; RCM, resorbable collagen membrane; RCT, randomized clinical trial; rpm, revolution per minute; t.i.d., ter in die, three times per day; T‐PRF, titanium‐prepared platelet‐rich fibrin; VAS, visual analog scale; w, week.

Three studies evaluated postoperative swelling,^86^, ^88^, ^91^ with two reporting a significant reduction in swelling in the PRF group,^86^, ^91^ while one study demonstrated no difference between groups.^33^ Of the two studies that evaluated postoperative analgesic use,^79^, ^80^ one demonstrated reduced intake in the PRF group,^80^ and the other found no statistically significant difference.^79^ Patient satisfaction was assessed by Yu et al.,^93^ who reported slightly higher satisfaction scores in the PRF + xenograft group compared with the natural healing group. Ten of the 24 studies reported postoperative use of CHX rinse,^77^, ^79^, ^80^, ^85^, ^86^, ^88^, ^91^, ^92^, ^95^, ^97^ while nine studies specified postoperative prescriptions for analgesics.^76^, ^77^, ^78^, ^79^, ^80^, ^81^, ^82^, ^85^, ^86^, ^88^, ^90^, ^91^, ^92^, ^95^, ^97^

Eighteen studies evaluated PRF as a stand‐alone biomaterial,^76^, ^77^, ^78^, ^79^, ^80^, ^81^, ^82^, ^83^, ^84^, ^87^, ^88^, ^90^, ^91^, ^94^, ^96^, ^97^, ^98^, ^99^ three evaluated PRF in combination with a xenograft,^77^, ^85^, ^93^ two evaluated PRF combined with an alloplastic graft,^76^, ^86^ one investigated PRF with dentin,^92^ and one explored PRF use during immediate implant placement.^89^

### 3.3 Palatal wound healing

A total of 20 clinical studies investigated the application of PRF in palatal wound healing (Table 3). Of these, 19 were RCTs,^100^, ^101^, ^102^, ^103^, ^104^, ^105^, ^106^, ^107^, ^108^, ^109^, ^110^, ^111^, ^112^, ^113^, ^114^, ^115^, ^116^, ^117^ and one was a prospective non‐RCT study.^118^ Only one study used a split‐mouth design,^105^ while the remaining 19 studies followed a parallel‐arm design.^100^, ^101^, ^102^, ^103^, ^104^, ^106^, ^107^, ^108^, ^109^, ^110^, ^111^, ^112^, ^113^, ^114^, ^115^, ^116^, ^117^, ^118^, ^119^ Regarding surgical indications, palatal tissue harvesting was performed for FGG procedures in 17 studies^100^, ^101^, ^102^, ^103^, ^104^, ^105^, ^106^, ^107^, ^108^, ^109^, ^110^, ^111^, ^112^, ^113^, ^114^, ^118^, ^119^ and for CTGs in three studies.^115^, ^116^, ^117^

**TABLE 3:** Summary of studies evaluating pain reduction with PRF for palatal wound healing.

Study; design	Sample size, M/F, mean age (years)	Postoperative medications	Control; test groups	PRF preparation (speed and time; Manufacturer)	Parameters assessed	Pain scores	Main findings
Free gingival grafts							
Femminella et al. (2016)^100^; Parallel RCT	40, 25/15, 32.4 ± 5.0	Ketoprofen PRN; AMOX/Clav (2 g; OD; 6 d); CHX (0.12%; b.i.d.; 21 d)	Gelatin sponge + Suture (*n* = 20); PRF + Suture (*n* = 20)	3000 rpm; 10 min; Intraspin Centrifuge, USA	Pain (VAS); changes in feeding habits (VAS); analgesic use; Alteration of sensitivity (VAS; 1, 2, 3 d, 4 w)	*Gelatin sponge + Suture*: 1 w: 4.50; 2 w: 3.50; 3 w: 2; 4 w: 1; *PRF + Suture*: 1 w: 2; 2 w: 2; 3 w: 1; 4 w: 0	PRF significantly reduced pain, feeding issues and analgesic use
Ustaoğlu et al. (2016)^101^; Parallel RCT	34, NR, NR	PCT (500 mg; PRN); CHX (0.12%; b.i.d.; 14 d)	Stent (*n* = 18); PRF + Stent (*n* = 16)	2800 rpm; 12 min; NR	Pain (VAS; 3, 7, 14, 21 d); analgesics use (1–7 d)	NR	No significant differences
Ozcan et al. (2017)^102^; Parallel RCT	125, NR, Control: 37.61 ± 6.64; Test 1: 34.55 ± 7.64; Test 2: 37.11 ± 4	NR	Spontaneous healing (*n* = 41); PRF + butyl cyanoacrylate (*n* = 42); butyl cyanoacrylate (*n* = 42)	2700 rpm; 10 min; Hettich Centrifuge, Germany	Pain (VAS; 5 h, 1–7, 14, 21, 28 d)	*Spontaneous healing*: 1 d: 6.10; 2 d: 5.22; 3 d: 3.22; 4 d: 2.41; 5 d: 1.98; 6 d: 1.29; 7 d: 1.02; 14 d: 0.32; 21 d: 0.15; 28d: 0.01; *Butyl cyanoacrylate*: 1 d: 4.55; 2 d: 3.90; 3 d: 1.90; 4 d: 1.21; 5 d: 0.90; 6 d: 0.12; 7 d: 0; 14 d: 0; 21 d: 0; 28d: 0; *PRF + Butyl cyanoacrylate*: 1 d: 2.00; 2 d: 1.29; 3 d: 0.35; 4 d: 0.12; 5 d: 0; 6 d: 0; 7 d: 0; 14 d: 0; 21 d: 0; 28d – 0	Significant pain reduction with PRF
Bahammam (2018)^103^; Parallel RCT	24, 14/10, Control: 28.5 ± 3.7; Test: 27.8 ± 4.3	ACT (1 g; PRN); CHX (0.12%; 14 d)	Noneugenol periodontal pack (*n* = 12); PRF + Noneugenol periodontal pack (*n* = 12)	3000 rpm (400 × *g*); 10 min; NR	Pain (VAS, NRS‐101, VRS‐4; 1‐8 h, then thrice daily on 2, 3, 4, 7 d)	*Noneugenol periodontal pack*: 1 h: 0.29; 2 h: 0.85; 3 h: 2.80; 4 h: 5.46; 5 h: 4.55; 6 h: 2.99; 7 h: 3.20; 8 h: 3.38; 2 d Noon: 2.93; 2 d PM: 3.02; 2 d AM: 3.94; 3 d Noon: 2.33; 3 d PM: 0.86; 3 d AM: 0.73; 4 d Noon: 0.53; 4 d PM: 0.34; 4 d AM: 0.10; 7 d Noon: 0.06; 7 d PM: 0.06; 7 d AM: 0; *PRF + Noneugenol periodontal pack*: 1 h: 0.21; 2 h: 0.58; 3 h: 0.94; 4 h: 2.10; 5 h: 2.36; 6 h: 2.31; 7 h: 1.25; 8 h: 1.41; 2 d Noon: 1.23; 2 d PM: 0.53; 2 d AM: 0.53; 3 d Noon: 0; 3 d PM: 0; 3 d AM: 0; 4 d Noon: 0; 4 d PM: 0; 4 d AM: 0; 7 d Noon: 0; 7 d PM: 0; 7 d AM: 0	Lower pain scores and faster recovery with PRF
İşler et al. (2019)^104^; Parallel RCT	60, 16/44, Control: 40.0 ± 15.7; Test 1: 40.4 ± 16.0; Test 2: 39.6 ± 6.2; Test 3: 37.0 ± 11.3; Test 4: 40.3 ± 11.3; Test 5: 39.7 ± 15.8	Flurbiprofen up to (100 mg; t.i.d.; 7 d); CHX (0.12%; b.i.d.; 21 d)	Spontaneous healing (*n* = 10); PRF (*n* = 10); Plastic retainer (*n* = 10); Ozone therapy (*n* = 10); LLLT (*n* = 10); Collagen Sponge (*n* = 10)	NR	Pain (VAS; 1–7 and 14 d); QoL (OHIP‐14; 14 d)	*Spontaneous healing*: 1 d: 2.2 ± 3.4; 2 d: 2.1 ± 3.0; 3 d: 0.3 ± 0.5; 4 d: 2.4 ± 2.8; 5 d: 3.9 ± 3.1; 6 d: 2.4 ± 3.2; 7 d: 1.9 ± 2.0; *LLLT*: 1 d: 1.9 ± 2.7; 2 d: 0.9 ± 1.4; 3 d: 1.1 ± 1.9; 4 d: 1.9 ± 2.8; 5 d: 1.5 ± 2.6; 6 d: 1.1 ± 2.8; 7 d: 0.6 ± 1.6; *Plastic retainer*: 1 d: 0.9 ± 2.0; 2 d: 0.6 ± 1.0; 3 d: 0.0 ± 0.0; 4 d: 1.0 ± 2.2; 5 d: 1.1 ± 2.1; 6 d: 1.1 ± 2.1; 7 d: 0.6 ± 1.6; *Ozone therapy*: 1 d: 0.9 ± 1.7; 2 d: 1.3 ± 1.9; 3 d: 0.9 ± 1.2; 4 d: 2.4 ± 3.0; 5 d: 4.0 ± 2.8; 6 d: 1.7 ± 1.9; 7 d: 0.6 ± 1.1; *Collagen Sponge*: 1 d: 1.3 ± 2.7; 2 d: 2.1 ± 2.9; 3 d: 0.7 ± 1.5; 4 d: 2.4 ± 3.1; 5 d: 2.8 ± 3.8; 6 d: 1.6 ± 2.9; 7 d: 1.6 ± 2.2; *PRF*: 1 d: 0.7 ± 1.5; 2 d: 0.3 ± 0.5; 3 d: 0.0 ± 0.0; 4 d: 0.1 ± 0.3; 5 d: 0.3 ± 0.7; 6 d: 0.1 ± 0.3; 7 d: 0.4 ± 1.0	Only 5 d showed significant difference for PRF
Patarapongsanti et al. (2019)^105^; Split‐mouth RCT	18, 7/11, 60.00 ± 8.45	AMOX (1.5 g; OD; 7 d); IBU (400 mg; PRN); CHX (0.12%; QID; 7 d)	Oxidized regenerated cellulose + Suture (*n* = 18); PRF + Suture (*n* = 18)	400 *g*; 10 min; Intraspin Centrifuge	Pain (VAS; 1, 3, 7 d)	*Oxidized regenerated cellulose + Suture*: All five patients felt pain; *PRF + Suture*: Only two patients felt pain	PRF group had less pain, particularly at 1 d
Hassan et al. (2020)^106^; Parallel RCT	30, 10/20, Control: 34.90 ± 8.18; Test 1: 30.90 ± 7.53 Test 2: 35.10 ± 7.09	Ketoprofen (150 mg; PRN); AUG (1 g; b.i.d.; 5 d); CHX (0.12%; b.i.d.; 21 d)	Gelatin sponge (*n* = 10); PRF (*n* = 10); Gelatin sponge + HA (*n* = 10)	3000 rpm; 10 min; NR	Pain (VAS; 1, 3, 7, 14, 21, 30 d)	*Gelatin sponge*: 1 d: 9.80 ± 0.42; 3 d: 7.50 ± 1.51; 7 d: 7.10 ± 1.20; 14 d: 5.20 ± 0.79; 21 d: 3.60 ± 0.97; 30 d: 1.50 ± 0.85; *Gelatin sponge + HA*: 1 d: 7.00 ± 2.94; 3 d: 6.20 ± 1.93; 7 d: 6.10 ± 2.23; 14 d: 2.30 ± 1.77; 21 d: 1.50 ± 1.27; 30 d: 0.70 ± 0.67; *PRF*: 1 d: 5.0 ± 1.89; 3 d: 3.30 ± 1.64; 7 d: 1.70 ± 2.26; 14 d: 0.60 ± 0.52; 21 d: 0.20 ± 0.63; 30 d: 0.00 ± 0.00	PRF group had greater pain reduction
Kızıltoprak and Uslu (2020)^107^; Parallel RCT	36, 9/27, Control: 32.08 ± 9.46; Test 1: 28.92 ± 9.66; Test 2: 33.25 ± 10.97	PCT (500 mg; PRN); CHX (0.12%; t.i.d.; 7 d)	Sterile aluminum foil and noneugenol periodontal pack (*n* = 12); i‐PRF + Sterile aluminum foil and noneugenol periodontal pack (*n* = 12); AFG + Sterile aluminum foil and noneugenol periodontal pack (*n* = 12)	2300 rpm (509.53 × *g*); 3 min; PC‐O2 Centrifuge, France	Pain (VAS; 3, 7, 14 d, 1 m)	*Sterile aluminum foil* and *noneugenol periodontal pack*: 3 d: 33.67 ± 20.99; 7 d: 33.33 ± 18.42; 14 d: 16.67 ± 16.48; 1 m: 0; *AFG + Sterile aluminum foil* and *noneugenol periodontal pack*; 3 d: 16.08 ± 16.37; 7 d: 11.67 ± 15.34; 14 d: 1.08 ± 3.75; 1 m: 0 ± 0; *i‐PRF + Sterile aluminum foil* and *noneugenol periodontal pack*: 3 d: 27.92 ± 14.38; 7 d: 32.42 ± 16.27; 14 d: 6.33 ± 9.31; 1 m: 0.83 ± 2.89	AFG group had the lowest pain at 7 d
Sharma et al. (2020)^108^; Parallel RCT	20, 5/15, NR	NR	Collagen membrane (*n* = 10); PRF (*n* = 10)	NR	Pain (VAS; 7, 12, 18, 24, 30 d)	Collagen membrane: 7 d: 3.2 ± 1.179; 12 d: 1.5 ± 1.65; 18d: 0.6 ± 0.699; 24 d: 0.5 ± 0.707; 30 d: 0.1 ± 0.316; PRF: 7 d: 1.9 ± 1.101; 12 d: 0.8 ± 0.789; 18d: 0.3 ± 0.483; 24 d: 0.4 ± 0.699; 30 d: 0.1 ± 0.316	PRF showed less pain, not statistically significant
Sousa et al. (2020)^109^; Parallel RCT	25, 9/16, 36.4 ± 14.9	PCT (1 g; t.i.d.)	Gelatin sponge + Protective splint (*n* = 11); A‐PRF + Protective splint (*n* = 14)	1500 rpm; 8 min; Duo Quattro Centrifuge, France	Pain (VAS; 2 d, 1, 2 w, 1, 3 m)	*Gelatin sponge + Protective splint*: 2 d: 2.0 (2); 1 w: 1.0 (2); 2 w: 0.0 (0); 1 m: 0.0 (0); 2 m: 0.0 (0); *PRF + Protective splint*: 2 d: 0.0 (1); 1 w: 0.0 (0); 2 w: 0.0 (0); 1 m: 0.0 (0); 2 m – 0.0 (0)	Control had more pain up to 14 d
Basma et al. (2022)^110^; Parallel RCT	72, 27/45, Control: 57.6 ± 18.4; Test 1: 64.2 ± 9.9; Test 2: 55.3 ± 14.8; Test 3: 52.0 ± 18.9	IBU (800 mg; pre‐op); IBU (600 mg; PRN); Warm salt water rinses 2–4 times daily; 14 d	Collagen plug + Suture (*n* = 18); PRF (*n* = 18); Collagen plug+Cyanoacrylate (*n* = 18); Palatal Stent (*n* = 18)	3000 rpm (635 × *g*); 10 min; Intraspin Centrifuge, USA	Pain (VAS), analgesic use; swelling, bleeding, activity tolerance (NRS; 1–14 d)	Average VAS scores = *Collagen plug + Suture*: 4.2 ± 2.4; *Collagen plug + Cyanoacrylate*: 2.2 ± 2.5; *Palatal Stent*: 1.0 ± 1.4; PRF: 1.9 ± 2.1	No pain difference, but PRF reduced analgesics
Eldien and Hassabou (2022)^111^; Parallel RCT	39, NR, NR	AUG (500 mg; b.i.d.; 5 d); IBU (600 mg); CHX (0.12%; b.i.d.; 28 d)	Noneugenol periodontal pack + Stent (*n* = 13); PRF + Stent (*n* = 13); Ozonated oil + Stent (*n* = 13)	3000 rpm; 10 min; NR	VAS (Pain; 1 d, 1, 2 w)	*Noneugenol periodontal pack + Stent*: 1 d: 9 ± 0.71; 1 w: 6.8 ± 0.84; *Ozonated oil + Stent*: 1 d: 7.6 ± 0.54; 1 w: 4 ± 0.71; *PRF + Stent*: 1 d: 2.2 ± 1.30; 1 w: 2 ± 1.41	PRF significantly reduced pain at 1 d and 1 w
Gatti et al. (2023)^112^; Parallel RCT	42, 18/24, Control: 38.4 ± 5; Test: 35.4 ± 6	AUG (2 g; pre‐op); then 1 g 6 h post‐op; then b.i.d.; 5 d; IBU PRN; CHX (0.12%; b.i.d.; 7 d)	Oxidized regenerated cellulose + Suture (*n* = 21); PRF + Suture (*n* = 21)	3000 rpm; 10 min; Hettich EBA 20 Centrifuge, Italy	Pain (VAS); analgesic use; discomfort, inability to chew, stress (VAS; 1 w)	NR	Only postoperative stress differed (less in PRF)
Mutallibli and Sağlam (2024)^118^; Parallel prospective	36, 11/25, Control: 43.91 ± 13.59; Test 1: 40.75 ± 12.74; Test 2: 37.00 ± 9.56	IBU (600 mg; 7 d); CHX (0.12%; 7 d)	Noneugenol periodontal pack + Stent (*n* = 12); L‐PRF (*n* = 12); A‐PRF (*n* = 12)	*L‐PRF*: 2700 rpm (635 × *g*); 12 min; Hettich EBA Centrifuge, Germany; *A‐PRF*: 1500 rpm (196 × *g*); 14 min; Hettich EBA Centrifuge, Germany	Pain (VAS); analgesic use (1–7 and 14 d); QoL (OHIP‐14; Weeks 1, 2)	*Noneugenol periodontal pack + Stent*: 1 d: 5.00 ± 3.61; 2 d: 4.16 ± 2.55; 3 d: 4.25 ± 2.63; 4 d: 3.58 ± 3.11; 5 d: 3.50 ± 3.06; 6 d: 2.50 ± 2.35; 7 d: 2.00 ± 2.00; 14 d: 0.41 ± 0.79; *L‐PRF*: 1 d: 4.95 ± 1.72; 2 d: 3.41 ± 2.02; 3 d: 3.16 ± 2.51; 4 d: 2.50 ± 1.97; 5 d: 1.75 ± 1.71; 6 d: 1.66 ± 1.49; 7 d: 1.00 ± 1.27; 14 d: 0.33 ± 0.65; *A‐PRF*: 1 d: 5.58 ± 2.90; 2 d: 4.75 ± 2.59; 3 d: 3.83 ± 2.03; 4 d: 3.08 ± 1.97; 5 d: 3.25 ± 2.52; 6 d: 3.00 ± 2.92; 7 d: 2.66 ± 2.99; 14 d: 1.16 ± 2.12	Pain similar, L‐PRF improved QoL at 2 w
Scott et al. (2024)^113^; Parallel RCT	71, 22/49, Control: 52.8 ± 16.1; Test: 55.9 ± 17.8	ACT (500 mg; pre‐op); IBU (600 mg); ACT (325 mg); IBU (600 mg; QID; 2 d; PRN)	Oxidized regenerated cellulose (*n* = 34); PRF (*n* = 37)	2700 rpm; 12 min; Intraspin Centrifuge, USA	Pain (NMRS‐21); analgesic use (1–3 d, 1–4 w)	*Oxidized regenerated cellulose*: 1 d: 4.25 ± 5.18; 2 d: 3.36 ± 3.87; 3 d: 3.03 ± 361; *PRF*: 1 d: 5.18 ± 4.28; 2 d: 3.77 ± 4.02; 3 d: 4.0 ± 4.44	No significant differences
Şen et al. (2024)^119^; Parallel RCT	39, 36/3, 37.76 ± 8.38	PCT (PRN); CHX (0.12%; t.i.d.; 14 d)	Palatal stent + Collagen sponge (*n* = 13); Palatal stent + L‐PRF (*n* = 13); Palatal stent + A‐PRF (*n* = 13)	*L‐PRF*: 2700 rpm; 12 min; NR; *A‐PRF*: 1500 rpm;14 min; NR	Pain, analgesic use, dietary changes, discomfort, bleeding (VAS; 1–7 and 14 d, 1 m); QoL (OHIP‐14; 1–7 and 14 d, 1, 6 m)	*Palatal stent + Collagen sponge*: 1 d: 70; 2 d: 56.15; 3 d: 64.61; 4 d: 52.3; 5 d: 46.92; 6 d: 45.38; 7 d: 46.15; 14 d: 25.38; 1 m: 14.61; *L‐PRF*: 1 d: 42.30; 2 d: 27.69; 3 d: 30.76; 4 d: 23.07; 5 d: 22.3; 6 d: 17.69; 7 d: 19.92; 14 d: 12.3; 1 m: 10; *A‐PRF*: 1 d: 70; 2 d: 51.53; 3 d: 37.69; 4 d: 30.76; 5 d: 30; 6 d: 26.15; 7 d: 20; 14 d: 13.84; 1 m: 10	PRF groups had better QoL and less pain, bleeding and analgesic use
Gulsever and Uckan (2025)^114^; Parallel RCT	26, 13/13, Control: 66.3 ± 10.7; Test: 57.1 ± 8.3	Naproxen sodium (550 mg; b.i.d.; 5 d); AUG (1 g; b.i.d.; 5 d); CHX (0.12%; t.i.d.; 7 d)	Palatal stent (*n* = 13); Palatal stent + PRF (*n* = 13)	3000 rpm (400 × *g*); 10 min; PC‐02 centrifuge, Process Ltd., France	Pain (VAS); burning sensation (VAS; 1 d, 1–4 w)	*Palatal stent*: 1 d: 4.69 ± 1.18; 1 w: 2.62 ± 1.26; 2 w: 1.23 ± 0.73; 3 w: 0.54 ± 0.78; 4 w: 0.15 ± 0.38; *Palatal stent + PRF*: 1 d: 0.85 ± 1.14; 1 w: 0.23 ± 0.44; 2 w: 0; 3 w: 0; 4 w: 0	PRF reduced pain and burning sensation at 1 d, 1 and 2 w
Subepithelial connective tissue grafts							
Alpan and Cin (2020)^115^; Parallel RCT	40, 19/21, Control: 30.89 ± 6.92; Test: 30.6 ± 6.45	PCT (500 mg; PRN); CHX (0.12%; b.i.d.; 7 d)	Spontaneous healing (*n* = 20); PRF + Suture (*n* = 20)	2800 rpm; 12 min; NR	Pain (VAS); analgesic use (1, 3, 7, 10 d)	*Spontaneous healing*: 1 d: 44.74 ± 19.54; 3 d: 38.42 ± 20.34; 7 d: 28.95 ± 31.70; 10 d: 14.21 ± 7.68; *PRF + Suture*: 1 d: 27 ± 11.74; 3 d: 20 ± 8.58; 7 d: 6.5 ± 7.45; 10 d: 3.0 ± 5.72	PRF group had significantly less pain and analgesic use
Mukhtar et al. (2023)^116^; Parallel RCT	31, 14/17, Control: 29.73 ± 7.95; Test: 30.27 ± 9.60	AMOX (500 mg; t.i.d.; 5 d); IBU (t.i.d.; 3 d); CHX (0.2%; 14 d)	Palatal Stent + LLLT (*n* = 16); Palatal Stent + PRF (*n* = 15)	3000 rpm; 12 min; REMI, Laboratories, India	Pain (VAS); burning sensation; discomfort (air spray test; 1, 4, 12 w)	*Palatal Stent + LLLT*: 1 w: 100% no pain/burning; *Palatal Stent +* PRF: 1 w: 26.67% no pain/burning	LLLT reduced pain and burning sensation more than the PRF group
Yadav et al. (2023)^117^; Parallel RCT	34, NR, NR	AMOX (500 mg; t.i.d.; 5 d); PCT (325 mg); DFN (50 mg; t.i.d.; 3 d)	Spontaneous healing (*n* = 17); PRF (*n* = 17)	3000 rpm; 12 min; NR	Pain, burning sensation (VAS); satisfaction (VAS; 10 d, 3, 12 w)	*Spontaneous healing*: 10 d: 76.5% with pain/burning; *PRF*: 10 d: 0% no pain/burning	PRF group reported less pain, burning and higher satisfaction

Abbreviations: ACT, acetaminophen; AFG, autologous fibrin glue; AMOX, amoxicillin; A‐PRF, advanced‐platelet‐rich fibrin; AUG, augmentin; b.i.d., bis in die, twice per day; CHX, chlorhexidine; Clav, clavulanic acid; DFN, diclofenac; h, hour; HA, hyaluronic acid; IBU, ibuprofen; i‐PRF, injectable platelet‐rich fibrin; LLLT, low‐level laser therapy; L‐PRF, leukocyte‐and platelet‐rich fibrin; m, month; min, minutes; NMRS‐21, 21‐point numerical scale; NR, not reported; NRS, numeric rating scale; NRS‐101, 101‐point numerical rating scale; OHIP‐14, oral health impact profile‐14; PCT, paracetamol; PRF, platelet‐rich fibrin; PRN, pro re nata, as needed; QID, quater in die, four times per day; RCT, randomized clinical trial; rpm, revolution per minute; t.i.d., ter in die, three times per day; VAS, visual analog scale; VRS‐4, 4‐point verbal rating scale; w, week.

Thirteen studies reported significantly lower postoperative pain in the PRF‐treated group compared with the control.^100^, ^102^, ^103^, ^104^, ^105^, ^106^, ^108^, ^109^, ^111^, ^114^, ^115^, ^117^, ^119^ Five studies reported no statistically significant difference in pain levels between PRF and control groups.^101^, ^110^, ^112^, ^113^, ^118^ Mukhtar et al.^116^ found greater postoperative pain in the PRF group compared with the low‐level laser therapy (LLLT) group. Kızıltoprak and Uslu^107^ reported that the acellular dermal matrix allograft (AFG) group experienced less pain than the i‐PRF group.^107^

Eight studies tracked postoperative analgesic use,^100^, ^101^, ^110^, ^112^, ^113^, ^115^, ^118^, ^119^ with four reporting reduced analgesic use in the PRF group.^100^, ^110^, ^115^, ^119^ CHX mouth rinse was prescribed postoperatively in 14 of the studies.^100^, ^101^, ^103^, ^104^, ^105^, ^106^, ^107^, ^111^, ^112^, ^114^, ^115^, ^116^, ^118^, ^119^ One study instructed patients to rinse with warm salt water postoperatively.^110^ In total, 18 of the 20 studies prescribed analgesic medications following surgery.^100^, ^101^, ^103^, ^104^, ^105^, ^106^, ^107^, ^109^, ^110^, ^111^, ^112^, ^113^, ^114^, ^115^, ^116^, ^117^, ^118^, ^119^

### 3.4 Mucogingival conditions

A total of 20 clinical studies were included that assessed the effects of PRF in various mucogingival procedures (Table 4). Eleven studies evaluated PRF for root coverage procedures,^120^, ^121^, ^122^, ^123^, ^124^, ^125^, ^126^, ^127^, ^128^, ^129^ six studies explored its application in gingivectomy or gingivoplasty procedures,^130^, ^131^, ^132^, ^133^, ^134^, ^135^ and three studies focused on vestibuloplasty or apically positioned flaps.^136^, ^137^, ^138^ Of these, 19 were RCTs,^120^, ^121^, ^122^, ^123^, ^124^, ^125^, ^126^, ^127^, ^128^, ^129^, ^130^, ^132^, ^133^, ^134^, ^135^, ^136^, ^137^, ^138^, ^139^ and one was a prospective cohort study.^131^ Thirteen studies used a split‐mouth design,^120^, ^121^, ^124^, ^125^, ^126^, ^128^, ^129^, ^130^, ^131^, ^132^, ^135^, ^136^, ^137^ while the remaining seven studies were parallel‐arm design.^122^, ^123^, ^127^, ^133^, ^134^, ^138^, ^139^

**TABLE 4:** Summary of studies evaluating pain reduction with PRF for mucogingival conditions.

Study; design	Sample size,M/F, mean age (years)	Postoperative medications	Control; test groups	PRF preparation (speed and time; Manufacturer)	Parameters assessed	Pain scores	Main findings
Root coverage							
Jankovic et al. (2010)^120^; Split‐mouth RCT	20, 8/12, NR	PEN (500 mg; QID; 14 d); IBU (800 mg; PRN); CHX (0.12%; b.i.d.; 14 d)	CAF + EMD (*n* = 10); CAF + PRF (*n* = 10)	3000 rpm (400 × *g*); 10 min; NR	Pain (VAS; 1–7 d)	*CAF + EMD*: 1 d: 1.06 ± 0.67; 2 d: 1.12 ± 0.43; 3 d: 0.95 ± 0.41; 4 d: 0.90 ± 0.35; 5 d: 0.82 ± 0.22; 6 d: 0.55 ± 0.10; 7 d: 0.25 ± 0.05; *CAF + PRF*: 1 d: 0.88 ± 0.64; 2 d: 0.80 ± 0.51; 3 d: 0.75 ± 0.45; 4 d: 0.61 ± 0.40; 5 d: 0.60 ± 0.33; 6 d: 0.50 ± 0.31; 7 d: 0.26 ± 0.27	PRF group reported significantly less pain in the first 5 d
Jankovic et al. (2012)^121^; Split‐mouth RCT	15, 5/10, NR	CHX (0.12%; 21 d)	CAF + CTG (*n* = 15); CAF + PRF (*n* = 15)	3000 rpm (400 × *g*); 10 min; NR	Pain (VAS; 1–7 d)	*CAF + CTG*: 1 d: 1.46 ± 0.51; 2 d: 1.33 ± 0.48; 3 d: 1.20 ± 0.41; 4 d: 1.06 ± 0.45; 5 d: 0.80 ± 0.41; 6 d: 0.60 ± 0.51; 7 d: 0.46 ± 0.51; *CAF + PRF*: 1 d: 0.46 ± 0.64; 2 d: 0.40 ± 0.50; 3 d: 0.33 ± 0.48; 4 d: 0.33 ± 0.48; 5 d: 0.26 ± 0.46; 6 d: 0.25 ± 0.45; 7 d: 0.20 ± 0.41	PRF had significantly lower pain; CTG had more severe pain
Eren & Atilla (2014)^129^; Split‐mouth RCT	22, 9/13, 33.81 ± 12.58	NSAIDs (PRN)	CAF + CTG (*n* = 22); CAF + PRF (*n* = 22)	400 × *g*; 12 min; Nüve Laboratory Equipments, NF200, Turkey	Pain; swelling	*CAF + CTG*: 18.1% of patients experienced pain as a complication	Less pain and edema with PRF. CTG had higher complication rate
Mufti et al. (2017)^122^; Parallel RCT	32, 18/14, Control: 36.38 ± 7.907; Test: 37.56 ± 5.291	CHX (0.2%; b.i.d.)	CAF + CTG (*n* = 16); CAF + PRF (*n* = 16)	3000 rpm; 10 min; NR	Pain (VAS; 1–3 w)	*CAF + CTG*: 1 w: 1.12 ± 1.25; 2 w: 0.5 ± 0.89; 3 w: 0.13 ± 0.50; *CAF + PRF*: 1 w: 2.0 ± 1.03; 2 w: 0.62 ± 0.95; 3 w: 0.0 ± 0.0	PRF group had significantly less pain in 1 w
Culhaoglu et al. (2018)^123^; Parallel RCT	22, 10/12, 31.6 ± 7.2	Flurbiprofen (100 mg; PRN); CHX (14 d)	CAF + CTG (*n* = 21); CAF + 2 membranes of PRF (*n* = 21); CAF + 4 membranes of PRF (*n* = 21)	2700 rpm; 12 min; (PC‐02 machine, Process Ltd., France)	Pain (VAS; 1–7 d); analgesics use (NR)	*CAF + CTG*: 1 d: 66.19 ± 18.84; 2 d: 37.14 ± 21.19; 3 d: 25.71 ± 16.30; 4 d: 35.71 ± 17.48; 5 d: 25.71 ± 24.76; 6 d: 11.43 ± 15.82; 7 d: 6.19 ± 12.03; *CAF + 2 membranes of PRF*: 1 d: 23.33 ± 15.28; 2 d: 20.95 ± 11.36; 3 d: 10.00 ± 9.49; 4 d: 1.90 ± 6.02; 5 d: 1.43 ± 3.59; 6 d: 0.00 ± 0.00; 7 d: 0.00 ± 0.00; *CAF + 4 membranes of PRF*: 1 d: 19.05 ± 15.78; 2 d: 18.10 ± 11.67; 3 d: 7.62 ± 11.79; 4 d: 2.86 ± 9.02; 5 d: 2.38 ± 4.36; 6 d: 0.95 ± 3.01; 7 d: 0.00 ± 0.00	PRF groups had less pain and analgesics use than CTG
Aldana et al. (2021)^124^; Split‐mouth RCT	13, 4/9, 41.7 ± 9.11	Ketoprofen (100 mg; b.i.d.; 3 d starting 1 d prior); CHX (0.12%; t.i.d.; 28 d)	CAF + CTG (*n* = 17); CAF + L‐PRF (*n* = 17)	408 *g*; 12 min; IntraSpin, Intra‐Lock	Pain (VAS; 1–3 d); postoperative complications (24, 28, 72 h)	*CAF + CTG*: 1 d: 38.5 ± 22.6; 2 d: 15.5 ± 13.0; 3 d: 3.0 ± 4.8; *CAF + L‐PRF*: 1 d: 16 ± 13.7; 2 d: 5 ± 8.5; 3 d: 1.5 ± 3.4	PRF showed significantly less pain and complications on 1–3 d
Carrera et al. (2023)^125^; Split‐mouth RCT	14, 7/7, 35.0	AMOX (500 mg; t.i.d.; 7 d); Nimesulide (100 mg; b.i.d.); Dipyrone (500 mg; QID); CHX (0.12%; b.i.d.; 15 d)	Tunnel + CTG (*n* = 36); Tunnel + PRF (*n* = 36)	3000 rpm, 10 min, IntraSpin, Intra‐Lock® System, Germany	Pain (VAS; 7, and 15 d); dentin sensitivity (1, 3 m)	*Tunnel + CTG*: 7 d: 3.90 (1.22–7.65); 15 d: 0.55 (0.00–2.12); *Tunnel + PRF*: 7 d: 2.40 (0.87–3.92); 15 d: 1.00 (0.00–1.72)	PRF had lower pain, but not statistically significant
Sen and Oncu (2023)^126^; Split‐mouth RCT	20, 7/13, 43.15 ± 8.67	Flurbiprofen; CHX (0.12%)	CAF + CTG (*n* = 60); CAF + T‐PRF (*n* = 58)	3500 rpm; 15 min; NR	Pain (VAS); sensitivity; dissatisfaction (1, 3 7 d)	*CAF + CTG*: 1 d: 70.50 ± 27.99; 3 d: 60.00 ± 16.54; 7 d: 35.00 ± 10.51; *CAF + T‐PRF*: 1 d: 55.00 ± 15.72; 3 d: 39.00 ± 10.20; 7 d: 21.50 ± 4.96	PRF showed lower pain, sensitivity and higher satisfaction
Santamaria et al. (2023)^127^; Parallel RCT	60, 33/27, 32.4 ± 5.0	IBU (400 mg; PRN); AMOX/clav (2 g; OD; 6 d); CHX (0.12%; b.i.d.; 21 d)	CAF (*n* = 20); CAF + CTG (*n* = 20); CAF + L‐PRF (*n* = 20)	2700 rpm; 12 min; IntraSpin™, Intra‐Lock System Europa SpA, Italy	Pain (VAS; 14 d); analgesics use (NR)	NR	PRF groups had less pain and analgesic need than the other two groups
Al‐Barakani et al. (2024)^128^; Split‐mouth RCT	18, 12/6, 25.0	IBU (200 mg); CHX spray (0.12%; 14 d)	PST + RCM (*n* = 18); PST + A‐PRF (*n* = 18)	1500 rpm, 14 min, NR	Pain (NRS; 1‐4 d)	*PST + RCM*: 1 d: 5 patients had moderate pain, 13 patients had severe pain; 2 d: 4 patients had mild pain, 11 patients had moderate pain, 3 patients had severe pain; 3 d: four patients had no pain, 11 patients had mild pain, three patients had moderate pain; 4 d: 15 patients had no pain, three patients had mild pain; *PST + A‐PRF*: 1 d: eight patients had no pain, 10 patients had mild pain; 2 d: All 18 patients had no pain; 3 d: All 18 patients had no pain; 4 d: All 18 patients had no pain	PRF group had lower pain, particularly on 1–3 d
Barakat et al. (2024)^140^; Parallel RCT	23, 9/14, Control: 39.19 ± 7.24; Test: 35.13 ± 8.66	AMOX (500 mg; t.i.d.; 5 d); IBU (400 mg; PRN); CHX (0.12%; b.i.d.; 14 d)	CTG (*n* = 16); A‐PRF (*n* = 16)	NR	Pain (VAS; 3 d); analgesic use (NR)	Pain scores at the recipient site ranged between 1 and 7 with a median score of 3 for both groups with no significant difference between CTG and A‐PRF groups	No significant difference in pain; CTG group used more analgesic consumption
Gingivectomy/Gingivoplasty (Often for Pigmentation or other reasons)							
Debnath & Chatterjee (2018)^130^; Split‐mouth RCT	11, NR, NR	CHX (0.2%)	Periodontal dressing (*n* = 11); PRFM gel + Periodontal dressing (*n* = 11); PRF + Periodontal dressing (*n* = 11)	2800 rpm; 12 min; R‐4C, REMI Laboratory Instruments, India	Pain (VAS; 3, 5 d)	*Periodontal dressing*: 3 d: 18.20% moderate pain, 81.80% severe pain; 5 d: 100% moderate pain; *PRFM gel + Periodontal dressing*: 3 d: 54.50% no pain, 45.50% moderate pain; 5 d: 100% no pain; *PRF + Periodontal dressing*: 3 d: 54.50% no pain, 45.50% moderate pain; 5 d: 100% no pain	PRF and PRFM reduced pain and inflammation vs. control
Dahiya et al. (2019)^131^; Split‐mouth Prospective	12, NR, 23.5	IBU (400 mg; t.i.d.; 3 d) 0.2% CHX	Periodontal dressing (*n* = 12); PRF (*n* = 12)	NR	Pain (VAS; 3, 5 d)	*Periodontal dressing*: 3 d: 58.3% no pain, 41.7% moderate pain; 5 d: 100% no pain; *PRF*: 3 d: 16.7% moderate pain, 83.3% severe pain; 5 d: 100% moderate pain	PRF had significantly less pain on 3 and 5 d
Bozkurt & Uslu (2022)^132^; Split‐mouth RCT	19, 8/11, 21.37 ± 7.73	PCT (t.i.d.; 7 d); CHX (0.12%; t.i.d.; 7 d)	Periodontal dressing (*n* = 19); PRF + Periodontal dressing (*n* = 19); AFG + Periodontal dressing (*n* = 19); CGF + Periodontal dressing (*n* = 19)	3000 rpm (866.87 × *g*); 10 min; NR	Pain (VAS; 7, 14, 2 d)	NR	Platelet groups had less early pain, but no group comparisons made
Bahar et al. (2024)^133^; Parallel RCT	46, 23/23, 16.98 ± 3.27	PCT (500 mg); CHX (0.12%)	Periodontal dressing (*n* = 23); Periodontal dressing + i‐PRF (*n* = 23)	2300 rpm (509.53 × *g*); 3 min; Intraspin centrifugation device	Pain (VAS; 3, 7, 14, 21 d)	NR	i‐PRF improved wound healing, comfort and QoL
Gupta et al. (2024)^134^; Parallel RCT	30, NR, NR	Analgesics PRN; CHX (0.2%)	Periodontal dressing (*n* = 15); PRF + Periodontal dressing (*n* = 15)	NR	Pain (VAS; 3 d)	*Periodontal dressing*: 3 d: 6.7% mild pain, 20.0% moderate pain, 66.7% severe pain, 6.7% no pain; *PRF + Periodontal dressing*: 3 d: 6.7% mild pain, 26.7% moderate pain, 6.7% severe pain, 60.0% no pain	PRF had less pain on 3 d; 9/15 patients had no pain
Ibrahim et al. (2024)^135^; Split‐mouth RCT	10, 5/5, 25.2	NR	Spontaneous healing (*n* = 10); i‐PRF (*n* = 10)	700 rpm; 3 min; IntraSpin, Duo Quattro Centrifuge, Process for PRF, France	Pain (VAS; 1 d, 1 w); satisfaction (3 m)	*Spontaneous healing*: 50% reported mild pain, 40% reported moderate pain, 10% reported severe pain; *i‐PRF*: 60% reported mild pain, 40% reported moderate pain	i‐PRF group had more patients pain‐free on 1 d. No difference in satisfaction
Vestibulplasty/Apically positioned flaps							
Temmerman et al. (2018)^136^; Split‐mouth RCT	8, 4/4, 51.6 ± 7.1	PCT (1 g; t.i.d.; 7 d); CHX (0.12%); PerioAid Spray (b.i.d.; 7 d)	FGG (*n* = 8); L‐PRF (*n* = 8)	2700 rpm (408 × *g*); 12 min; Intraspin®; Intra‐Lock™, Boca Raton, USA	Pain (VAS; Every 4 h until 7 d)	NR	PRF group had consistently less pain at all timepoints
Torkzaban et al. (2023)^137^; Split‐mouth RCT	10, 6/4, 33.9 ± 11.13	IBU (400 mg; PRN); CHX (0.2%; b.i.d.; 28 d)	MARF (*n* = 10); MARF + PRF (*n* = 10)	2400 rpm; NR; Arman Teb Noor, Iran	Pain (VAS; 1 d)	*MARF*: 1 d: 3.40 ± 0.69 *MARF + PRF*: 1 d: 3.20 ± 0.91	No difference in pain between groups
Veluri et al. (2024)^138^; Parallel RCT	20, 8/12, NR	NR	FGG (*n* = 15); i‐PRF + MN (*n* = 15)	700 rpm (60 × *g*); 3 min; A‐PRF + centrifuge	Pain (VAS; 1 w)	*FGG*: 1 w: 5.0 ± 0.70; *i‐PRF + MN*: 1 w: 1.11 ± 0.60	i‐PRF + MN group had significantly less pain at 1 w

Abbreviations: AFG, autologous fibrin glue; AMOX, amoxicillin; A‐PRF, advanced‐platelet‐rich fibrin; AUG, augmentin; b.i.d., bis in die, twice per day; CAF, coronally advanced flap; CGF, concentrated growth factor; CHX, chlorhexidine; Clav, clavulanic acid; CTG, connective tissue graft; d, day; EMD, enamel matrix derivative; FGG, free gingival graft; HA, hyaluronic acid; IBU, ibuprofen; i‐PRF, injectable platelet‐rich fibrin; L‐PRF, leukocyte‐and platelet‐rich fibrin; m, month; MARF, modified apically repositioned flap; min, minutes; MN, microneedling; NR, not reported; NRS, numeric rating scale; NSAIDs, nonsteroidal anti‐inflammatory drugs; PCT, paracetamol; PEN, penicillin; PRF, platelet‐rich fibrin; PRFM, platelet‐rich fibrin matrix; PRN, pro re nata, as needed; PST, pinhole surgical technique; QID, quater in die, four times a day; RCM, Resorbable collagen membrane; RCT, randomized clinical trial; rpm, revolution per minute; t.i.d., ter in die, three times per day; tCTG, connective tissue graft from the maxillary tuberosity; T‐PRF, titanium‐platelet‐rich‐fibrin; VAS, visual analog scale; w, week.

Out of the 20 studies that evaluated pain, 17/20 (85%) reported significantly reduced pain levels in the PRF group compared with the control.^120^, ^121^, ^122^, ^123^, ^124^, ^126^, ^127^, ^128^, ^129^, ^130^, ^131^, ^132^, ^133^, ^134^, ^135^, ^136^, ^138^ Three studies found no significant difference in pain between groups.^125^, ^137^, ^139^ Eren and Atilla^129^ specifically reported that none of the patients in the PRF group experienced pain or edema postoperatively, while 18.1% of patients in the CTG group experienced pain and two reported edema. Additionally, Aladana et al.^124^ reported that the PRF group demonstrated fewer postoperative complications, including hematoma and swelling, compared with the CTG group.^124^

Four studies monitored analgesic use, all of which reported reduced analgesic intake in the PRF group.^123^, ^124^, ^127^, ^139^ One study evaluated patient satisfaction, reporting higher satisfaction in the PRF group.^126^ Postoperative dentin hypersensitivity was assessed in two studies evaluated^125^, ^126^; with one study reporting reduced hypersensitivity in the PRF group^126^ and one showing no significant differences between groups.^125^ Postoperative CHX mouth rinse was prescribed in 17 out of the 20 studies,^121^, ^122^, ^123^, ^124^, ^125^, ^126^, ^127^, ^128^, ^130^, ^131^, ^132^, ^133^, ^134^, ^136^, ^137^, ^139^ and analgesic medications in 15 studies.^120^, ^123^, ^124^, ^125^, ^126^, ^127^, ^128^, ^129^, ^131^, ^132^, ^133^, ^134^, ^136^, ^137^, ^139^

Regarding comparisons, 9 of the 11 root coverage studies compared PRF to a CTG,^121^, ^122^, ^123^, ^124^, ^125^, ^126^, ^127^, ^129^, ^139^ one study compared PRF to enamel matrix derivative (EMD),^120^ and one compared PRF to a resorbable collagen membrane.^128^ Among the gingivectomy/gingivoplasty studies, five compared PRF (with or without periodontal dressing) to periodontal dressing alone,^130^, ^131^, ^132^, ^133^, ^134^ while one compared PRF with spontaneous healing.^135^ In the gingival augmentation subgroup, one study compared PRF with FGG,^136^ another study compared PRF + microneedling with FGG,^138^ and one assessed PRF combined with a modified apically positioned flap (MARF) versus MARF alone.^138^

### 3.5 Periodontal/bone procedures

A total of five studies were included that assessed the use of PRF in various periodontal/bone procedures (Table 5). Three of these studies explored PRF in the context of guided bone regeneration (GBR),^141^, ^142^, ^143^ one focused on its application in periodontal flap surgery,^144^ and one study assessed PRF in nonsurgical periodontal therapy (NSPT).^145^ Among the included studies, four were RCTs,^142^, ^143^, ^144^, ^145^ and one was a prospective cohort study.^141^ Four studies utilized a parallel‐arm design,^141^, ^142^, ^143^, ^145^ while one study had a split‐mouth design.^144^

**TABLE 5:** Summary of studies evaluating pain reduction with PRF for periodontal/bone procedures.

Study; design	Sample size, M/F, mean age (years)	Postoperative medications	Control; test groups	PRF preparation (speed and time; manufacturer)	Parameters assessed	Pain scores	Main findings
Guided bone regeneration							
Barbu et al. (2016)^141^; Parallel prospective	24, 11/13, NR	Antibiotics (6 d); ACT (PRN)	Ramus Block + Autograft and Xenograft particulate + Pericardium membrane (*n* = 12); Ramus Block + Autograft particulate + PRF (*n* = 12)	NR	Pain; 2 w	*Ramus Block + Autograft* and *Xenograft particulate + Pericardium membrane*: 2 w: one patient presented with pain as a postoperative complication. *Ramus Block + Autograft particulate + PRF*: 2 w: two patients presented with pain as a postoperative complication	PRF groups had two patients of persistent pain. No pain reduction with PRF group
Hartlev et al. (2020)^142^; Parallel RCT	27, 15/12, Control: 52.3 Test: 47.9	Methylprednisone (16 mg; b.i.d.; 1 d); IBU (400 mg; QID; 7 d); PCT (1 g; QID; 7 d); CHX (0.12%; b.i.d.; 14 d)	Ramus Block + DPBB + Collagen membrane (*n* = 13); Ramus Block + PRF (*n* = 14)	1300 rpm (208 × *g*); 14 min; Duo Quattro, A‐PRF 12; Process for PRF, France	Pain (VAS; 1–24 h, and 1‐7 d)	*Ramus Block + DPBB + Collagen membrane*: Average pain score on day of surgery = 16.9 ± 14.6; *Ramus Block + PRF*: Average pain score on day of surgery = 11.7 ± 13.7	PRF group had significantly less pain on 1 d only; otherwise, pain scores were similar. Medication may have masked differences
Sun et al. (2021)^143^; Parallel RCT	80, 52/28, Control: 35.4 ± 10.8; Test: 38.9 ± 6.2	Clindamycin (3–5 d)	Flap Curettage + FDBA (*n* = 40); Flap Curettage + FDBA + PRF (*n* = 40)	3200 rpm; 12 min; Shandong Biobase Scientific Instrument Co., Ltd	Pain (Questionnaire; 1 d)	*Flap Curettage + FDBA*: 1 d: 11 patients reported no pain, 24 patients reported mild pain, five patients reported moderate pain; *Flap Curettage + FDBA*: 1 d: 26 patients reported type no pain, 14 patients reported type mild pain	PRF group reduced pain at 24 h postoperative in peri‐implantitis defect regeneration
Periodontal surgery							
Joseph et al. (2012)^144^; Split‐mouth RCT	15, 6/9, 29.47 ± 7.65	AMOX (500 mg; t.i.d.; 5 d); PCT (500 mg; t.i.d.; 3 d); CHX (0.2%; t.i.d.; 42 d)	Open Flap Debridement (*n* = 15); Open Flap Debridement + PRF (*n* = 15)	3000 rpm; 10 min; KW‐70, AlmicroTM Instruments, India	Pain (VAS; 1 w)	NR	The PRF group demonstrated statistically significant lower pain scores than the control group
Nonsurgical periodontal therapy							
Sherif et al. (2025)^145^; Parallel RCT	45, 21/24, Control: 34.9 ± 8.6; Test 1: 35.9 ± 7.6; Test 2: 33.7 ± 8.7	NR	Nonsurgical therapy (*n* = 15); Nonsurgical therapy + i‐PRF (*n* = 15); Nonsurgical therapy + i‐PRF + Vitamin C (*n* = 15)	700 rpm (60 × *g*); 3 min; 45° rotor angulation; SCILOGEX DM0412, USA	Pain (VAS; 2 and 3 d)	*Nonsurgical therapy*: 2 d: 4 (0–8); 3 d: 4 (0–8); *Nonsurgical therapy + i‐PRF*: 2 d: 0 (0–6); 3 d: 0 (0–6); *Nonsurgical therapy + i‐PRF + Vitamin C*: 2 d: 1 (0–4); 3 d: 0 (0–2)	Both i‐PRF groups showed significantly lower pain than SRP alone, particularly on 2 and 3 d

Abbreviations: ACT, acetaminophen; AMOX, amoxicillin; b.i.d., bis in die, twice per day; CHX, chlorhexidine; d, day; DPBB, deproteinized bovine bone; FDBA, freeze‐dried bone allograft; h, hour; IBU, Ibuprofen; min, minutes; NR, not reported; PCT, paracetamol; PRF, platelet‐rich fibrin; PRN, pro re nata, as needed; QID, quater in die, four times per day; RCT, randomized clinical trial; rpm, revolution per minute; t.i.d., ter in die, three times per day; VAS, visual analog scale; w, week.

Four of the studies demonstrated less pain in the PRF group. One study by Barbu et al. showed less occurrence of pain as a complication in the non‐PRF group.^141^ Two of the studies reported prescribing CHX mouth rinse postoperatively,^142^, ^144^ and three studies prescribed analgesic medications for postoperative pain management.^141^, ^142^, ^144^

### 3.6 Maxillary sinus lifts

A total of two clinical studies were included that examined the utilization of PRF for sinus lift procedures (Table 6). Both studies were RCTs with a parallel‐arm design.^146^, ^147^ Lv et al.^147^ compared lateral sinus elevation using xenograft bone and a collagen membrane to transcrestal sinus elevation with PRF. They demonstrated significantly reduced postoperative pain and swelling, along with a greater willingness to undergo the procedure again. In contrast, Gurler and Delilbasi^146^ assessed lateral sinus elevation using allograft material and compared the application of a collagen membrane versus PRF. Their findings reported no statistically significant differences in postoperative pain scores between the two groups. Both studies prescribed analgesic medications and CHX mouth rinses postoperatively.^146^, ^147^

**TABLE 6:** Summary of studies evaluating pain reduction with PRF for sinus lift procedures.

Study; design	Sample size, M/F, mean age (years)	Postoperative medications	Control; test groups	PRF preparation (speed and time; manufacturer)	Pain assessment	Pain scores	Main findings
Gurler and Delilbasi (2016)^146^; Parallel RCT	24, 14/10, 47.8	Cephalexin (500 mg; b.i.d.; 5 d); PCT (500 mg; b.i.d.; 5 d); CHX (b.i.d.; 15 d)	Lateral Sinus Floor Elevation + Allograft + Collagen Membrane (*n* = 12); Lateral Sinus Floor Elevation + Allograft + L‐PRF (*n* = 12)	2700 rpm; 12 min; IntraSpinTM, Intra‐Lock Int.‐Inc., Baco Raton, USA	Pain; swelling; sleeping; eating; phonetics; daily living; and missed workdays (Questionnaire; 8, 24, 48 and 72 h and 7 d)	*Lateral Sinus Floor Elevation + Allograft + Collagen Membrane*: 8 h: 1.7 ± 0.67; 24 h: 0.9 ± 0.57; 48 h: 0.9 ± 0.99; 72 h: 0.6 ± 1.26; 7 d: 0.5 ± 0.85; *Lateral Sinus Floor Elevation + Allograft + L‐PRF*: 8 h: 1.7 ± 0.95; 24 h: 0.8 ± 0.63; 48 h: 0.5 ± 0.71; 72 h: 0.4 ± 0.52; 7 d: 0.1 ± 0.32	L‐PRF group showed improvements, but differences were not statistically significant
Lv et al. (2022)^147^; Parallel RCT	40, 18/22, 52.75	PEN and Metronidazole injections immediate post‐op; the lateral sinus floor elevation group received DEX (10 mg) and Vit‐C (1 g) injections OD; 3 d; CHX (t.i.d.; 3 d)	Lateral Sinus Floor Elevation + Xenograft + Collagen Membrane (*n* = 20); Transcrestal Sinus Floor Elevation + PRF (*n* = 20)	3000 rpm; 10 min; IntraSpin, Intra‐Lock, USA	Pain (VAS); swelling (VRS; 0, 1, 2, 3 and 7 d); Willingness to do procedure again (VRS; 7 d)	*Lateral Sinus Floor Elevation + Xenograft + Collagen Membrane*: 0 d: 35.0 (32.5–37.0); *Transcrestal Sinus Floor Elevation + PRF*: 0 d: 18.0 (15.0–22.5)	Transcrestal PRF group had less pain and sdwelling, with higher willingness for repeat procedures compared with lateral + Xenograft group

Abbreviations: b.i.d., bis in die, twice per day; CHX, chlorhexidine; d, day; DEX, dexamethasone; h, hour; L‐PRF, leukocyte‐and platelet‐rich fibrin; min, minutes; NR, not reported; OD, once daily; PCT, paracetamol; PEN, penicillin; PRF, Platelet‐rich fibrin; RCT, randomized clinical trial; rpm, revolution per minute; t.i.d., ter in die, three times per day; VAS, visual analog scale; Vit‐C, vitamin C; VRS, verbal rating scale.

### 3.7 Endodontic procedures

A total of 15 studies were included that investigated the use of PRF in various endodontic procedures (Table 7). Among these, six studies focused on PRF use in root‐end surgery,^148^, ^149^, ^150^, ^151^, ^152^, ^153^ five studies evaluated PRF in pulp revitalization procedures,^154^, ^155^, ^156^, ^157^, ^158^ and four studies assessed its use in pulpotomies.^159^, ^160^, ^161^, ^162^ Eleven of the included studies were RCTs,^148^, ^149^, ^151^, ^153^, ^154^, ^155^, ^156^, ^157^, ^159^, ^161^, ^162^ two were prospective cohort studies,^150^, ^160^ and two were retrospective analyses.^152^, ^158^ Fourteen studies followed a parallel‐group design,^148^, ^149^, ^150^, ^151^, ^152^, ^153^, ^154^, ^155^, ^157^, ^158^, ^159^, ^160^, ^161^, ^162^ while only one study used a split‐mouth design.^156^

**TABLE 7:** Summary of studies evaluating pain reduction with PRF for endodontic procedures.

Study; design	Sample size, M/F, mean age (years)	Postoperative medications	Control; test groups	PRF preparation (speed and time; manufacturer)	Parameters assessed	Pain scores	Main findings
Root‐end surgery							
Angerame et al. (2015)^148^; Parallel RCT	11, 5/6, 46.81	AMOX (1 g; b.i.d.; 6 d); Analgesics (PRN)	RES (*n* = 4); RES + PRF (*n* = 7)	2500 rpm; 10 min; NR	Pain (VAS); swelling (VAS; 2, 6 and 12 h and 1–7 d)	NR	PRF group had reduced pain and swelling at 2–6 h
Meschi et al. (2018)^149^; Parallel RCT	50, 22/28, Control: 49.7 ± 16.34; Test 1: 44.8 ± 13.87; Test 2: 44.9 ± 19.25; Test 3: 43.5 ± 16.37	Azithromycin (500 mg; t.i.d.); PCT (1 g; t.i.d.); IBU (600 mg; t.i.d.); CHX (0.12%; 7 d)	RES (*n* = 15); RES + Collagen Membrane (*n* = 10); RES + PRF (*n* = 12); RES + Collagen Membrane + PRF (*n* = 13)	702 × *g*; NR; Intra‐Lock® Int., USA	Patient (VAS); Quality of life (Questionnaire;1–7 d)	Mean pain over 7 days: RES: 1.4 ± 1.98; RES + Collagen Membrane: 1.8 ± 1.91; RES + PRF: 0.8 ± 1.04; RES + Collagen Membrane + PRF: 1.2 ± 0.94	No significant pain or quality of life difference between groups
Singh et al. (2020)^150^; Parallel Prospective	126, NR, NR	Antibiotics and analgesics	Hydroxyapatite granules (*n* = 42); Cerament (*n* = 42); PRF (*n* = 42)	NR	Pain; (1, 3, 6, 9 and 12 m)	Mean days taken to get over pain: Hydroxyapatite granules: 51.2 d; Cerament: 52.3 d; PRF: 44.7 d	PRF group had the shortest pain resolution (i.e., 44.7 d)
Soto‐Penaloza et al. (2020)^151^; Parallel RCT	50, 22/28, Control: 49.2 ± 14.4; Test: 48.2 ± 17.6	IBU (400 mg; t.i.d.; 3 d); CHX (0.12%; b.i.d.; 7 d)	RES (*n* = 25); RES + A‐PRF (*n* = 25)	1300 rpm (208 × *g*); 8 min; Process for PRF, France	Pain (VAS); Analgesic use; Quality of Life (Questionnaire; 1–7 d)	Mean pain over 7 days: RES: 20.7 ± 16.3; RES + PRF: 12.7 ± 8.5	A‐PRF group had lower pain and better quality of life; not statistically significant
Machut & Zoltowska (2022)^152^; Parallel Retrospective	36, NR, 31.85	NR	RES + Calcium hydroxide (*n* = 20); RES+A‐PRF+ (*n* = 20)	1200 rpm; 8 min; Neuation iFuge D06 Premium Edition, Neuation Technologies Pvt., India	Pain (VAS; 7 d)	RES + Calcium hydroxide: 7–11 d patients had no pain, five patients had mild pain, two patients had moderate pain, and two patients had severe pain. RES+A‐PRF+: 7–14 d patients had no pain, 4 patients had mild pain, and two patients had moderate pain	More pain in control; not statistically significant
Gulsever et al. (2025)^153^; Parallel RCT	64, 30/34, Control 1: 32 ± 13; Test 1: 34 ± 15; Control 2: 31 ± 12; Test 2: 33 ± 11	AMOX/clav (1 g; b.i.d.; 5 d); Naproxen sodium (550 mg; b.i.d.); CHX (0.2%; t.i.d.; 7 d)	Retrograde obturation (*n* = 16); Orthograde obturation (*n* = 16); Retrograde obturation + L‐PRF (*n* = 16); Orthograde obturation + L‐PRF (*n* = 16)	3000 rpm (400 × *g*); 10 min; PC‐02 centrifuge, Process Ltd., France	Pain (VAS); swelling; percussion tenderness and palpation; 1 w and 1, 3, 6, 9 and 12 m; analgesic use; 1–7 d	Retrograde obturation: 7 d: 3 ± 3; Orthograde obturation: 7 d: 3 ± 3; Retrograde obturation + L‐PRF: 7 d: 2 ± 3; Orthograde obturation + L‐PRF: 7 d: 3 ± 3	RG + L‐PRF group had significantly less percussion tenderness and analgesic use
Pulp revitalization							
Shivashankar et al. (2017)^154^; Parallel RCT	60, 32/28, NR	NR	Induced bleeding (*n* = 20); PRP (*n* = 20); PRF (*n* = 20)	NR	Pain; 12 m	No patients reported with pain after 12 m	All groups pain‐free at 12 m
Ragab et al. (2019)^155^; Parallel RCT	22, 15/7, 9.86 ± 1.55	NR	Natural blood clot (*n* = 11); Natural blood clot + PRF (*n* = 11)	3000 rpm; 12 min; Centrifuge Model 801, China	Pain; 12 m	NR	No significant difference in pain
Rizk et al. (2020)^156^; Split‐mouth RCT	12, 6/6, 9.08 ± 1.165	NR	Natural blood clot (*n* = 12); PRF (*n* = 12)	2400 rpm; 12 min; i Fuge D06 Bench Top Doctor Centrifuge, Neuation Tech Pvt. Ltd., India	Pain; swelling; 3, 6, 9 and 12 m	NR	No significant difference in pain/swelling
Rizk et al. (2020)^157^; Parallel RCT	25, 13/12, Control: 9.08 ± 1.038; Test: 9.08 ± 1.165	NR	PRP (*n* = 13); PRF (*n* = 12)	NR	Pain; swelling; 3, 6, 9 and 12 m	NR	No significant difference in pain/swelling
Thakkar et al. (2023)^158^; Parallel Retrospective	28, NR, NR	NR	Natural blood clot (*n* = 14); PRF (*n* = 14)	NR	Pain; swelling; 3 and 6 m	No patients had a presence of pain after treatment	No significant difference in pain/swelling
Pulpotomy							
Kumar et al. (2016)^159^; Parallel RCT	41, NR, Control: 17.82; Test 1: 21.20; Test 2: 25.81	NR	MTA (*n* = 15); Calcium hydroxide (*n* = 14); PRF (*n* = 13)	NR	Pain (NRS; 1 and 7 d, and 6 and 12 m)	*MTA*: 1 d: 0.66 ± 0.84; 7 d: 0.83 ± 1.22; 6 m: 0.52 ± 1.06; 12 m: 0.13 ± 0.35; *Calcium hydroxide*: 1 d: 0.56 ± 0.89; 7 d: 1.00 ± 2.06; 6 m: 0.64 ± 1.33; 12 m: 0.38 ± 0.65; *PRF*: 1 d: 0.42 ± 0.64; 7 d: 0.14 ± 0.36; 6 m: 0.50 ± 1.34; 12 m: 0.30 ± 0.63	No significance difference in pain
Singh et al. (2020)^160^; Parallel prospective	60, NR, NR	NR	Calcium Hydroxide (*n* = 20); MTA (*n* = 20); PRF (*n* = 20)	NR	Pain (NRS; 0, 1 and 7 d and 1, 3, 6 and 12 m)	*Calcium Hydroxide*: 1 d: 1.2; 1 w: 0.54; 1 m: 0.67; 3 m: 0.65; 6 m: 0.45; 12 m: 0.41; *MTA*: 1 d: 1.4; 1 w: 0.62; 1 m: 0.68; 3 m: 0.61; 6 m: 0.42; 12 m: 0.40; *PRF*: 1 d: 1.1; 1 w: 0.52; 1 m: 0.54; 3 m: 0.60; 6 m: 0.51; 12 m: 0.38	No significant difference in pain
Doranala et al. (2021)^161^; Parallel RCT	60, 33/27, 25.0	NR	Calcium Hydroxide (*n* = 20); EndoSequence (*n* = 20); EndoSequence + T‐PRF (*n* = 20)	NR	Pain (VAS; 1–7 d and 6 and 12 m)	T‐PRF and EndoSequence showed the maximum reduction of pain mean score (0.22 ± 0.39)	EndoSequence + T‐PRF group had the greatest pain reduction
Abuhashema et al. (2025)^162^; Parallel RCT	108, 43/65, Control: 26.85 ± 8.94; Test: 24.63 ± 6.20	NR	Pulp cap + MTA (*n* = 54); Pulp + PRF (*n* = 54)	3000 rpm; 10 min; 800D, Biofield medical, China	Pain (VPIS); pain on Percussion (+ve or −ve); 6 and 12 m	*Pulp cap + MTA*: 6 m: 75.93% of patients had an absece of pain, 16.67% had weak pain, 7.41% had moderate pain; 12 m: 75.93% had no pain, 24.07% had weak pain; *Pulp cap + PRF*: 6 m: 74.07% of patients had an absece of pain, 25.93% had weak pain; 12 m: 74.07% had no pain, 25.93% had weak pain	No statistically significant difference at 6 m; PRF group was pain‐free at 12 m

Abbreviations: AMOX, amoxicillin; A‐PRF, advanced‐platelet‐rich fibrin; A‐PRF+, advanced‐platelet‐rich fibrin plus; AUG, augmentin; b.i.d., bis in die, twice per day; CHX, chlorhexidine; Clav, clavulanic acid; d, day; IBU, ibuprofen; L‐PRF, leukocyte‐and platelet‐rich fibrin; m, months; min, minutes; MTA, mineral trioxide aggregate; NR, not reported; NRS, numeric rating scale; OD, once daily; PCT, paracetamol; PRF, platelet‐rich fibrin; PRN, pro re nata, as needed; PRP, platelet‐rich plasma; RCT, randomized clinical trial; RES, root‐end surgery; rpm, revolution per minute; t.i.d., ter in die, three times per day; T‐PRF, titanium‐prepared platelet‐rich fibrin; VAS, visual analog scale; VPIS, verbal pain intensity scale; w, week.

Regarding pain outcomes, four studies reported a significant pain reduction in the PRF group compared with non‐PRF controls,^148^, ^150^, ^161^, ^162^ while 11 studies found no statistically significant differences in postoperative pain between the groups.^149^, ^151^, ^152^, ^153^, ^154^, ^155^, ^156^, ^157^, ^158^, ^159^, ^160^ Six studies considered pain as a clinical criterion for procedural success.^150^, ^154^, ^155^, ^156^, ^157^, ^158^

Moreover, five studies evaluated swelling,^148^, ^153^, ^156^, ^157^, ^158^ with only one study reporting reduced swelling in the PRF group.^148^ Notably, three of these studies evaluated swelling as part of the clinical success criteria.^156^, ^157^, ^158^ Two studies evaluated QoL outcomes,^149^, ^151^ and one study found significantly improved QoL in the PRF group.^151^ Gulsever et al.^153^ examined postoperative percussion tenderness and palpation and reported significantly reduced percussion tenderness in the L‐PRF + retrograde obturation (RG) group compared to RG alone. One study monitored analgesic use, reporting reduced analgesic intake in the PRF group.^153^ CHX was prescribed postoperatively in three studies,^149^, ^151^, ^153^ while five studies prescribed postoperative analgesics.^148^, ^149^, ^150^, ^151^, ^152^

### 3.8 Orthodontic procedures

A total of four studies were included that investigated the application of PRF in orthodontic procedures (Table 8). Among these, three studies focused on PRF use during canine retraction,^163^, ^164^, ^165^ while one study assessed PRF in the context of accelerated orthodontics.^166^ All four studies were RCTs,^163^, ^164^, ^165^, ^166^ while three used a split‐mouth design,^163^, ^164^, ^165^ and one used a parallel‐arm design.^166^

**TABLE 8:** Summary of studies evaluating pain reduction with PRF for orthodontic procedures.

Study; design	Sample size, M/F, mean age (years)	Postoperative medications	Control; treatment groups	PRF preparation (speed and time; manufacturer)	Parameters assessed	Pain scores	Main findings
Canine retraction							
Gupta et al. (2023)^163^; Split‐mouth RCT	16, 9/7, 21.85 ± 2.45	None	Spontaneous healing (*n* = 16); L‐PRF (*n* = 16)	2700 rpm (400 × *g*); 12 min; IntraSpin centrifuge system, Intra‐Lock Int. Boca, Raton, USA	Pain (four‐point Likert scale); swelling (four‐point Likert scale); discomfort (four‐point Likert scale); 21 and 42 d; analgesic use (NR)	NR	No significant difference in pain. No analgesics used by either group
Zeitounlouian et al. (2023)^164^; Split‐mouth RCT	21, NR, 20.9 ± 3.9	None	Spontaneous healing (*n* = 21); i‐PRF (2 injections with 1 month interval) (*n* = 21)	700 rpm; 3 min; HW6C, HWLAB® Mini Combo Centrifuge, China	Pain (VAS, Questionnaire); discomfort; swelling; (1, 2, 6, 24 and 48 h after 2nd injection)	*Spontaneous healing*: 1 h: 3.80 ± 8.64; 2 h: 2.38 ± 7.68; 6 h: 1.42 ± 6.54; 24 h: 0.47 ± 2.18; *i‐PRF injections*: 1 h: 24.28 ± 17.58; 2 h: 15.23 ± 15.69; 6 h: 5.23 ± 10.30; 24 h: 2.38 ± 8.89	Experimental group had higher pain and swelling for first 6 h, then normalized by 24 h
Satapathy et al. (2024)^165^; Split‐mouth RCT	16, 6/10, 22.25 ± 2.26	NR	Saline (*n* = 16); L‐PRF (*n* = 16)	2700 rpm; 12 min; NR	Pain (Pain Index Scores; 3, 7 and 15 d)	*Saline*: 3 d: 5.43 ± 0.62; 7 d: 3.87 ± 0.81; 15 d: 2.00 ± 0.63; *L‐PRF*: 3 d: 4.18 ± 0.83; 7 d: 3.06 ± 0.99; 15 d: 1.31 ± 0.87	Pain was significantly lower on L‐PRF side at all timepoints
Accelerated orthodontics							
Taha et al. (2023)^166^; Parallel RCT	25, 0/25, NR	NR	No injection (*n* = 10); i‐PRP (*n* = 8); i‐PRF (*n* = 7)	700 rpm; 3 min; NR	Pain (VAS; 1 w)	*No injection*: 1 w: 100% Pain; *i‐PRP*: 1 w: 68.85% Pain; *i‐PRF*: 1 w: 79.42% Pain	Both i‐PRP and i‐PRF reduced pain vs. control; i‐PRP had the greatest pain relief

Abbreviations: d, day; h, hour; i‐PRF, injectable platelet‐rich fibrin; i‐PRP, injectable platelet‐rich plasma; L‐PRF, leukocyte‐and platelet‐rich fibrin; min, minutes; NR, not reported; RCT, randomized clinical trial; rpm, revolution per minute; VAS, visual analog scale; w, week.

Two of the included studies demonstrated reduced pain in the PRF group compared with the control,^165^, ^166^ while one study found no statistically significant difference in pain levels between PRF and non‐PRF groups.^163^ Interestingly, Zeitounlouian et al.^164^ reported an initial increase in pain and swelling in the PRF group within the first 6 h postoperatively, which subsequently declined sharply to baseline levels by 24 h.

### 3.9 Oral lesions

A total of six studies evaluated the use of PRF in the management of various oral lesions (Table 9). Among these, four studies focused specifically on oral lichen planus (OLP),^167^, ^168^, ^169^, ^170^ one study evaluated PRF in premalignant oral lesions,^171^ and one study investigated PRF for treating mucocutaneous disorders (i.e., Steven–Johnson syndrome, mucous membrane pemphigoid, and pemphigus vulgaris).^172^ Of the six included studies, three had a parallel‐arm design,^170^, ^171^, ^172^ while the remaining three utilized a split‐mouth design.^167^, ^168^, ^169^

**TABLE 9:** Summary of studies evaluating pain reduction with PRF for oral lesions.

Study; design	Sample size, M/F, mean age (age)	Postoperative medications	Control; test groups	PRF preparation (device)	Pain assessment	Pain scores	Main findings
Lichen planus							
Bennardo et al. (2021)^167^; Split‐ mouth RCT	9, 3/6, 59.56 ± 3.57	NR	TA injections (*n* = 9); i‐PRF injections (*n* = 9)	700 rpm; 3 min; Process for PRF, France	Pain (VAS); symptoms (Pain, discomfort, burning sensation, dry mouth) (Questionnaire; 0 d and 1, 2, 3, 4 and 8 w)	*TA injections*: 8 w: 1.9 ± 1.5; *i‐PRF injections*: 8 w: 2.9 ± 2.1	No significant difference between TA and i‐PRF in symptom reduction
Saglam et al. (2021)^168^; Split‐mouth RCT	24, 10/14, 52.25	NR	Methylprednisone injections (*n* = 24); i‐PRF injections (*n* = 24)	700 rpm (47 × *g*); 3 min; Process for PRF, Boca Raton, USA	Pain (VAS; 0 and 45 d and 1, 2 and 6 m); Quality of life (OHIP‐14; 0 d and 1, 2 and 6 m)	*Methylprednisone injections*: 45 d: 41.25 ± 23.97; 1 m: 20.83 ± 17.61; 2 m: 12.29 ± 14.37; 6 m: 23.33 ± 26.81; *i‐PRF injections*: 45 d: 37.92 ± 25.66; 1 m: 19.79 ± 18.15; 2 m: 8.75 ± 10.96; 6 m: 13.33 ± 18.34	Both treatments improved pain and quality of life significantly, with no significant differences between the groups
Al‐Hallak et al. (2023)^169^; Split‐ mouth RCT	12, 3/9, 48 ± 12.7	NR	TA injections (*n* = 12); i‐PRF injections (*n* = 12)	700 rpm (60 × *g*); 3 min; Process for PRF, France	Pain and Burning (VAS; 1, 15 d and 4 w)	*TA injections*: 1 d: 6.33 ± 2.26; 15 d: 3 ± 1.53; 4 w: 0.58 ± 0.79; *i‐PRF injections*: 1 d: 6.08 ± 2.15; 15 d: 3.87 ± 1.28; 4 w: 1.91 ± 1.31	No significant difference in pain and burning between TA and i‐PRF groups
El Araby et al. (2025)^170^; Parallel RCT	30, 7/23, 42	NR	TA injections (*n* = 10); MMF gel (*n* = 10); i‐PRF (*n* = 10)	700 rpm; 3 min; NR	Pain and Burning (VAS; 0 d, 2, 4, 6 and 8 w)	*TA injections*: 0 d: 9.57 ± 0.53; 2 w: 7.57 ± 0.53; 4 w: 5.57 ± 0.53; 6 w: 4.57 ± 0.53; 8 w: 2.71 ± 0.49; *MMF gel*: 0 d: 9.57 ± 0.53; 2 w: 8.57 ± 0.53; 4 w: 6.57 ± 0.53; 6 w: 5.57 ± 0.53; 8 w: 4.57 ± 0.53; *i‐PRF*: 0 d: 9.57 ± 0.53; 2 w: 7.00 ± 0.82; 4 w: 4.14 ± 0.69; 6 w: 2.00 ± 0.82; 8 w: 0.29 ± 0.49	i‐PRF group showed significantly lower pain scores at all timepoints
Premalignant oral lesions							
Mahajan et al. (2018)^171^; Parallel RCT	30, 27/3, Control: 37.93 ± 18.00; Test: 38.46 ± 13.16	Aceclofenac‐serratiopeptidase (PRN)	Collagen Membrane (*n* = 15); PRF (*n* = 15)	3000 rpm (400 × *g*); 10 min; NR	Pain (NRS; 7, 15, 30 and 60 d)	*Collagen membrane*: 7 d: 100% of patients had moderate pain; 15 d: 46.67% of patients had slight pain; 30 d: 60% of patients had no pain; 60 d: 100% of patients had no pain; *PRF*: 7 d: 93.33% of patients had moderate pain; 15 d: 66.66% of patients had slight pain; 30 d: 86.67% of patients had no pain; 60 d: 100% of patients had no pain	PRF group had significantly less pain at 15 and 30 d; no difference at 7 and 60 d
Oral Ulcers from SJS, BMMP, and PV							
Ahmed et al. (2022)^172^; Parallel RCT	16, NR, NR	Systemic steroid treatment	Clobetasol Propionate + Orabase (0.05%; *n* = 8); PRF + Orabase (*n* = 8)	3000 rpm; 15 min; NR	Pain (NRS; 7 d)	*Clobetasol Propionate + Orabase*: 7 d: 25% of patients had 40% pain reduction, 25% had 20% reduction and 50% had almost no change in pain score; *PRF + Orabase*: 7 d: 87% of patients had 100% pain reduction. 13% had 90% pain reduction	PRF group had 100% pain reduction; control had 32.5% reduction at 7 d

Abbreviations: BMMP, benign mucous membrane pemphigoid; d, day; i‐PRF, injectable platelet‐rich fibrin; m, month; min, minutes; MMF, mycophenolate mofetil; NR, not reported; NRS, Numeric rating scale; OHIP‐14, 14‐item oral health impact profile; PRF, platelet‐rich fibrin; PRN, pro re nata, as needed; PV, pemphigus vulgaris; RCT, randomized clinical trial; rpm, revolution per minute; SJS, Steven–Johnson syndrome; TA, triamcinolone acetonide; VAS, visual analog scale; w, week.

Three studies reported significantly reduced pain scores in the PRF‐treated group compared with the control.^170^, ^171^, ^172^ In contrast, three studies, mainly in OLP, found no statistically significant difference in pain outcomes between PRF and control groups.^167^, ^168^, ^169^

### 3.10 Alveolar osteitis (Dry socket)

A total of eight studies explored the application of PRF for the management of alveolar osteitis (dry socket; Table 10). Among these, six studies were RCTs,^173^, ^174^, ^175^, ^176^, ^177^, ^178^ while the remaining two were prospective clinical studies.^179^, ^180^ Seven of the included studies used a parallel‐arm design,^173^, ^174^, ^175^, ^176^, ^177^, ^178^, ^180^ while one employed a split‐mouth design.^179^

**TABLE 10:** Summary of studies evaluating pain reduction with PRF for alveolar osteitis (dry socket).

Study; design	Sample size, M/F, mean age (years)	Postoperative medications	Control; treatment groups	PRF preparation (speed and time; manufacturer)	Parameters assessed	Pain scores	Main findings
Hussain et al. (2018)^173^; Parallel RCT	40, 9/31, 35.2	IBU (400 mg; PRN); PCT (325 mg; PRN)	ZOE (*n* = 20); PRF (*n* = 20)	3000 rpm; 10 min; NR	Pain (VAS; 0, 1, 3, 5 and 7 d)	*ZOE*: 0 d: 8.87 ± 0.915; 1 d: 5.07 ± 1.223; 3 d: 3.47 ± 1.767; 5 d: 0.87 ± 1.685; 7 d: 0.27 ± 0.704; *PRF*: 0 d: 9.87 ± 0.352; 1 d: 9.47 ± 0.743; 3 d: 5.67 ± 1.718; 5 d: 3.00 ± 1.134; 7 d: 0.60 ± 0.737	ZOE group had faster pain relief than PRF, significant on 1, 3, and 5 d
Yuce and Komerik (2019)^174^; Parallel RCT	40, 18/22, 31.2	Diclofenac sodium (50 mg; PRN)	Alveolar curettage (*n* = 20); Alveolar curettage+A‐PRF+ (*n* = 20)	1300 rpm (200 × *g*); 8 min; PRF Duo Centrifuge, France	Pain (VAS; 0, 1, 3, 5 and 7 d)	*Alveolar curettage*: 0 d: 6.8 ± 0.83; 1 d: 7.25 ± 1.02; 3 d: 7.05 ± 1.23; 5 d: 5.9 ± 0.91; 7 d: 4.05 ± 0.76; *Alveolar curettage + A‐PRF+*: 0 d: 7.15 ± 1.04; 1 d: 5.2 ± 1.06; 3 d: 2.25 ± 0.64; 5 d: 0.8 ± 0.62; 7 d: 0.45 ± 0.51	A‐PRF+ group had significantly lower pain at all timepoints
Keshini et al. (2020)^175^; Parallel RCT	30, NR, NR	PCT (500 mg; t.i.d.)	Alvogyl dressing (*n* = 15); PRF (*n* = 15)	3000 rpm; 10 min; Remy C‐852	Pain (VAS; 1, 3 and 10 d)	*Alvogyl dressing*: 1 d: 9.6667 ± 0.48795; 3 d: 1.1333 ± 0.83381; 10 d: 0.0000 ± 0.00000; *PRF*: 1 d: 9.5333 ± 0.51640; 3 d: 0.2667 ± 0.457; 10 d: 0.0000 ± 0.00000	Both groups showed significant pain reduction by 3 d; comparable results
Reeshma and Dain (2021)^176^; Parallel Prospective	70, 17/53, NR	NR	ZOE (*n* = 35); PRF (*n* = 35)	3500 rpm; 15 min; Remi clinical centrifuge C‐854/4, Remi Elektrotechnik Limited, India	Pain (VAS; 1, 3, 5 and 7 d)	*ZOE*: 1 d: 7.4 ± 1.5; 3 d: 5.1 ± 1.1; 5 d: 3.4 ± 0.9; 7 d: 2.1 ± 0.7; *PRF*: 1 d: 4.1 ± 1.2; 3 d: 2.6 ± 0.9; 5 d: 1.7 ± 0.9; 7 d: 0.8 ± 0.8	PRF group had greater pain reduction with statistical significance
Chybicki and Janas‐Naze (2022)^179^; Split‐mouth Prospective	30, 18/12, 37.8	NR	Aspirin cones (*n* = 30); PRF (*n* = 30)	2690 rpm; 12 min; Hettich EBA 200	Pain (VAS; 0–2 d)	*Aspirin cones*: 1 d: 1.07 ± 1.17; 2 d: 2.0 ± 1.49; *PRF*: 1 d: 1.1 ± 0.96; 2 d: 2.10 ± 1.12	PRF showed significantly better pain relief on 1 and 2 d
Wang (2023)^177^; Parallel RCT	60, 21/39, Control: 34.77 ± 9.91; Test: 32.17 ± 9.59	Cephalosporin and metronidazole (t.i.d.); (Azithromycin in case of allergy); Loxoprofen sodium (60 mg; PRN)	Iodoform gauze (*n* = 30); PRF (*n* = 30)	1500 rpm; 8 min; NR	Pain (VAS; 0, 3 and 7 d); analgesic use; 0–7 d	*Iodoform gauze*: 0 d: 8.33 ± 0.80; 3 d: 4.17 ± 1.49; 7 d: 1.73 ± 1.44; *PRF*: 0 d: 8.67 ± 0.76; 3 d: 1.10 ± 1.03; 7 d: 0.30 ± 0.60	PRF group had significantly lower pain and less analgesic use on 3 and 7 d
Yuzbasioglu and Eroglu (2024)^180^; Parallel Prospective	80, 30/50, A‐PRF: 43.25 ± 15.50; LLLT: 35 ± 11.46; PTX: 29.14 ± 8.44; Alveogyl: 37.45 ± 13.04	PCT (500 mg; QID)	Alveogyl dressing (*n* = 20); A‐PRF (*n* = 20) LLLT (*n* = 20); PTX (*n* = 20)	1500 rpm; 14 min; Hettich EBA 20 Zentrifuger, D‐78532, 2006, Germany	Pain (VAS; 0, 2, 4, 7 and 14 d)	*Alveogyl dresssing*: 0 d: 7.05 ± 1.638; 2 d: 5.15 ± 2.084; 4 d: 3.60 ± 1.818; 7 d: 1.50 ± 1.051; *LLLT*: 0 d: 7.40 ± 1.789; 2 d: 4.75 ± 1.860; 4 d: 3.00 ± 2.026; 7 d: 1.35 ± 0.988; *PTX*: 0 d: 7.70 ± 1.174; 2 d: 5.85 ± 1.531; 4 d: 4.05 ± 1.395; 7 d: 1.85 ± 1.387; *A‐PRF*: 0 d: 8.05 ± 1.638; 2 d: 5.40 ± 2.280; 4 d: 3.25 ± 2.074; 7 d: 1.50 ± 1.051	A‐PRF group showed the greatest pain reduction, but not statistically significant
Tekin and Turedi (2025)^178^; Parallel RCT	60, 28/32, 37.40 ± 12.81	NR	Alveolar curettage (*n* = 30); Alveolar curettage + i‐PRF (*n* = 30)	700 rpm; 3 min; NR	Pain (VAS; 0, 3 and 7 d)	*Alveolar curettage*: 0 d: 6.87; 3 d: 4.40; 7 d: 2.27; *Alveolar curettage + i‐PRF*: 0 d: 6.43; 3 d: 3.67; 7 d: 1.27	i‐PRF group had significantly lower pain on 3 and 7 d

Abbreviations: A‐PRF, advanced‐platelet‐rich fibrin; A‐PRF+, advanced‐platelet‐rich fibrin plus; d, day; IBU, ibuprofen; i‐PRF, injectable platelet‐rich fibrin; LLLT, low‐level laser therapy; min, minutes; NR, not reported; PCT, paracetamol; PRF, platelet‐rich fibrin; PRN, pro re nata, as needed; PTX, pentoxifylline; QID, quater in die, four times a day; RCT, randomized clinical trial; rpm, revolution per minute; t.i.d., ter in die, three times per day; VAS, visual analog scale; ZOE, zinc oxide eugenol.

Five studies reported a reduction in pain levels in the PRF group compared with the control or non‐PRF‐treated group.^174^, ^176^, ^177^, ^178^, ^179^ Two studies reported no significant difference in pain outcomes between the groups.^175^, ^180^ Hussain et al.^173^ found that while PRF was effective, zinc oxide eugenol (ZOE) dressing resulted in faster pain relief. Four of the included studies reported prescribing postoperative analgesics,^173^, ^174^, ^175^, ^177^ though the correlation between analgesic use and pain scores was not consistently analyzed.

### 3.11 Oroantral communications

A total of five studies assessed the use of PRF in the management of oroantral communications (Table 11). All included studies were RCTs with a parallel‐arm design.^181^, ^182^, ^183^, ^184^, ^185^ Four of the five studies reported significantly reduced postoperative pain in the PRF group compared with non‐PRF‐treated groups.^181^, ^182^, ^184^, ^185^ However, Kaba et al.^183^ reported higher pain scores in the PRF group when compared with groups treated with oxidized regenerated cellulose or an oral wound dressing.

**TABLE 11:** Summary of studies evaluating pain reduction with PRF for oroantral communications.

Study; design	Sample size, M/F, mean age (years)	Postoperative medications	Control; treatment groups	PRF Preparation (speed and time; manufacturer)	Parameters assessed assessment	Pain scores	Main findings
Bilginaylar (2019)^181^; Parallel RCT	36, 20/16, NR	PCT (500 mg; PRN)	Buccal advancement flap (*n* = 15); Buccal advancement flap + PRF (*n* = 21)	3000 rpm; 10 min; Elektro‐mag, M415P, Turkey	Pain (VAS); analgesics use; swelling; 1, 2, 3 and 7 d	*Buccal advancement flap*: Sum of pain scores – 59.0 ± 19.53 *Buccal advancement flap + PRF*: Sum of pain scores – 31.24 ± 15.89	PRF group had significantly less pain, swelling and analgesic
Hunger et al. (2023)^182^; Parallel RCT	40, 27/13, Control: 51.6 ± 10.9; Test: 47.0 ± 14.9	AMOX/clav (875 mg/125 mg) or Clindamycin (600 mg; t.i.d.; 7 d); IBU (600 mg; PRN); CHX (0.12%; b.i.d.; 5 d); Fentrinol (b.i.d.; 5 d)	Buccal advancement flap (*n* = 20); PRF (*n* = 20)	1300 rpm (210 × *g*); 8 min; NR	Pain (VAS); analgesics use; 1–7 d	NR	PRF group had significantly lower pain and reduced analgesic use
Kaba et al. (2024)^183^; Parallel RCT	60, 30/30, 34.65 ± 12.73	AMOX/clav (1 g; 7 d); Flurbiporfen (100 mg; 7 d); CHX (7 d)	Oxidized regenerated cellulose + SG (*n* = 20); OWD (*n* = 20); PRF (*n* = 20)	NR	Pain (VAS; 1, 3, and 7 d)	*Oxidized regenerated cellulose + SG*: 1 d: 2.5 ± 1.82; 3 d: 1.25 ± 1.25; 7 d: 0.10 ± 0.45; *OWD*: 1 d: 1.05 ± 1.43; 3 d: 0.3 ± 0.66; 7 d: 0 ± 0; *PRF*: 1 d: 4.35 ± 2.85; 3 d: 2.55 ± 1.73; 7 d: 0.70 ± 0.92	PRF group had significantly higher pain scores than OWD and SG groups. on all days evaluated
Śmieszek‐Wilczewska et al. (2024)^184^; Parallel RCT	22, 13/9, 42.45	IBU and/or PCT and antibiotics; CHX (0.12%)	Buccal advancement flap (*n* = 11); PRF (*n* = 11)	1200 rpm (177 × *g*); 8 min; Process for PRF, France	Pain (VAS; 0, 1, 7, and 14 d)	*Buccal advancement flap*: 0 d: 6.1; 1 d: 2.7; 7 d: 0.3; 14 d: 0; *PRF*: 0 d: 4.2; 1 d: 1.7; 7 d: 0; 14 d: 0	PRF group had significantly more pain reduction at 24 h and 7 d than control
Mahmoud (2025)^185^; Parallel RCT	24, 14/10, NR	IBU (600 mg; t.i.d.); AUG (1 g; b.i.d.; 5 d); Otrivin nasal decongestant (0.05%)	Buccal fat pad advancement (*n* = 6); Fibrin Glue (*n* = 6); Oxidized regenerated cellulose plugs (*n* = 6); A‐PRF (*n* = 6)	3000 rpm; 15 min; NR	Pain (VAS); swelling; 1, 3, and 7 d, 1–4 w	*Buccal fat pad advancement*: 1 d: 8.83 ± 0.75; 3 d: 5.67 ± 1.51; 1 w: 3.67 ± 0.82; 2 w: 2.00 ± 1.26; 3 w: 0.67 ± 0.82; 4 w: 0.00 ± 0.00; *Fibrin Glue*: 1 d: 7.83 ± 0.75; 3 d: 4.17 ± 0.75; 1 w: 2.17 ± 0.41; 2 w: 0.67 ± 0.52; 3 w: 0.50 ± 0.55; 4 w: 0.00 ± 0.00; *Oxidized regenerated cellulose plugs*: 1 d: 7.00 ± 0.89; 3 d: 4.00 ± 1.55; 1 w: 2.67 ± 0.52; 2 w: 2.00 ± 1.10; 3 w: 0.00 ± 0.00; 4 w: 0.00 ± 0.00; *A‐PRF*: 1 d: 6.83 ± 0.75; 3 d: 3.83 ± 0.75; 1 w: 2.33 ± 0.52; 2 w: 1.00 ± 0.63; 3 w: 0.17 ± 0.41; 4 w: 0.00 ± 0.00	A‐PRF showed the lowest pain at 1 w and minimal swelling across time

Abbreviations: AMOX, amoxicillin; A‐PRF, advanced‐platelet‐rich fibrin; AUG, augmentin; b.i.d., bis in die, twice per day; CHX, chlorhexidine; Clav, clavulanic acid; d, day; IBU, ibuprofen; min, minutes; NR, not reported; OWD, oral wound dressing; PCT, paracetamol; PRF, platelet‐rich fibrin; PRN, pro re nata, as needed; RCT, randomized clinical trial; rpm, revolution per minute; SG, sterile gauze; t.i.d., ter in die, three times per day; VAS, visual analog scale; w, week.

Two studies evaluated postoperative swelling, and both reported decreased swelling in the PRF‐treated group compared with controls.^181^, ^185^ Two studies evaluated analgesic use postoperatively, and both reported a reduction in analgesic use in the PRF group.^181^, ^182^ Concerning postoperative care, three studies reported prescribing CHX rinses,^182^, ^183^, ^184^ and four studies reported prescribing analgesic medications.^181^, ^182^, ^183^, ^184^, ^185^

### 3.12 Medication‐related osteonecrosis of the jaw

A total of three studies evaluated the use of PRF in the management of medication‐related osteonecrosis of the jaw (MRONJ; Table 12). Of these, one study was an RCT,^186^ one was a prospective clinical study,^187^ and one was a retrospective cohort study.^188^ All three studies used a parallel‐group design.^186^, ^187^, ^188^

**TABLE 12:** Summary of studies evaluating pain reduction with PRF for medication‐related osteonecrosis of the jaw.

Study; design	Sample size, M/F, mean age (years)	Postoperative medications	Control; test groups	PRF preparation (speed and time; manufacturer)	Parameters assessed	Pain scores	Main findings
Giudice et al. (2018)^186^; Parallel RCT	47, 23/24, 74.7 ± 6.5	AMOX (1 g; b.i.d.) or Clindamycin (600 mg; t.i.d.; 10 d; starting 3 d pre‐op); Metronidazole (250 mg; t.i.d.; 10 d; starting 3 d preo‐op); CHX (0.2%)	Bone surgery (*n* = 23); Bone surgery + PRF (*n* = 24)	1300 rpm; 8 min; Process for PRF, France	Pain (VAS; 1 and 6 m and 1y)	*Bone Surgery*: 1 m: 3.73; 6 m: 1.78; 1 y: 1.43; *Bone Surgery + PRF*: 1 m: 2.66; 6 m: 1.5; 1 y: 1.42	PRF group showed significantly lower pain at 1 m only; no difference later
Tenore et al. (2020)^188^; Parallel retrospective	34, 8/26, 58.09	AMOX/clav acid (1 g; b.i.d.; 7 d; starting 3 d pre‐op); CHX (0.2%; t.i.d.; starting 3 d pre‐op)	Necrotic bone removal (*n* = 21); Necrotic bone removal + PBM (*n* = 21); Necrotic bone removal + PBM + L‐PRF (*n* = 21)	2700 rpm (408 × *g*); 12 min; IntraLock Int., Boca Raton, USA	Absence of Pain; (3 and 6 m)	*Necrotic bone removal*: 3 m: 100% of sites healing with an absence of pain; 6 m: 50% of sites healing (including absence of pain); *Necrotic bone removal + PBM*: 3 m: 46.2% of sites healing (including absence of pain); 6 m: 38.5% of sites healing (including absence of pain); *Necrotic bone removal + PBM + L‐PRF*: 3 m: 100% of sites healing (including absence of pain); 6 m: 100% of sites healing (including absence of pain)	L‐PRF group had significantly better healing (including pain absence) at 3 and 6 m
Blatt et al. (2022)^187^; Parallel prospective	45, 18/27, 71.5 ± 8.6	Clindamycin (600 mg; 5 d); analgesics applied individually	Necrotic bone removal (*n* = 20); Necrotic Bone Removal + PRF (*n* = 25)	1200 rpm (177 × *g*); 8 min; A‐PRF+; Process for PRF, France	Pain (VAS); OHRQoL (OHIP G14); 5, 14 and 42 d	NR	No statistically significant difference in pain or OHRQoL between groups

Abbreviations: AMOX, amoxicillin; b.i.d., bis in die, twice per day; CHX, chlorhexidine; Clav, clavulanic acid; d, day; L‐PRF, leukocyte‐and platelet‐rich fibrin; m, month; min, minutes; NR, not reported; OHIP, oral health impact profile; OHRQoL, oral health‐related quality of life; PBM, photobiomodulation; PRF, platelet‐rich fibrin; RCT, randomized clinical trial; rpm, revolution per minute; t.i.d., ter in die, three times per day; VAS, visual analog scale; y, year.

Two of the studies demonstrated significantly reduced postoperative pain in the PRF group compared with the non‐PRF or control groups.^186^, ^188^ The remaining study observed no significant difference in pain outcomes between the PRF and control groups.^187^ Tenore et al.^188^ found significantly improved healing in the PRF group compared with both PBM and control groups at 3 and 6 months post‐treatment. Although pain was not analyzed as an isolated outcome, the authors included the absence of pain as a critical criterion for defining lesion resolution, indirectly supporting PRF's analgesic effect. Postoperative CHX rinses were prescribed in two studies,^186^, ^188^ while only one study explicitly reported prescribing analgesics postoperatively.^187^

### 3.13 Temporomandibular joint disorders

A total of 18 studies assessed the use of PRF in the management of TMJ disorders (Table 13). Of these, 13 studies investigated PRF for internal derangement,^189^, ^190^, ^191^, ^192^, ^193^, ^194^, ^195^, ^196^, ^197^, ^198^, ^199^, ^200^, ^201^ three studies focused on noninflammatory degenerative joint disorders (osteoarthritis),^202^, ^203^, ^204^ one study evaluated PRF for TMJ hypermobility,^205^ and one study examined PRF in patients with unspecified TMJ pain.^206^ Among these, 12 studies were RCTs,^189^, ^190^, ^191^, ^193^, ^194^, ^195^, ^198^, ^199^, ^200^, ^203^, ^205^, ^206^ five were retrospective analyses,^192^, ^197^, ^201^, ^202^, ^204^ and one was a prospective clinical trial.^196^ All studies utilized a parallel‐group design.

**TABLE 13:** Summary of studies evaluating pain reduction with PRF in temporomandibular joints.

Study; design	Sample size, M/F, mean age (years)	Postoperative medications	Control; test groups	PRF Preparation (speed and time; manufacturer)	Pain assessment	Pain scores	Main findings
Internal derangement disorder							
Teama & Abdelmoneim (2020)^189^; Parallel RCT	30, NR, Control: 29 ± 6.5; Test: 30 ± 8	Analgesics	Arthrocentesis + saline (*n* = 18); Arthrocentesis + i‐PRF (V18)	3300 rpm; 2 min; NR	Pain (VAS; 0 d, 1, 2, 4, and 8 w)	*Arthrocentesis + saline*: 0 d: 8 ± 1.5; 2 w: 5.5 ± 1.5; 4 w: 1.5 ± 1.5; 8 w: 0.5 ± 0.5; *Arthrocentesis + i‐PRF*: 0 d: 8 ± 1.5; 2 w: 3 ± 2; 4 w: 0.5 ± 0.5; 8 w: 0 ± 0	i‐PRF group showed significantly reduced pain
Yuce and Komerik (2020)^174^; Parallel retrospective	47, NR, NR	Ketoprofen (500 mg; t.i.d.; 3 d)	Arthrocentesis (*n* = 16); ArthroVcentesis + HA injection (*n* = 14); Arthrocentesis + i‐PRF (*n* = 17)	700 rpm (60 × *g*); 3 min; NR	Pain (VAS; 0 d, 2 w and 1, 2, 3, 6, 9 and 12 m)	*Arthrocentesis*: 0 d: 66.69 ± 8.75; 2 w: 36.25 ± 8.06; 1 m: 31.88 ± 6.29; 2 m: 26.88 ± 5.12; 3 m: 24.06 ± 6.38; 6 m: 23.44 ± 9.44; 9 m: 26.56 ± 9.26; 12 m: 29.69 ± 11.9; *Arthrocentesis + HA*: 0 d: 64.64 ± 11.17; 2 w: 24.64 ± 4.99; 1 m: 21.07 ± 5.25; 2 m: 18.93 ± 3.50; 3 m: 17.86 ± 4.26; 6 m: 17.5 ± 4.70; 9 m: 20.36 ± 4.58; 12 m: 23.21 ± 6.39; *Arthrocentesis + i‐PRF*: 0 d: 65 ± 12.75; 2 w: 26.47 ± 7.24; 1 m: 22.35 ± 7.52; 2 m: 19.41 ± 5.56; 3 m: 17.65 ± 4.72; 6 m: 15.59 ± 4.96; 9 m: 16.18 ± 4.16; 12 m: 14.71 ± 4.13	i‐PRF + arthrocentesis showed superior, gradual pain relief
Ghoneim et al. (2021)^190^; Parallel RCT	40, 11/29, Control: 28.60 ± 8.42; Test: 26.45 ± 8.21	AMOX/clav Potassium (1 g; b.i.d.; 5 d)	Arthrocentesis (*n* = 20); Arthrocentesis + i‐PRF (*n* = 20)	700 rpm (60 × *g*); 3 min; Spin plus centrifuge	Pain (VAS; 0 d, 1 w and 3 and 6 m)	*Arthrocentesis*: 0 d: 8.0 (3.0–9.0) 1 w: 5.0 (1.0–8.0) 3 m: 3.0 (1.0–6.0) 6 m: 3.0 (1.0–5.0) *Arthrocentesis + i‐PRF*: 0 d: 6.0 (4.0–10.0) 1 w: 0.0 (0.0–2.0) 3 m: 0.0 (0.0–0.0) 6 m: 0.0 (0.0–2.0)	i‐PRF led to significantly greater pain reduction
Karadayi and Gursoytrak (2021)^191^; Parallel RCT	36, 19/17, 39.82	NR	Arthrocentesis (*n* = 18); Arthrocentesis + i‐PRF (*n* = 18)	700 rpm; 3 min; NR	Pain (VAS; 0, 10, 30 d, and 3 m)	*Arthrocentesis*: 0 d: 5.94 ± 1.67; 10 d: 4.61 ± 2.46; 30 d: 3.61 ± 2.38; 3 m: 3.00 ± 2.01; *Arthrocentesis + i‐PRF*: 0 d: 6.22 ± 2.63; 10 d: 2.78 ± 2.42; 30 d: 1.00 ± 1.75; 3 m: 0.39 ± 0.92	i‐PRF group showed significantly greater VAS improvement
Torul et al. (2021)^192^; Parallel retrospective	54, 2/52, Control: 35.44 ± 16.83; Test 1: 28.33 ± 11.95; Test 2: 32.61 ± 12.89	NR	Arthrocentesis (*n* = 18); Arthrocentesis + HA (*n* = 18); Arthrocentesis + i‐PRF (*n* = 18)	700 rpm (60 × *g*); 3 min; Duo centrifuge	Pain (VAS at rest and function; 0 d, 1 w and 1 and 3 m)	VAS rest = *Arthrocentesis*: 0 d: 6.50 ± 2.43; 1 w: 4.56 ± 2.44; 1 m: 4.50 ± 1.97; 3 m: 4.50 ± 2.0; *Arthrocentesis + HA*: 0 d: 6.06 ± 2.58; 1 w: 3.72 ± 1.87; 1 m: 3.61 ± 1.78; 3 m: 3.44 ± 1.91; *Arthrocentesis* + *i‐PRF*: 0 d: 5.94 ± 2.10; 1 w: 2.89 ± 1.64; 1 m: 1.83 ± 0.78; 3 m: 1.44 ± 0.92; VAS functio*n* = *Arthrocentesis*: 0 d: 6.50 ± 2.60; 1 w: 4.39 ± 2.38; 1 m: 4.78 ± 1.89; 3 m: 4.89 ± 1.87; *Arthrocentesis + HA*: 0 d: 7.06 ± 2.50; 1 w: 4.11 ± 1.96; 1 m: 4.17 ± 1.88; 3 m: 4.22 ± 1.80; *Arthrocentesis* + *i‐PRF*: 0 d: 5.89 ± 2.11; 1 w: 2.67 ± 1.74; 1 m: 1.78 ± 0.87; 3 m: 1.56 ± 0.92	i‐PRF more effective than other groups
Abdelrahman et al. (2023)^193^; Parallel RCT	30, 4/26, NR	PCT (500 mg; b.i.d.; 2 d)	Dextrose (*n* = 15); i‐PRF (*n* = 15)	700 rpm; 3 min; NR	Pain (VAS; Day 0 d, 1 w and 1, 3 and 6 m)	*Dextrose*: 0 d: 8.47 ± 0.64; 1 w: 5.27 ± 1.03; 1 m: 3.60 ± 1.12; 3 m: 2.53 ± 1.06; 6 m: 3.07 ± 1.16; i*‐PRF*: 0 d: 8.20 ± 0.94; 1 w: 2.80 ± 1.32; 1 m: 2.40 ± 1.24; 3 m: 1.87 ± 0.52; 6 m: 1.73 ± 0.96	i‐PRF had significantly lower pain at all timepoints
Işık et al. (2023)^194^; Parallel RCT	76, 7/69, Control: 46.8 ± 10.2; Test: 47.2 ± 9.1	PCT (500 mg; 7 d)	Arthrocentesis (*n* = 38); Arthrocentesis + i‐PRF (*n* = 38)	700 rpm; 3 min; NR	Pain (VAS; 0 d and 1, 2, 3, 6 and 12)	Pain on palpation: *Arthrocentesis*: 0 d: 7.9 ± 1.4; 1 m: 2.9 ± 0.7; 2 m: 2.5 ± 0.7; 3 m: 2.3 ± 0.7; 6 m: 2.0 ± 0.7; 12 m: 2.8 ± 0.7; *Arthrocentesis + i‐PRF*: 0 d: 7.8 ± 1.5; 1 m: 1.7 ± 0.8; 2 m: 1.4 ± 0.7; 3 m: 1.2 ± 0.7; 6 m: 1.0 ± 0.6; 12 m: 0.9 ± 0.6	i‐PRF significantly improved pain and mobility
Sharma et al. (2023)^195^; Parallel RCT	14, NR, NR	NR	Arthrocentesis + PRP (*n* = 7); Arthrocentesis + i‐PRF (*n* = 7)	700 rpm; 3 min; NR	Pain (VAS; 0 d and 1, 2, 3, 4, 5, 6 and 9 m)	*PRP*: 0 d: 7.11 ± 1.10; 1 m: 4.86 ± 1.80; 2 m: 3.54 ± 2.32; *i‐PRF*: 0 d: 7.07 ± 1.48; 1 m: 6.93 ± 1.81; 2 m: 6.18 ± 2.33	i‐PRF outperformed PRP for pain and function
Vingender et al. (2023)^196^; Parallel prospective	68, 9/59, 53 ± 16	NR	Arthrocentesis + HA (*n* = 28); Arthrocentesis + i‐PRP (*n* = 21); Arthrocentesis + i‐PRF (*n* = 19)	NR	Pain (VAS; 0 d and 6 and 12 m)	*HA*: 0 d: 6.6; 6 m: 1.8; 12 m: 1; *PRP*: 0 d: 5; 6 m: 1.2; 12 m: 0.7; *PRF*: 0 d: 5; 6 m: 2.3; 12 m: 1.1	All groups improved; platelet concentrates preferred
Akkaş and Esen (2024)^197^; Parallel retrospective	116, 13/103, Control: 32.77 ± 14.43; Test 1: 28.91 ± 12.89; Test 2: 36.5 ± 15.95	IBU (600 mg; b.i.d.); Tizanidine (6 mg; OD; 7 d)	Arthrocentesis (*n* = 35); Arthrocentesis + i‐PRF (*n* = 47); Arthrocentesis + i‐PRP (*n* = 34)	700 rpm; 3 min; NR	Pain (VAS; 0 d, 1 w and 1, 3 and 6 m)	*Arthrocentesis*: 0 d: 55.37 + 21.05; 1 w: 28.26 + 24.23; 1 m: 21.40 + 23.34; 3 m: 19.91 + 25.42; 6 m: 19.91 + 25.93; *Arthrocentesis + i‐PRP*: 0 d: 52.88 + 28.02; 1 w: 9.71 + 19.54; 1 m: 20.44 + 18.70; 3 m: 18.09 + 17.87; 6 m: 17.91 + 19.54; *Arthrocentesis* + *i‐PRF*: 0 d: 48.77 + 26.93; 1 w: 28.40 + 20.62; 1 m: 19.68 + 19.61; 3 m: 16.60 + 21.75; 6 m: 11.72 + 15.62	No significant intergroup pain difference
Nasef et al. (2024)^198^; Parallel RCT	30, NR, NR	NSAIDs, an anti‐edematous drug, and a muscle relaxant	ARS + arthrocentesis (*n* = 15); ARS + arthrocentesis + i‐PRF (*n* = 15)	700 rpm; 3 min; NR	Pain (VAS; 0 d, 1 w and 1, 3 and 6 m)	*ARS + arthrocentesis*: 0 d: 6.8 ± 1.3; 1 w: 5.5 ± 1.2; 1 m: 3.9 ± 1.0; 3 m: 2.5 ± 0.9; 6 m: 1.5 ± 1.1; *ARS + arthrocentesis + i‐PRF*: 0 d: 7.3 ± 1.3; 1 w: 5.2 ± 1.3; 1 m: 3.3 ± 1.0; m: 1.6 ± 0.6; 6 m: 0.5 ± 0.6	Significant pain reduction favoring i‐PRF
Tepecik and Baş (2024)^199^; Parallel RCT	88, 88, Control: 36.8 ± 10.2; Test 1: 34.8 ± 8.9; Test 2: 37.6 ± 11.6	AMOX/clav (500/125 mg); Dexketoprofen trometamol (25 mg)	Arthrocentesis (*n* = 30); Arthrocentesis + i‐HA (*n* = 29); Arthrocentesis + i‐PRF (*n* = 29)	700 rpm; 3 min; NR	Pain (VAS; 0 d and 1 and 6 m)	*Arthrocentesis*: 0 d: 63.2 ± 8.7; 1 m: 36.5 ± 10.8; 6 m: 34.8 ± 16.3; *Arthrocentesis + i‐HA*: 0 d: 66.7 ± 9.6; 1 m: 29.0 ± 11.5; 6 m: 24.7 ± 12.7; *Arthrocentesis* + *i‐PRF*: 0 d: 66.2 ± 9.6; 1 m: 35.9 ± 9.8; 6 m: 25.3 ± 13.4	HA and i‐PRF both superior to control
Chaulagain et al. (2025)^200^; Parallel RCT	48, 12/36, Control: 29.9 ± 7.8; Test 1: 36.5 ± 10.9; Test 2: 27.2 ± 8.5	Analgesics (3 d)	Arthrocentesis (*n* = 16); i‐PRF (*n* = 16); Arthrocentesis + i‐PRF (*n* = 16)	700 rpm; 3 min; NR	Pain (VAS); Masticatory muscle tenderness; 0 and 10 d, 1 and 3 m; QoL; 0 d and 3 m	*Arthrocentesis*: 0 d: 6.7; 10 d: 5.9; 1 m: 4.1; 3 m: 3.6; *i‐PRF*: 0 d: 7.2; 10 d: 4.4; 1 m: 3.3; 3 m: 2.9; *Arthrocentesis + i‐PRF*: 0 d: 7.4; 10 d: 5.6; 1 m: 3.4; 3 m: 1.4	AC + i‐PRF had best outcomes across all parameters
Bera and Tiwari (2021)^202^; Parallel retrospective	130, NR, Control: 34.3731 ± 11.5; Test: 32.381 ± 8.9	Analgesics	Arthrocentesis (*n* = 67); Arthrocentesis + i‐PRF (*n* = 63)	700 rpm; 3 min; NR	Pain (VAS; 0 and 15 d, and 1, 3 and 6 m)	*Arthrocentesis*: 0 d: 8.31 ± 0.94; 15 d: 7.69 ± 1.32; 1 m: 5.4 ± 0.63; 3 m: 3.54 ± 0.89; 6 m: 0.27 ± 0.45; *Arthrocentesis + i‐PRF*: 0 d: 8.37 ± 0.94; 15 d: 7.37 ± 0.94; 1 m: 6.37 ± 0.94; 3 m: 3.27 ± 0.45; 6 m: 0.27 ± 0.45	No significant pain difference at 6 m
Işık et al. (2022)^203^; Parallel RCT	36, 3/33, Control: 45.72 ± 13.12; Test: 44.67 ± 12.13	PCT (7 d)	Arthrocentesis (*n* = 18); Arthrocentesis + i‐PRF (*n* = 18)	700 rpm; 3 min; NR	Pain (VAS; 0 d and 1, 2, 3, 6, and 12 m)	Pain on palpation: *Arthrocentesis*: 0 d: 8.06 ± 1.16 1 m: 4.00 ± 0.76 2 m: 3.39 ± 1.83 3 m: 3.28 ± 0.75 6 m: 2.78 ± 0.64 12 m: 3.39 ± 0.69 *Arthrocentesis + i‐PRF*: 0 d: 7.83 ± 1.20 1 m: 3.22 ± 0.80 2 m: 2.11 ± 0.83 3 m: 1.67 ± 0.76 6 m: 1.67 ± 0.68 12 m: 1.72 ± 0.75	i‐PRF showed significantly better pain relief
Tepecik and Gedik (2025)^204^; Parallel Retrospective	127, 0/127, 52.3 ± 9.8	AMOX/clav (500/125 mg); Dexketoprofen trometamol (25 mg)	Arthrocentesis (*n* = 45); Arthrocentesis + i‐PRF (*n* = 43); Arthrocentesis + HA (*n* = 39)	700 rpm (60 × *g*); 3 min; NR	Pain (VAS; 0 d and 1, 6, and 12 m)	*Arthrocentesis*: 0 d: 0.7 ± 9.3; 1 m: 38.8 ± 9.8; 6 m: 34.7 ± 12.7; 12 m: 35.9 ± 16.1; *Arthrocentesis + i‐HA*: 0 d: 69.5 ± 9.0; 1 m: 29.0 ± 6.8; 6 m: 25.6 ± 8.4; 12 m: 19.9 ± 14.4; *Arthrocentesis + i‐PRF*: 0 d: 71.3 ± 10.0; 1 m: 32.4 ± 0.0; 6 m: 24.5 ± 10.3; 12 m: 20.6 ± 16.4	No significant difference in pain between i‐PRF and HA
TMJ hypermobility							
Baiomy et al. (2019)^205^; Parallel RCT	30, NR, NR	NR	Autologous blood (*n* = 15); i‐PRF (*n* = 15)	700 rpm (60 × *g*); 3 min; NR	Pain (VAS); 0 d, 1, and 3 m	*Autologous blood*: 0 d: – 8.8 ± 0.91; 1 m: – 6.5 ± 1.67; 3 m: – 2.7 ± 1.33; *i‐PRF*: 0 d – 8.9 ± 1.11; 1 m: – 4.63 ± 0.55; 3 m: – 1.1 ± 0.95	i‐PRF showed significantly better pain control
Unspecified TMJ pain							
Kumar et al. (2024)^206^; Parallel RCT	68, 17/51, Control: 42.5 ± 8.2; Test: 41.8 ± 7.5	NR	No treatment (*n* = 34); i‐PRF (*n* = 34)	700 rpm; 3 min; Hettich® EBA 20 centrifuge	Pain (VAS); QoL and satisfaction (PROs); 0 d, 6, 12 and 24 w	*No treatment*: 0 d: 6.0 ± 1.4; 6 w: 5.8 ± 1.3; 12 w: 5.6 ± 1.2; 24 w: 5.4 ± 1.1; *i‐PRF*: 0 d: 6.2 ± 1.5; 6 w: 3.2 ± 1.2; 12 w: 2.5 ± 1.0; 24 w: 2.0 ± 0.8	I‐PRF reduced pain, improved jaw function and PROs

Abbreviations: AC, arthrocentesis; AMOX, amoxicillin; ARS, anterior repositioning splint; AUG, augmentin; b.i.d., bis in die, twice a day; Clav, clavulanic acid; d, day; HA, hyaluronic acid; IBU, Ibuprofen; i‐PRF, injectable platelet‐rich fibrin; i‐PRP, injectable platelet‐rich plasma; m, month; min, minutes; NR, not reported; NSAIDs, nonsteroidal anti‐inflammatory drugs; OD, once daily; PCT, paracetamol; PRF, platelet‐rich fibrin; PROs, patient‐reported outcomes; PRP, platelet‐rich plasma; QoL, quality of life; RCT, randomized clinical trial; rpm, revolution per minute; t.i.d., ter in die, three times a day; TMJ, temporomandibular joint; VAS, visual analog scale; w, week.

Pain outcomes favored PRF in 14 of the included studies, with significantly reduced pain scores reported in the PRF group compared with control or non‐PRF‐treated groups,^189^, ^190^, ^191^, ^192^, ^193^, ^194^, ^195^, ^198^, ^199^, ^200^, ^201^, ^203^, ^205^, ^206^ In contrast, four studies demonstrated no statistically significant difference in pain between groups.^196^, ^197^, ^202^, ^204^

Two studies assessed the effect of PRF on QoL. Chaulagain et al.^200^ reported a significant improvement in QoL when i‐PRF was used in conjunction with arthrocentesis compared with i‐PRF alone or arthrocentesis alone. Kumar et al.^206^ demonstrated improved QoL in the i‐PRF group versus the no‐treatment control group for nonspecific TMJ pain. In total, 11 studies reported prescribing postoperative analgesics.^189^, ^193^, ^194^, ^197^, ^198^, ^199^, ^200^, ^201^, ^202^, ^203^, ^204^

### 3.14 Orthopedics

A total of five studies were included that utilized PRF in orthopedic procedures, including rotator cuff repair,^207^ arthrodesis,^208^ sacroiliac joint dysfunction,^209^ posterior lumbar interbody fusion,^210^ and tendon‐exposed wound healing (Table 14).^211^ Of these, three studies were RCTs,^207^, ^209^, ^211^ while two were retrospective analyses.^208^, ^210^ All five studies employed a parallel‐group design.^207^, ^208^, ^209^, ^210^, ^211^

**TABLE 14:** Summary of studies evaluating pain reduction with PRF in orthopedics.

Study; sesign	Sample size, M/F, mean age (years)	Postoperative medications	Control; test groups	PRF preparation (speed and time; manufacturer)	Parameters assessmed	Pain scores	Main findings
Rotator cuff repair							
Zumstein et al. (2016)^207^; Parallel RCT	35, 18/17, Control: 65; Test: 66	NR	Arthroscopic rotator cuff repair (*n* = 18); Arthroscopic rotator cuff repair + L‐PRF (*n* = 17)	NR; NR; NR	Pain (VAS; 0 d and 3, 6 and 12 m)	*Arthroscopic rotator cuff repair*: 0 d: 6.0; 12 m: 13.6; *Arthroscopic rotator cuff repair + L‐PRF*: 0 d: 6.3; 12 m: 13.9	No significant difference in pain at any timepoint
Arthrodesis							
Bernasconi et al. (2019)^208^; Parallel retrospective	34, 2/32, Control: 70.5; Test: 67	NR	MTPJ1 arthrodesis (*n* = 20); MTPJ1 arthrodesis + A‐PRF (*n* = 14)	3000 rpm; 10 min; NR	Pain (VAS); AOFAS; SEFASl EQ‐5 d; Final follow‐up	*MTPJ1 arthrodesis*: Final follow‐up: 2 (0–3) *MTPJ1 arthrodesis + A‐PRF*: Final follow‐up: 0 (0–1)	AOFAS, VAS and EQ‐5 d similar; SEFAS favored A‐PRF group. No long‐term difference overall
Sacreoiliac joint dysfunction							
Mohi Eldin et al. (2019)^209^; Parallel RCT	186, NR, Test 1: 46; Test 2: 48	NR	SIJ dysfunction + i‐PRP (*n* = 62); SIJ dysfunction + i‐PRF (*n* = 124)	3300 rpm; 2 min; Horizontal centrifuge	Pain (VAS; 0 d and 1 and 6 m)	*PRP*: 0 m: 8.29 + 0.458; 1 m: 5.47 + 1.696; 6 m: 5.19 + 1.491; *PRF*: 0 d: 8.28 + 0.452; 1 m: 5.06 + 1.869; 6 m: 4.61 + 2.003	SIJP + i‐PRF group showed significantly greater pain improvement than PRP
Posterior lumbar interbody fusion							
Boktor et al. (2020)^210^; Parallel retrospective	40, 11/29, Control: 43.9 ± 9.01; Test: 40.75 ± 7.8	NR	PLIF (*n* = 20); PLIF + PRF (*n* = 20)	2700 rpm; 12 min; NR	Clinical outcome (ODI); Pain (VAS; 0 d and 3, 6, and 12 m)	Back Pain: *PLIF*: 0 d: 6.5 ± 0.7; 3 m: 4.5 ± 1; 6 m: 3.6 ± 1.7; 12 m: 2.4 ± 1.3; *PLIF + PRF*: 0 d: 6.8 ± 0.8; 3 m: 4.2 ± 1.2; 6 m: 2.6 ± 1.9; 12 m: 1.7 ± 1.5; Leg Pain: *PLIF*: 0 d: 5.9 ± 1.2; 3 m: 2.4 ± 1; 6 m: 1 ± 1; 12 m: 0.45 ± 0.7; *PLIF + PRF*: 0 d: 6.4 ± 1.1; 3 m: 2.5 ± 0.99; 6 m: 0.85 ± 1.1; 12 m: 0.5 ± 1	Greater reduction in back pain VAS and better ODI improvement in PRF group. Leg pain VAS was similar

Abbreviations: AOFAS; American Orthopaedic Foot and Ankle Society forefoot scale; b.i.d., bis in die, twice a day; d, day; EQ‐5 d, EuroQol five dimensions questionnaire; i‐PRF, injectable platelet‐rich fibrin; L‐PRF, leukocyte‐and platelet‐rich fibrin; m, month; min, minute; MTPJ1, first metatarsophalangeal; NR, not reported; ODI, Oswestry Disability Index; PLIF, posterior lumbar interbody fusion; PRF, platelet‐rich fibrin; PRP, platelet‐rich plasma; RCT, randomized clinical trial; rpm, revolution per minute; SEFAS, self‐reported foot and ankle score; SIJ, sacroiliac joint dysfunction; VAS, visual analog scale.

Three of the five studies demonstrated significantly reduced postoperative pain in the PRF‐treated groups compared with their respective non‐PRF controls,^209^, ^210^, ^211^ while two studies reported no statistically significant differences in pain outcomes between the PRF and control groups.^207^, ^208^

### 3.15 Facial surgery and aesthetics

A total of four studies examined the application of PRF in facial aesthetics and surgical interventions, including cleft lip and palate repair,^212^, ^213^ facial lipostructure surgery,^214^ and microneedling procedures (Table 15).^215^ All four studies were RCTs,^212^, ^213^, ^214^, ^215^ with two studies using a parallel design,^212^, ^213^ and the remaining two utilized a split‐mouth or split‐face design.^214^, ^215^

**TABLE 15:** Summary of studies included evaluating pain reduction with PRF in facial surgery and aesthetics.

Study; design	Sample size, M/F, mean age (years)	Postoperative medications	Control; Test Groups	PRF preparation (speed and time; manufacturer)	Parameters assessed	Pain scores	Main findings
Cleft Lip/Palate							
El‐Ahmady et al. (2018)^212^; Parallel RCT	20, 8/12, 11.5	Antibiotics, analgesics and anti‐inflammatory medications and an antiseptic mouthwash	Autogenous iliac crest bone (*n* = 10); BMMNCs + collagen sponge + nanohydroxyapatite + PRF (*n* = 10)	3000 rpm; 20 min; NR	Pain (NRS); swelling; satisfaction; 1 d, 1 and 3 w and 6 and 12 m	*Pain was notably* minimal in all patients. On day one post‐op, it was described by all patients as a seven on the pain scale. It was then dropped to five on day three and then to two after 1 week	PRF group had less pain and fewer complications
Bedeer et al. (2024)^213^; Parallel RCT	36, 18/18, Control: 12.13 ± 3.07; Test: 10.56 ± 1.96	NSAIDS (5–7 d)	Autogenous iliac crest (*n* = 18); Xenograft + PRF (*n* = 18)	3000 rpm; 10 min; NR	Pain (VAS; 2 and 14 d, and 6 m; hospital stay; 2–4 d)	All patients at donor site experienced some pain.	PRF + Xenograft group reported less pain and shorter hospital day
Facial lipostructure surgery							
Keyhan et al. (2013)^214^; Split‐mouth RCT	25, 8/17, 45	NR	Fat Graft + PRP (*n* = 25); Fat graft + PRF (*n* = 25)	3000 rpm; 10 min; NR	Pain; swelling; 1 m and 1 y	NR	Both groups had mild pain and edema; no severe cases
Microneedling							
Fayyaz et al. (2025)^215^; Split‐face RCT	50, 29/21, 27.68 ± 4.93	NR	PRP (*n* = 50); PRF (*n* = 50)	700 rpm; 3 min; NR	Pain (VAS); swelling; satisfaction	PRP: 2.54 ± 1.27 PRF: 2.96 ± 1.29	No significant difference in pain or swelling; PRF group had higher satisfaction

Abbreviations: BMMNC, bone marrow mononuclear cells; d, day; m, month; min, minutes; NR, not reported; NRS, numeric rating scale; NSAIDs, nonsteroidal anti‐inflammatory drugs; PRF, platelet‐rich fibrin; PRP, platelet‐rich plasma; RCT, randomized clinical trial; rpm, revolution per minute; VAS, visual analog scale; w, week.

Two studies reported a statistically significant reduction in postoperative pain in the PRF group compared with the non‐PRF control group.^212^, ^213^ The remaining two studies reported no significant difference in pain levels between PRF and control groups^214^, ^215^; however, other patient‐centered outcomes were explored. Keyhan et al.^214^ compared PRF and PRP in combination with autologous fat grafts during facial lipostructure surgery and reported no cases of postoperative pain or edema in either group. Similarly, Fayyaz et al.^215^ compared PRF with PRP in facial microneedling and reported no significant differences in pain perception; however, the PRF group exhibited higher levels of patient satisfaction, potentially reflecting better overall healing or aesthetic outcomes. Two of the included studies prescribed analgesics postoperatively.^212^, ^213^

### 3.16 Miscellaneous medical procedures

A total of eight studies were included that examined the analgesic effects of PRF in additional medical procedures outside of dentistry (Table 16). These comprised three studies focused on chronic wound care,^216^, ^217^, ^218^ three studies involving otorhinolaryngologic (ENT) interventions, and^219^, ^220^, ^221^ two studies in ophthalmologic procedures.^222^, ^223^ Among these, six studies were RCTs,^216^, ^217^, ^218^, ^219^, ^221^, ^223^ one was a prospective clinical study,^220^ and one was retrospective in design.^222^ All eight studies utilized a parallel‐group design.

**TABLE 16:** Summary of studies included evaluating pain reduction with PRF in miscellaneous medical procedures.

Study; design	Sample size, M/F, mean age (years)	Postoperative medications	Control; test groups	PRF Preparation (speed and time; manufacturer)	Parameters assessed	Pain scores	Main findings
Chronic wound care							
Chignon‐Sicard et al. (2012)^216^; Parallel RCT	64, 54/10, Control: 66 ± 7.7; Test: 61.4 ± 8.8	NR	Petroleum jelly mesh (*n* = 31); L‐PRF (*n* = 33)	2700 rpm (400 × g); 12 min; NR	Pain (VAS; 1, 2, 7, 14, 21, 28 and 60 d)	NR	Lower pain in L‐PRF group, not statistically significant
Vaheb et al. (2021)^217^; Parallel RCT	33, 17/16, 33.10 ± 2.60	NR	Vaseline petrolatum gauze and wet dressing (*n* = 33); Vaseline petrolatum gauze and wet dressing + PRF gel (*n* = 33)	3000 rpm; 10 min; Thermo Scientific Sorvall BP 16	Pain (VAS; 0, 8 and 15 d)	*Vaseline petrolatum gauze* and *wet dressing*: 0 d: 8.50 ± 0.50; 8 d: 5.6 ± 0.50; 15 d: 3.5 ± 0.40; *Vaseline petrolatum gauze* and *wet dressing + PRF gel*: 0 d: 8.70 ± 0.70; 8d: 3.4 ± 0.40; 15 d: 2.8 ± 0.30	PRF significantly reduced donor site pain
Lin et al. (2023)^218^; Parallel RCT	120, 72/48, Control: 29.89 ± 3.89; Test: 30.35 ± 4.15	NR	AgNP dressing (*n* = 60); AgNP + PRF dressing (*n* = 60)	3000 rpm; 10 min; NR	Pain (VAS; 1, 3 and 5 d)	*AgNP dressing*: 1 d: 5.56 ± 0.62; 3 d: 3.42 ± 1.12; 5 d: 3.79 ± 0.70; *AgNP dressing + PRF*: 1 d: 5.47 ± 0.55; 3 d: 4.59 ± 0.93; 5 d: 2.63 ± 0.52	PRF + AgNP dressing alleviated pain more effectively
Otorhinolaryngology procedures							
Elkahwagi et al. (2019)^219^; Parallel RCT	30, NR, 38.4 ± 7.85	NR	Relocation pharyngoplasty (*n* = 15); Relocation pharyngoplasty + PRF (*n* = 15)	3000 rpm; 10 min; Model 800 centrifuge	Pain (VAS; 1, 3, 5, and 10 d)	*Relocation pharyngoplasty*: 1 d: 9; 3 d: 8; 5 d: 5; 10 d: 3; *Relocation pharyngoplasty + PRF*: 1 d: 8; 3 d: 6; 5 d: 3; 10 d: 1	PRF group had significantly less pain, especially on 5 and 10 d
Tutar et al. (2020)^220^; Parallel Prospective	141, NR, NR	AMOX/clav (1 g; b.i.d.); nasal irrigation (5 d)	Septoplasty (*n* = 67); Septoplasty + PRF (*n* = 74)	3000 rpm; 15 min; NR	Pain (VAS; 1 and 3 w)	*Septoplasty*: 1 w: 6.37 ± 2.269; 3 w: 2.37 ± 1.369; *Septoplasty + PRF*: 1 w: 4.10 ± 2.091; 3 w: 0.49 ± 0.940	PRF reduced pain in the early postoperative period
Reksodiputro et al. (2021)^221^; Parallel RCT	20, 20, 60	NR	Total laryngectomy (*n* = 10); Total Laryngectomy + PRF (*n* = 10)	3000 rpm (±400 × *g*); 10 min; Regenlab PRP‐Centri Type RGL‐PRP‐C	Pain (VAS; 2, 3, 4, 5, 6, and 14 d)	*Total Laryngectomy*: 2 d: 3 patients mild pain, 7 patients moderate pain; 3 d: 2 patients mild pain, 7 patients moderate pain, one patient severe pain; 4 d: two patients mild pain, six patients moderate pain, two patients severe pain; 5 d: two patients mild pain, one patients moderate pain, one patient severe pain; 6 d: three patients mild pain, seven patients moderate pain; 14 d: 0 patients mild pain, four patients moderate pain, six patients severe pain. *Total Laryngectomy + PRF*: 2 d: nine patients mild pain, 0 patients moderate pain, one patient severe pain; 3 d: six patients mild pain, three patients moderate pain, one patient severe pain; 4 d: six patients mild pain, three patients moderate pain, one patient severe pain; 5 d: nine patients mild pain, one patient moderate pain; 6 d: nine patients mild pain, one patient moderate pain; 14 d: two patients mild pain, five patients moderate pain, three patients severe pain	PRF enhanced wound healing and reduced pain
Ophthalmology procedures							
Bahar and Sabur (2024)^222^; Parallel Retrospective	65, 37/28, Control: 53.6 ± 8.4; Test: 54.8 ± 8.1	Pressure patch with antibiotic ointment for 1 d; 0.3% netilmicin + 0.1% DEX eye drops QID for 1 m	Conjunctival autograft (*n* = 34); Conjunctival autograft + i‐PRF (*n* = 31)	700 rpm; 3 min; DLAB 0506	Pain; 1 d	*Surgery*: 1 d: 5.08 ± 1.40; *Surgery + PRF*: 1 d: 4.45 ± 1.52	No significant differences in postoperative pain
Bhattacharjee et al. (2025)^223^; Parallel RCT	25, 16/9, 38.8 ± 8.8	NR	Dermis‐fat graft with fornix forming sutures (*n* = 14); Dermis‐fat graft with fornix forming sutures + PRF (*n* = 11)	3000 rpm; 12 min; NR	Pain (VAS; 1 d and 1 w)	Among the PRF group, 72% of patients showed statistically significantly lower pain intensity in VAS as compared with the non‐PRF group on postoperative 1 d	PRF group had significantly less pain on 1 d

Abbreviations: AgNP: nano silver; AMOX: amoxicillin; Clav: clavulanic acid; d: day; DEX: dexamethasone; i‐PRF: injectable platelet‐rich fibrin; L‐PRF: leukocyte‐and platelet‐rich fibrin; min: minutes; NR: not reported; PRF: platelet‐rich fibrin; QID: quater in die, four times a day; RCT: randomized clinical trial; rpm: revolution per minute; VAS: visual analog scale; w: week.

Six studies reported significantly reduced pain levels in the PRF group compared with the non‐PRF group.^217^, ^218^, ^219^, ^220^, ^221^, ^223^ Two studies, however, reported no significant difference in pain outcomes between PRF and non‐PRF groups.^216^, ^222^ Only one study explicitly reported postoperative analgesic prescription.^222^

### 3.17 Risk of bias outcomes

The RoB of the included studies is shown in the Figures S1–S32. For RCTs, a total of 22 studies were categorized as “low” RoB, 132 as “some,” and 15 as “serious” RoB. For non‐RCTs, a total of two studies were classified as “low,” 11 as “moderate,” and 18 as “serious” RoB. For the RoB of the individual studies, please refer to Data S1.

## 4 DISCUSSION

This systematic review represents the first comprehensive synthesis of clinical studies evaluating the analgesic effects of PRF across both medical and dental disciplines. A total of 200 comparative clinical studies were included, offering a broad evidence base across several procedural settings. Among these, 65.5% of studies reported reduced pain in the PRF group compared with non‐PRF controls, 31.0% of studies found no significant difference, and only 3.5% of studies reported higher pain in the PRF‐treated cohort. These outcomes highlight the substantial analgesic potential of PRF while also underscoring variability across methodological designs, procedural contexts, and outcome measures.

The largest subset of included studies (*n* = 54) focused on third molar extractions, a common model for assessing postoperative pain and healing.^224^ Consistent with the published literature, post‐extraction pain peaks within 5–6 h and may persist for numerous days owing to the release of inflammatory mediators.^225^ Of the studies examining swelling (*n* = 40), 73% of studies reported significantly less swelling in PRF groups, indicating that PRF may also exert anti‐edematous effects. This observation is supported by multiple in vitro studies on solid and liquid PRF,^226^, ^227^, ^228^ suggesting the ability for PRF to reduce swelling by decreasing pro‐inflammatory mediators in macrophages and mesenchymal cells and thereby inhibiting osteoclastogenic inflammatory responses.^229^, ^230^ Some of the pro‐inflammatory mediators that were studied include IL‐1β, IL‐6, CCL2, and CCL5.^227^, ^229^ Despite the evidence that PRF may reduce inflammatory reactions in vitro, studies are still not clear on the effective component at which PRF mediates these functions.^227^, ^229^ Based on recent studies, the main mechanism of action for PRF application may lie in its antioxidant promotion as it neutralizes the hydrogen peroxide produced in gingival fibroblasts,^231^ and thus its improvement of local immune responses.^229^ Nonetheless, it is imperative to note that swelling was not a primary inclusion criterion, and future systematic reviews focusing particularly on PRF's anti‐edematous and anti‐inflammatory potential are recommended.

One additional finding was that overall, procedures that tend to generate the most patient‐reported pain, such as third molar extractions and autogenous soft tissue grafting from the palate were the most commonly studied (Tables 1, 3, 4). Among the 54 studies investigating third molar extractions, 72.2% reported significantly reduced postoperative pain, and 87.5% reported less analgesic use in the PRF group. Similarly, out of 20 studies evaluating root coverage procedures, gingivectomy/gingivoplasty procedures, vestibuloplasty, or apically positioned flaps, 85% reported significantly reduced pain levels in the PRF group compared with the controls. These findings highlight the fact that for studies generating typically the most patient‐reported pain scores, not only were those procedures studied more commonly than others, but they also generated the best results. It may therefore be concluded that the adjunctive use of PRF may be better be suited for procedures with higher overall patient‐reported pain scores, as these preliminary results seem to support these findings.

The frequent utilization of CHX as a postoperative mouth rinse was reported in 74 (37.0%) of the included studies. While CHX is commonly prescribed, it has been demonstrated in in vitro models to exert cytotoxic and pro‐inflammatory effects, especially on fibroblasts and other regenerative cell types.^232^, ^233^ For example, Fujioka‐Kobayashi et al.^232^ reported a nearly 2000‐fold increase in inflammatory markers when CHX was compared with a natural alternative.^232^ Therefore, it may be speculated that the application of CHX contributed to the pain experienced by patients. The adverse interaction between CHX and PRF, especially its potential to impair platelet function or inhibit growth factor release, raises concerns about confounding pain outcomes in studies utilizing CHX. Our group has also recently published a narrative review on a homeopathic surgical kit with anti‐inflammatory and analgesic properties.^234^ While few studies have demonstrated the reduced pain achieved with this alternative, significantly more data is warranted.^235^, ^236^ Future clinical studies should evaluate CHX‐PRF compatibility and investigate/compare results to nontoxic alternatives for postoperative care.

Beyond conventional PRF, methodological advances have introduced variations including i‐PRF, A‐PRF, and Alb‐PRF, aimed at optimizing cellular content and release kinetics of bioactive molecules.^237^, ^238^, ^239^, ^240^, ^241^, ^242^ Additionally, centrifugation protocols, tube types, and material characteristics (such as hydrophilicity) may significantly affect the biocompatibility and biological activity of PRF preparations.^237^, ^239^, ^243^ Some tubes utilized for PRF fabrication contain chemical additives or silica particles, which have been correlated with pro‐inflammatory responses and may inadvertently increase postoperative pain.^244^, ^245^, ^246^ Therefore, variability observed in certain studies may be attributed to these modifications. Unfortunately, a vast majority of studies published on PRF do not report the types utilized, making comparison difficult. Standardization of PRF preparation, such as centrifugation protocols and tube composition, should be a priority in future research to ensure consistency and maximize therapeutic efficacy.

Several limitations were identified that must be acknowledged and addressed in future research for strengthening the clinical applicability of PRF. A major limitation lies in the significant heterogeneity among the included studies. Heterogeneity in methodological designs (RCTs, prospective and retrospective studies), outcome evaluation methods (follow‐up durations, pain scales), PRF types (L‐PRF, A‐PRF, i‐PRF, Alb‐PRF), and surgical techniques made direct comparisons challenging and precluded the performance of a meta‐analysis. Future studies should adopt standardized PRF preparation methods, such as tube types, centrifugation durations and speeds, to enhance comparability and reproducibility. Additionally, pain evaluation protocols varied significantly across studies, with inconsistent utilization of validated scales and varying postoperative timepoints. The implementation of uniform pain assessment tools and standardized timepoints is necessary to permit meaningful comparisons.

While third molar extractions and mucogingival procedures were well‐represented, other clinical applications, including aesthetic medicine, orthopedics, ophthalmology, ENT, MRONJ, and so forth, remain underexplored. Larger, well‐designed RCTs are required to clarify PRF's role in these domains. Moreover, direct comparisons of varying PRF preparations specifically focused on analgesic outcomes are limited and mandate further research. A significant gap identified was the lack of patient‐reported outcome measures (PROMs), including QoL, satisfaction, and functional recovery. Considering the subjective nature of pain, future investigations should incorporate PROMs utilizing validated tools for providing a more patient‐centered comprehension of PRF's effectiveness.

## 5 CONCLUSION

This systematic review provides compelling evidence that PRF contributes to reduced postoperative pain across a wide range of medical and dental procedures. The autologous nature of PRF, together with the extended release of bioactive factors, most likely plays a vital role in mediating inflammation and improving tissue healing, hence enhancing patient comfort and recovery. As PRF continues to gain traction in medical and dental practice, integrating well‐designed, comparative studies with standardized outcome measures and protocols will be critical to completely comprehend the therapeutic potential and inform evidence‐based guidelines for the application of PRF.

## AUTHOR CONTRIBUTIONS

All authors made substantial contributions to the conception and design of the manuscript. NE, TT, and PA performed the literature search. All other authors contributed significantly through critical analysis of study design, solving disputes regarding certain studies to be excluded/included, and drafting the written manuscript. All authors agree to be accountable for all aspects of the study design and its content, and approved the final submitted version.

## CONFLICT OF INTEREST STATEMENT

Richard J. Miron is the founder of Miron Research and Development in Dentistry LLC, which holds intellectual property on the production of PRF. All other authors declare that they have no competing interests.

## ETHICAL APPROVAL

No ethics approval was required for this study since it was a narrative review.

## INFORMED CONSENT

No informed consent was required.

## Supporting information


Figures S1–S52:

Data S1:

## Data Availability

The data sets used and/or analyzed during the current study are available from the corresponding author upon reasonable request.
