# Supplementary material for: Analgesic effects of platelet‐rich fibrin (PRF): A systematic review
Source: Periodontol 2000. 2025 Oct 13;99(1):132–84. doi: 10.1111/prd.70014 (PMC13428096; doi:10.1111/prd.70014)
Supplement: Supplementary file 1 — Figures S1–S52: [file PRD-99-132-s001.docx]

**Supplementary Material**

**Analgesic Effects of Platelet-rich Fibrin (PRF): A Systematic Review.**

Nathan E. Estrin^1,2,3^, Troy B. Tran^2^, Paras Ahmad^3^, Nima Farshidfar^3,4^, Georgios E. Romanos^5^, Anton Sculean^4^, Richard J. Miron^3,4^

^1^The University of Iowa College of Dentistry and Dental Clinics, Iowa City, Iowa, USA;

^2^Lake Erie College of Osteopathic Medicine School of Dental Medicine, Bradenton, Florida, USA;

^3^Department of Research, Advanced PRF Education, Jupiter, Florida, USA;

^4^Department of Periodontology, University of Bern, Bern, Switzerland;

^5^Department of Periodontics and Endodontics, Laboratory for Periodontal-, Implant-, Phototherapy (La-PIP), School of Dental Medicine, Stony Brook University, Stony Brook, NY, USA.


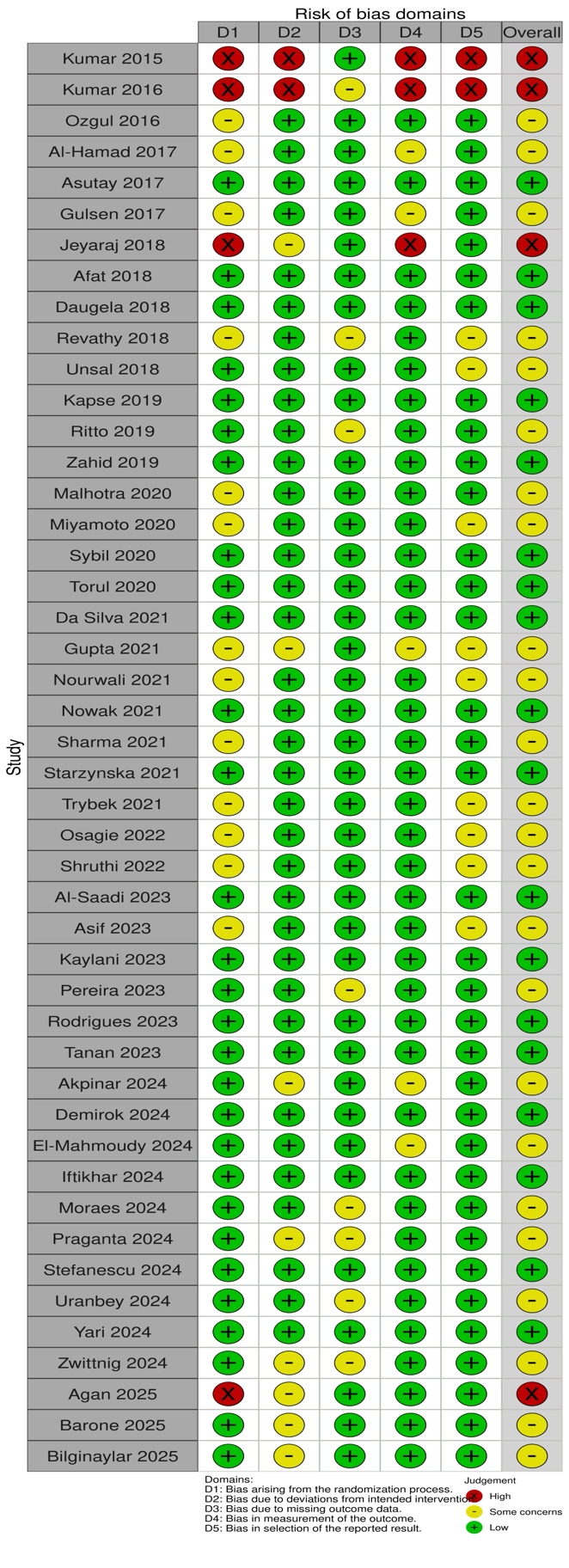


**Figure S1.** Traffic light plot showing ROB-II assessment of the included studies regarding pain reduction with PRF for 3^rd^ molar extractions.


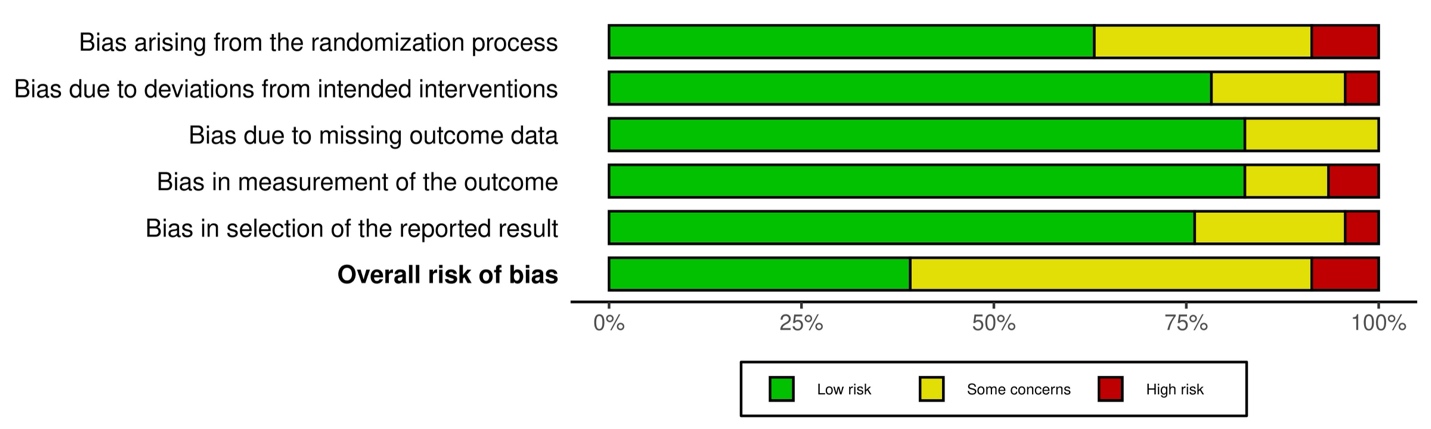


**Figure S2.** Summary plot showing ROB-II assessment of the included studies regarding pain reduction with PRF for 3^rd^ molar extractions.


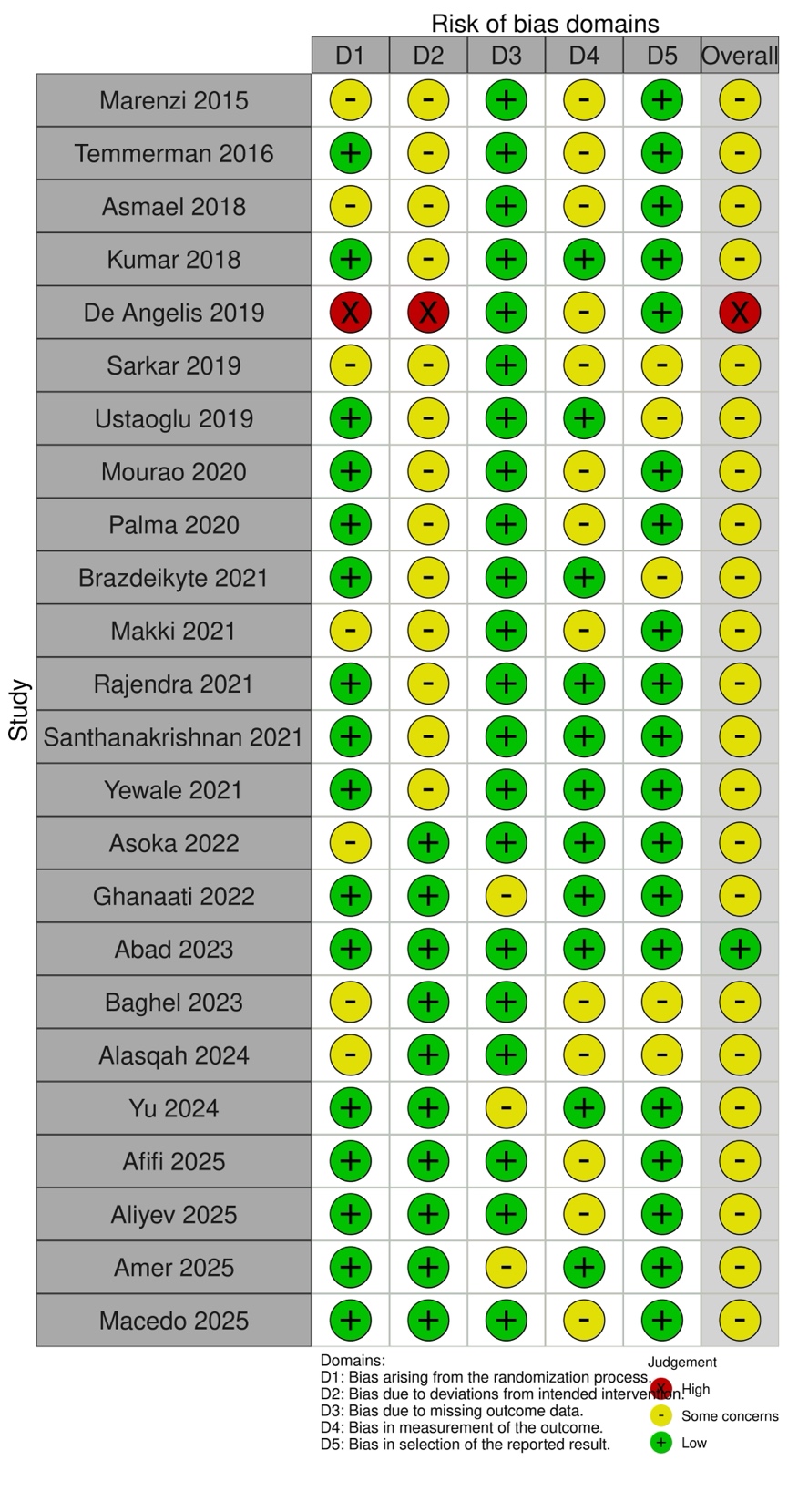


**Figure S3**. Traffic light plot showing ROB-II assessment of the included studies regarding pain reduction with PRF for dental extractions.


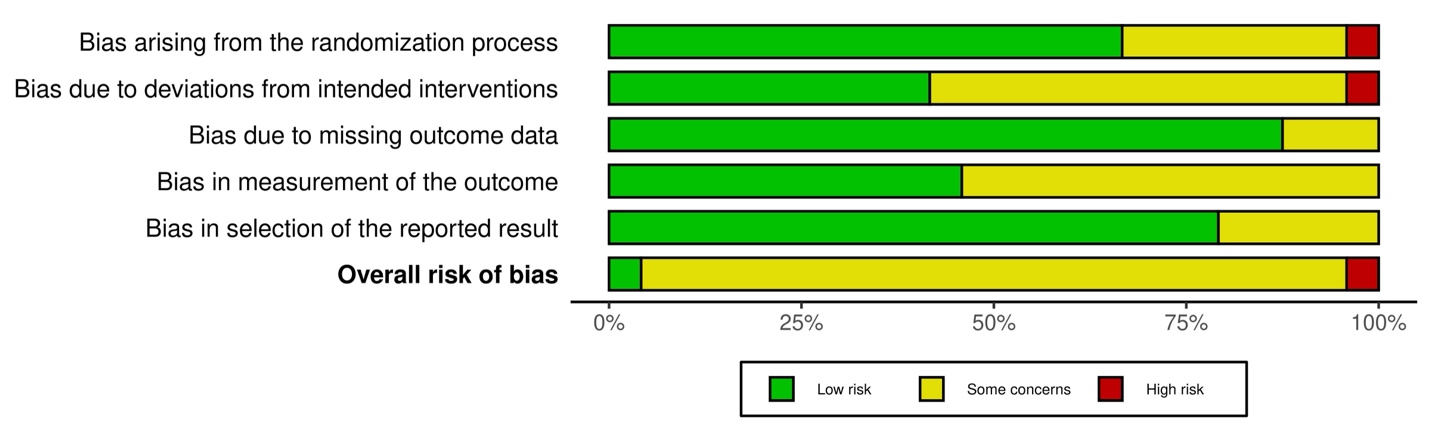


**Figure S4.** Summary plot showing ROB-II assessment of the included studies regarding pain reduction with PRF for dental extractions.


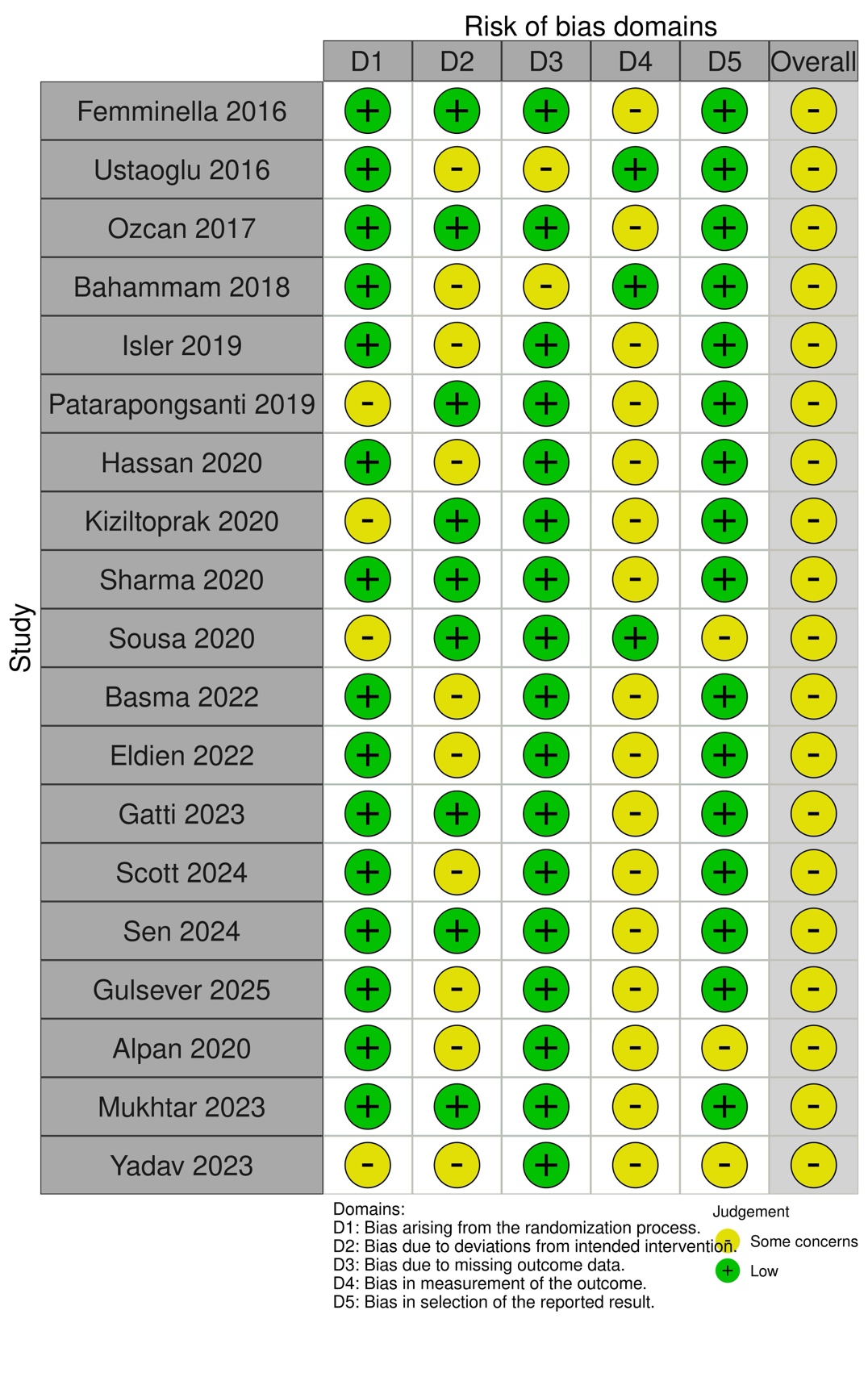


**Figure S5**. Traffic light plot showing ROB-II assessment of the included studies regarding pain reduction with PRF for palatal wound healing.


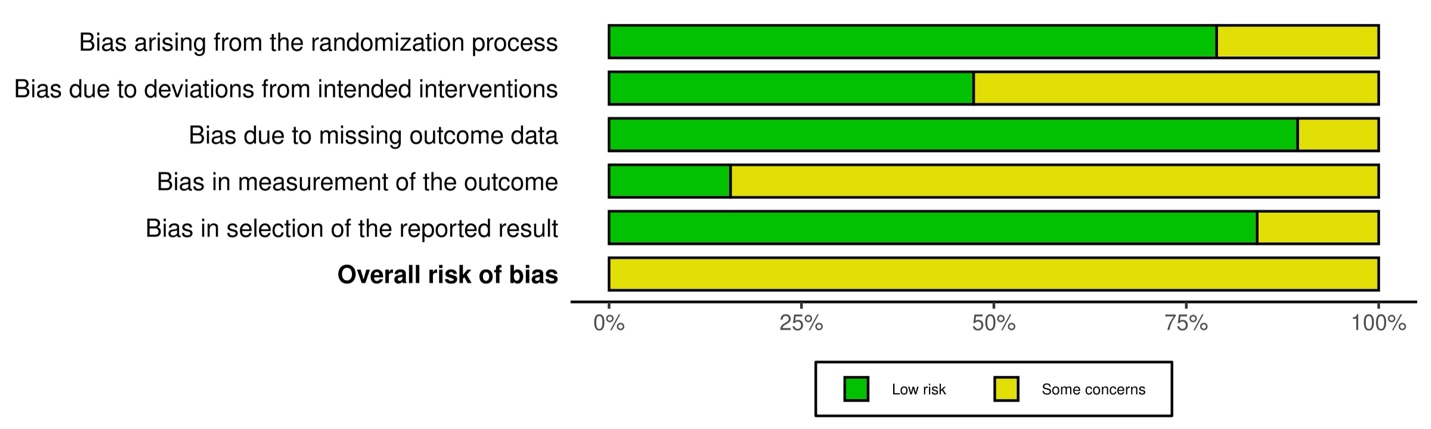


**Figure S6.** Summary plot showing ROB-II assessment of the included studies regarding pain reduction with PRF for palatal wound healing.


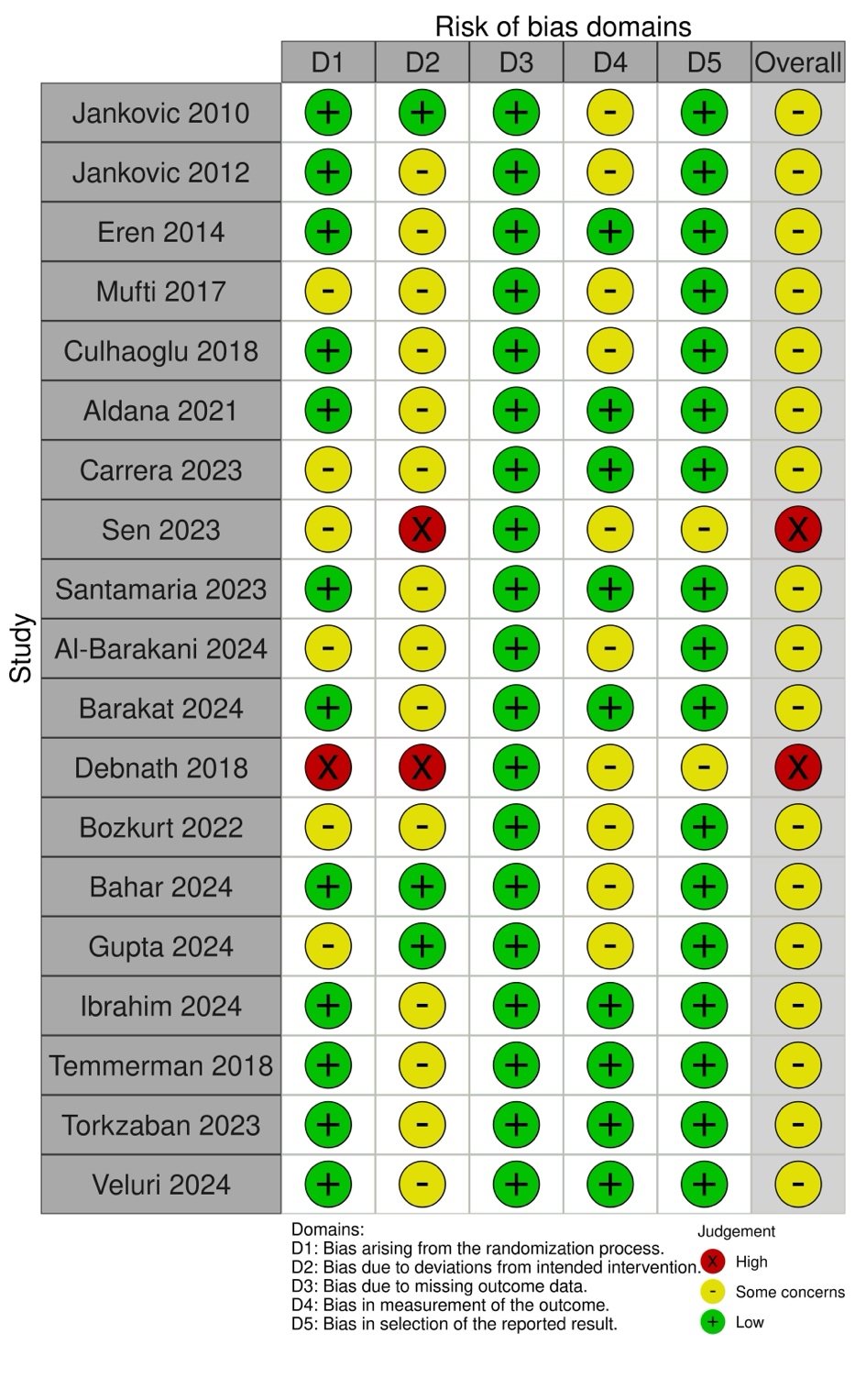


**Figure S7**. Traffic light plot showing ROB-II assessment of the included studies regarding pain reduction with PRF for mucogingival conditions.


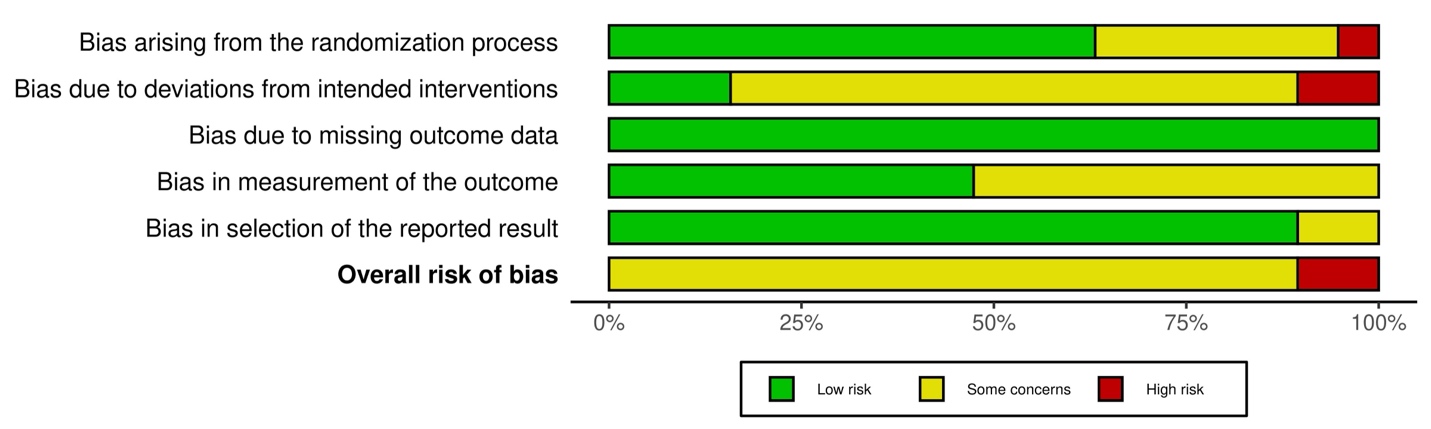


**Figure S8.** Summary plot showing ROB-II assessment of the included studies regarding pain reduction with PRF for mucogingival conditions.


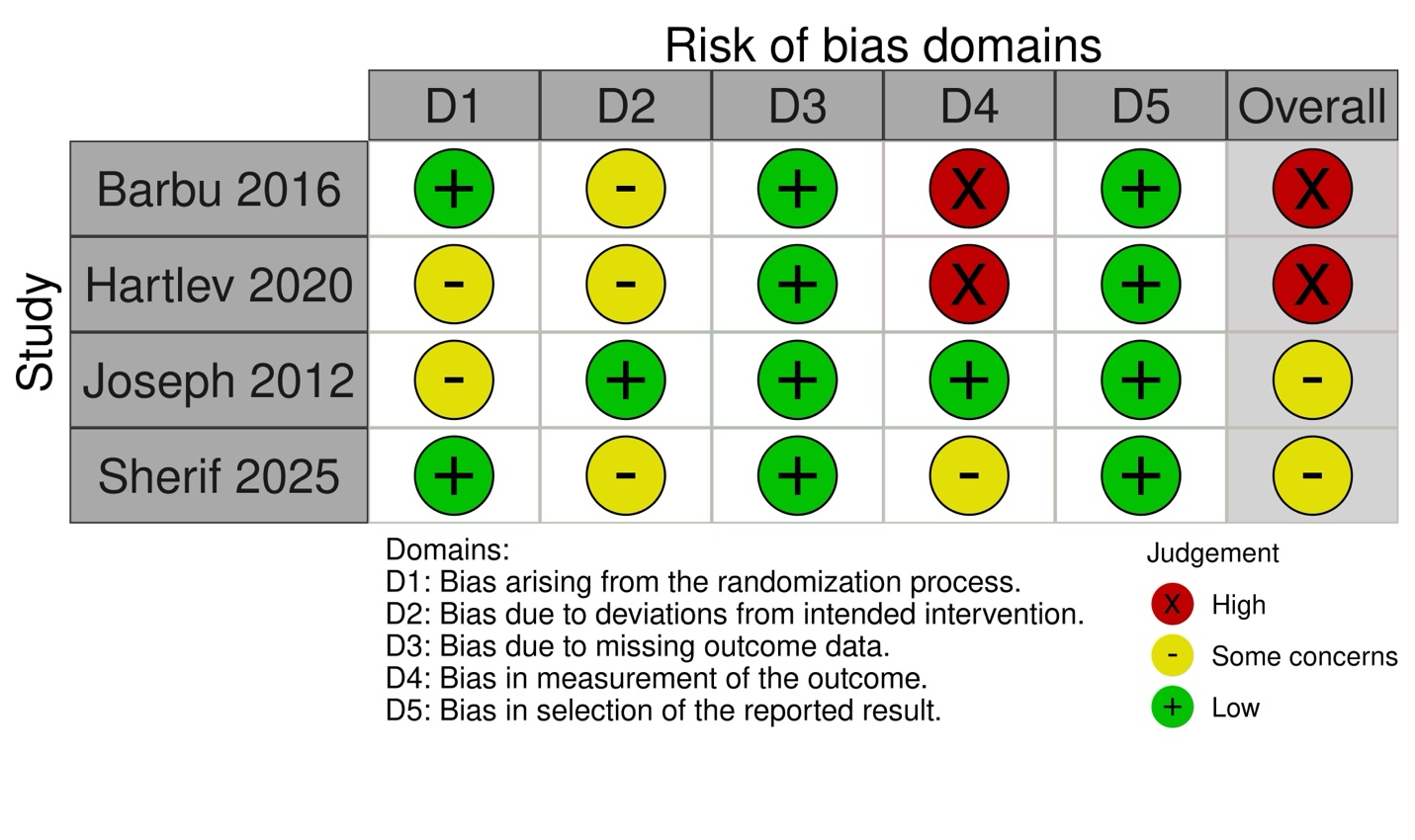


**Figure S9**. Traffic light plot showing ROB-II assessment of the included studies regarding pain reduction with PRF for periodontal/bone procedures.


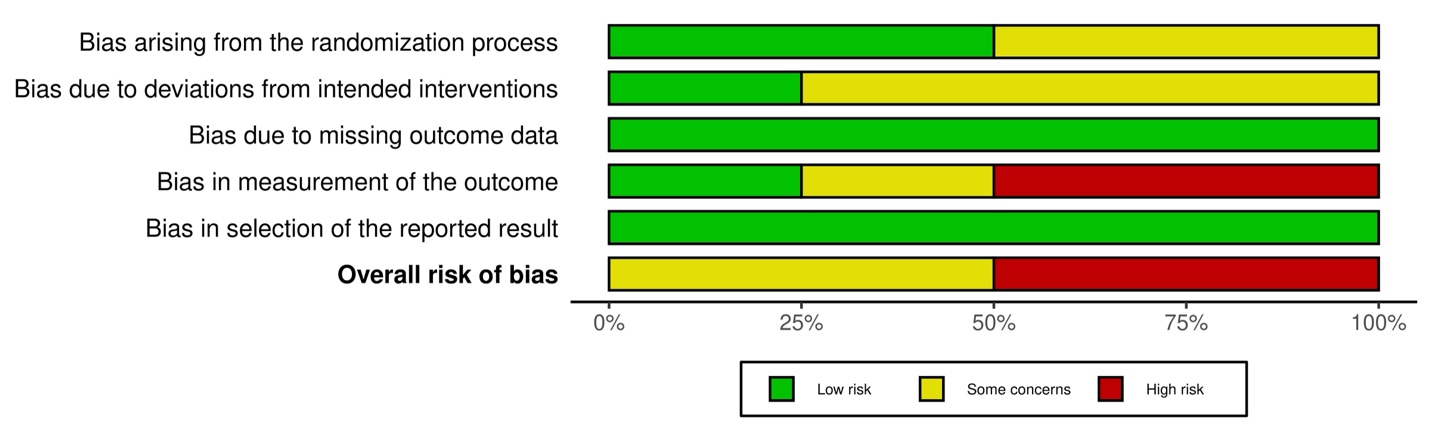


**Figure S10.** Summary plot showing ROB-II assessment of the included studies regarding pain reduction with PRF for periodontal/bone procedures.


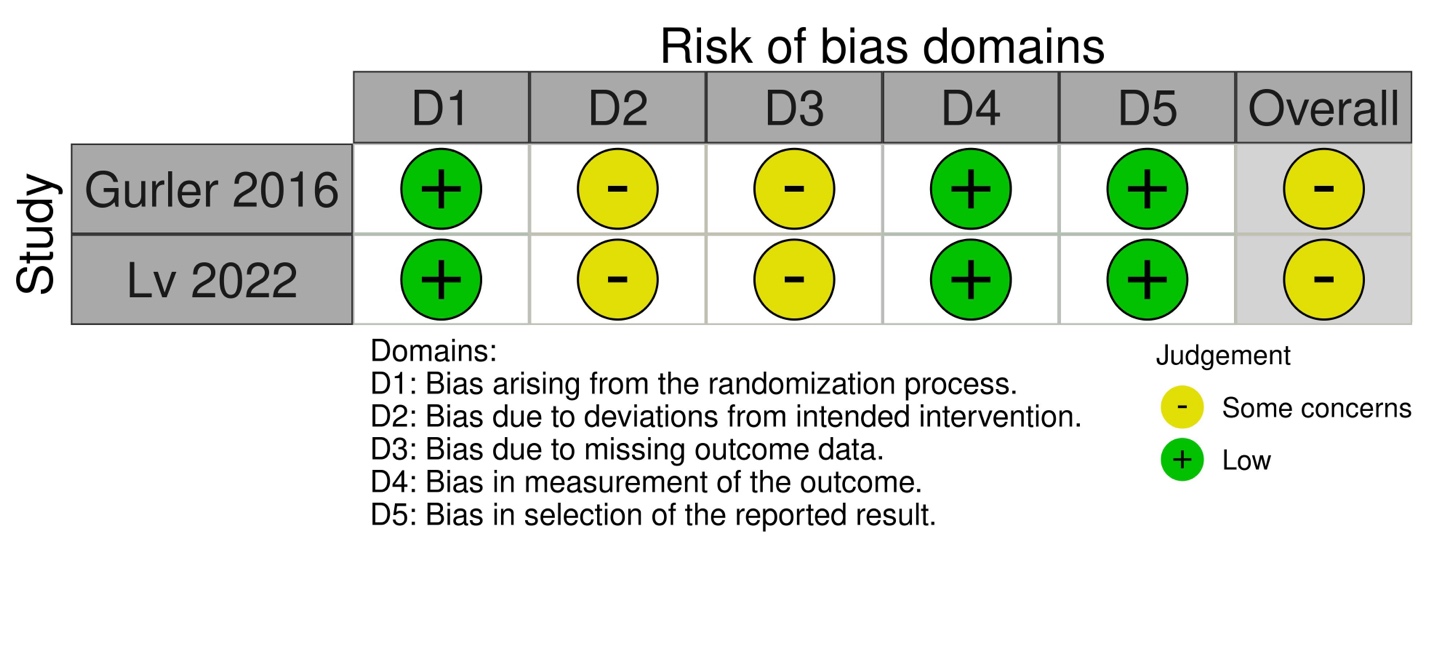


**Figure S11**. Traffic light plot showing ROB-II assessment of the included studies regarding pain reduction with PRF for sinus lift procedures.


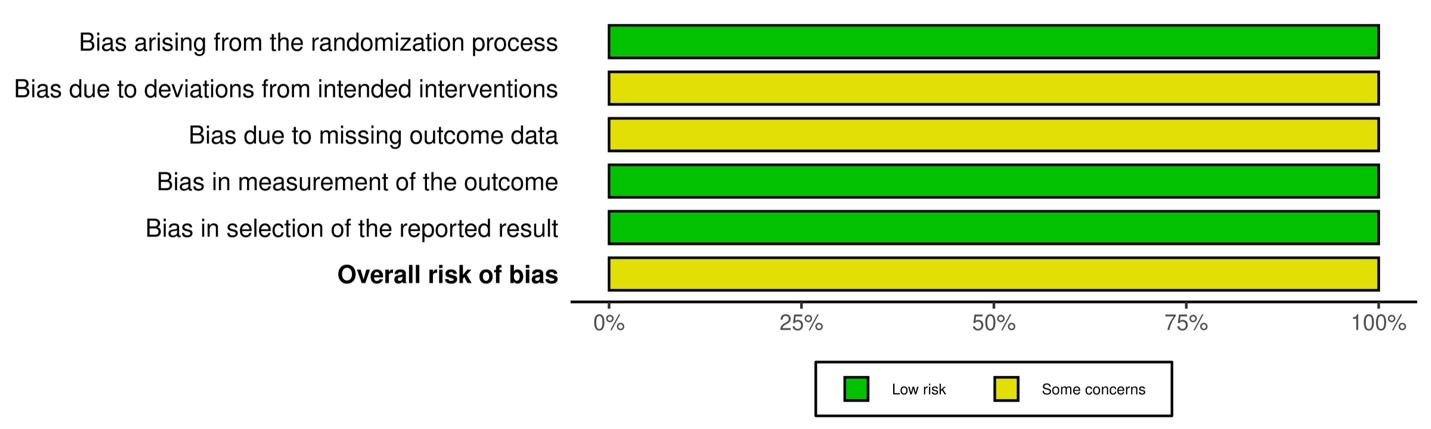


**Figure S12.** Summary plot showing ROB-II assessment of the included studies regarding pain reduction with PRF for sinus lift procedures.


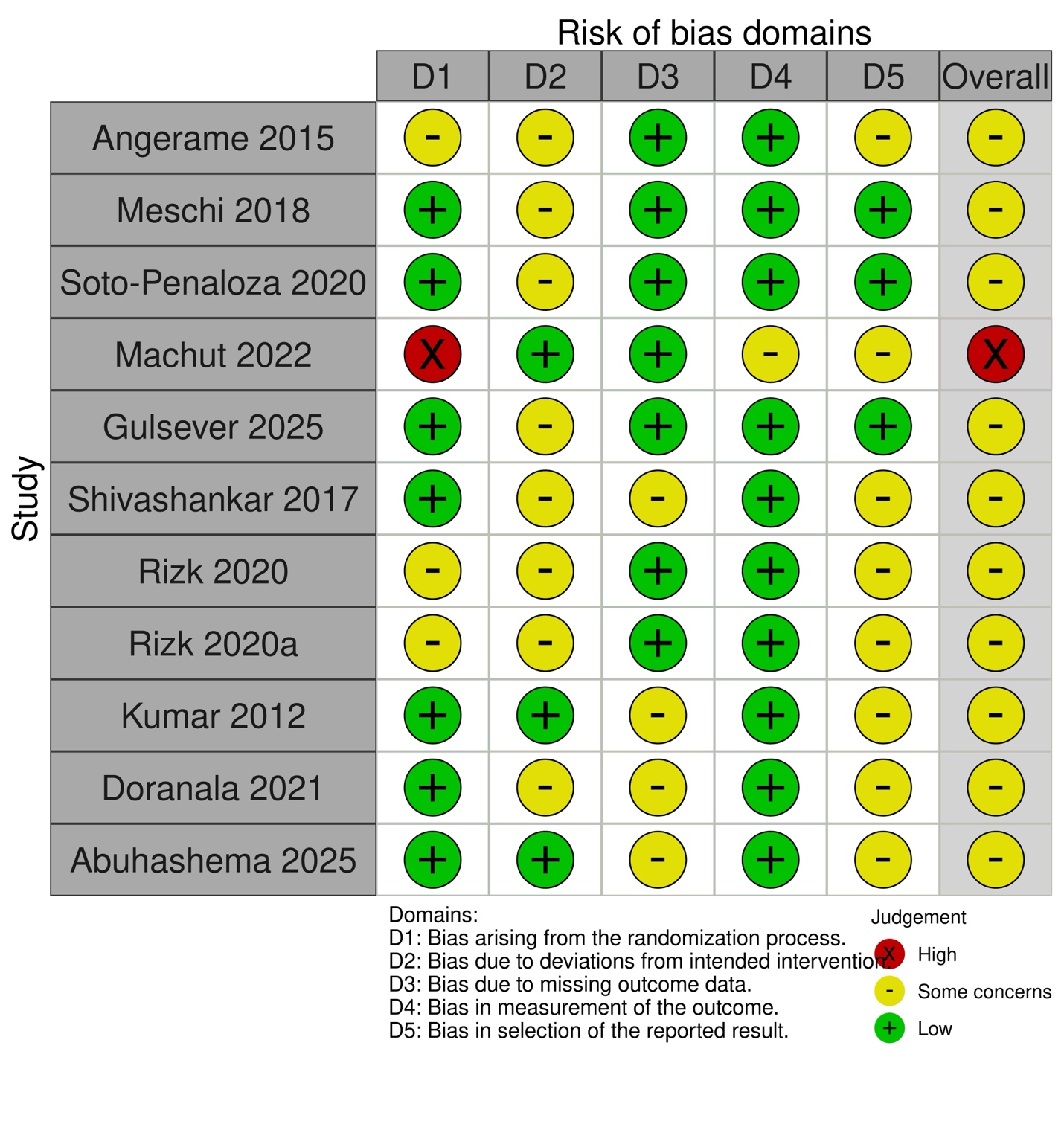


**Figure S13**. Traffic light plot showing ROB-II assessment of the included studies regarding pain reduction with PRF for endodontic procedures.


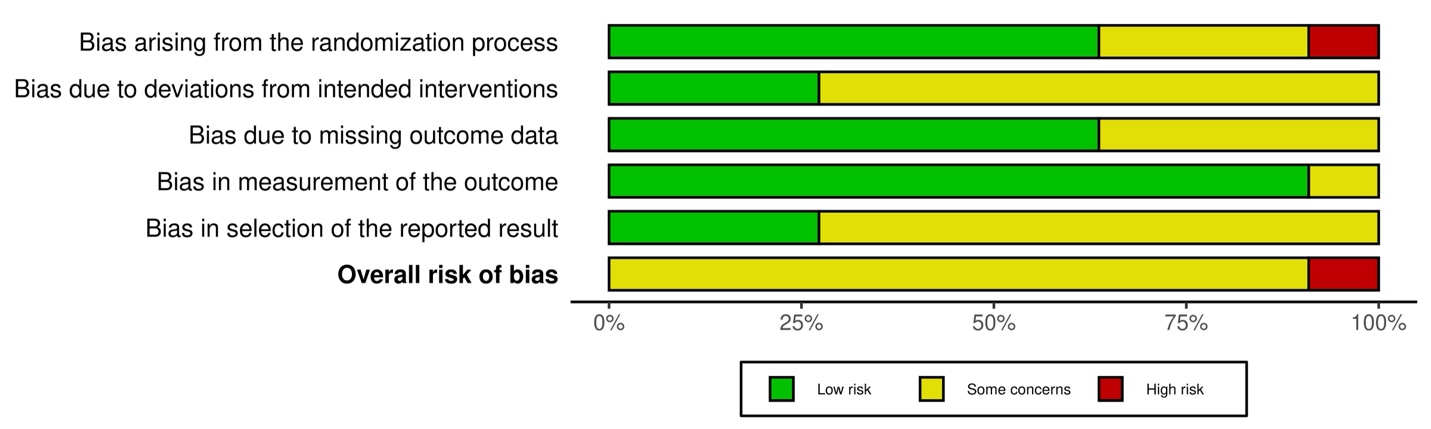


**Figure S14.** Summary plot showing ROB-II assessment of the included studies regarding pain reduction with PRF for endodontic procedures.


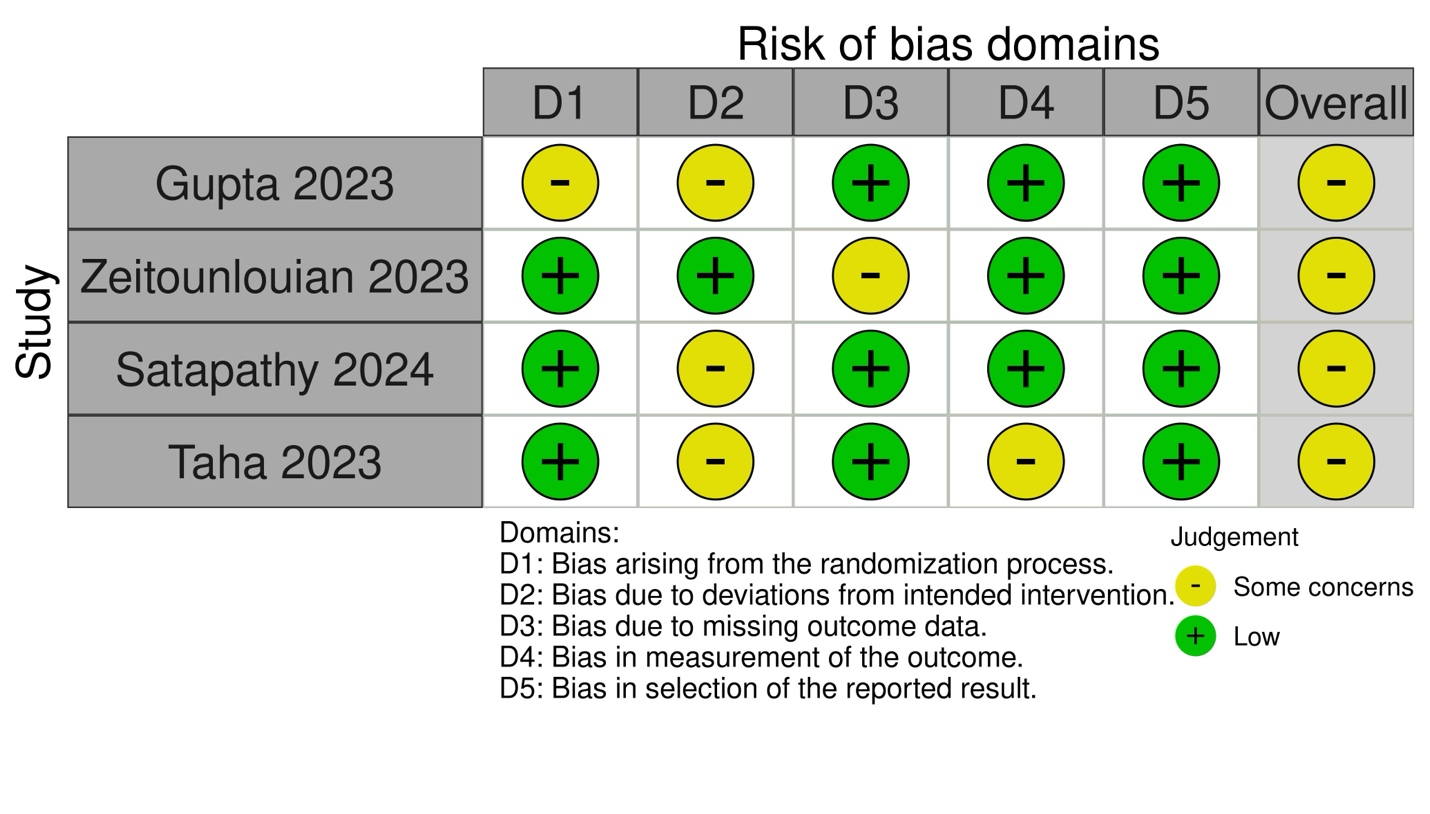


**Figure S15**. Traffic light plot showing ROB-II assessment of the included studies regarding pain reduction with PRF for orthodontic procedures.


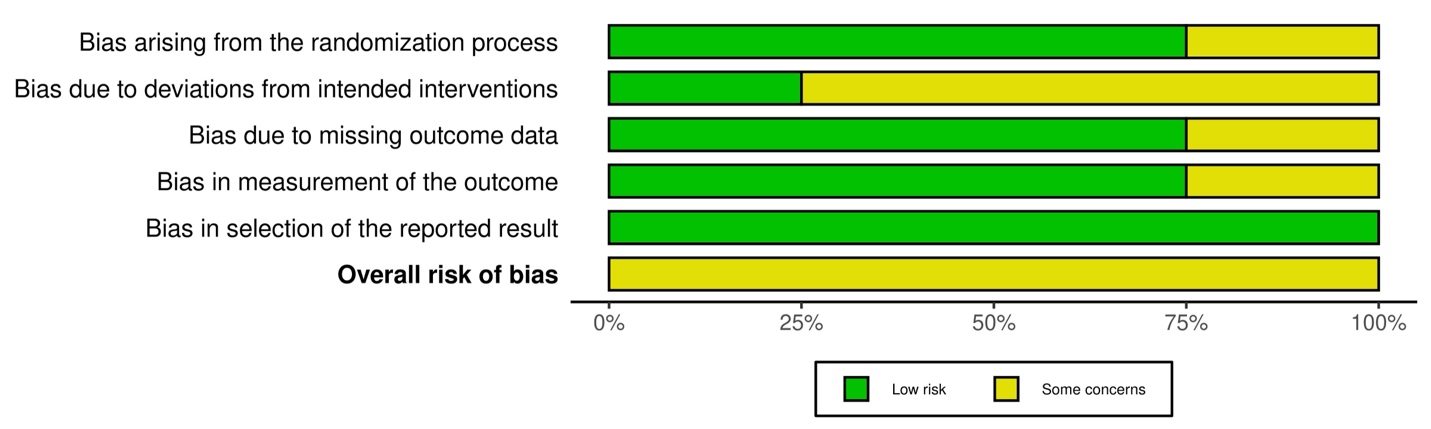


**Figure S16.** Summary plot showing ROB-II assessment of the included studies regarding pain reduction with PRF for orthodontic procedures.


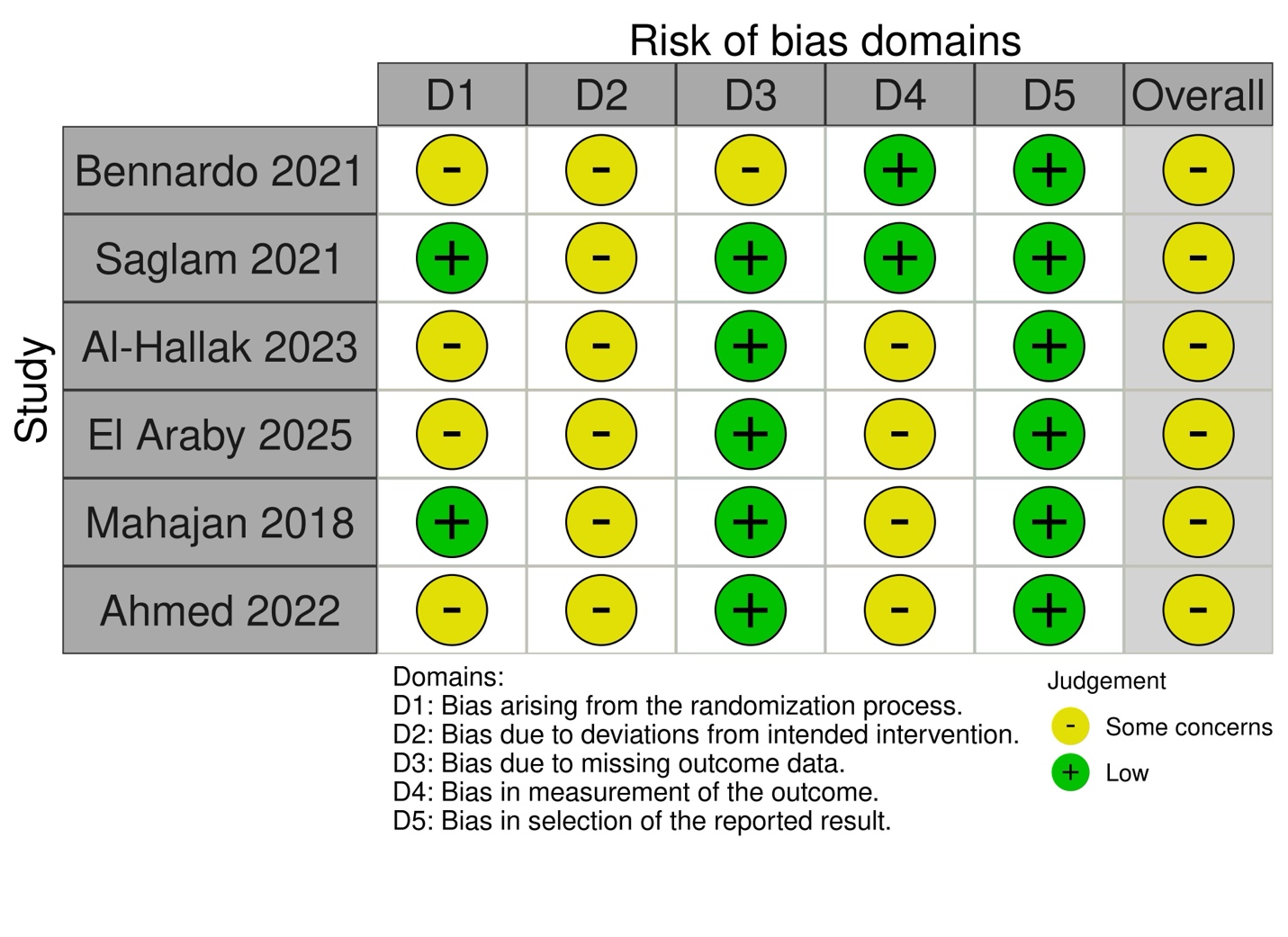


**Figure S17**. Traffic light plot showing ROB-II assessment of the included studies regarding pain reduction with PRF for oral lesions.


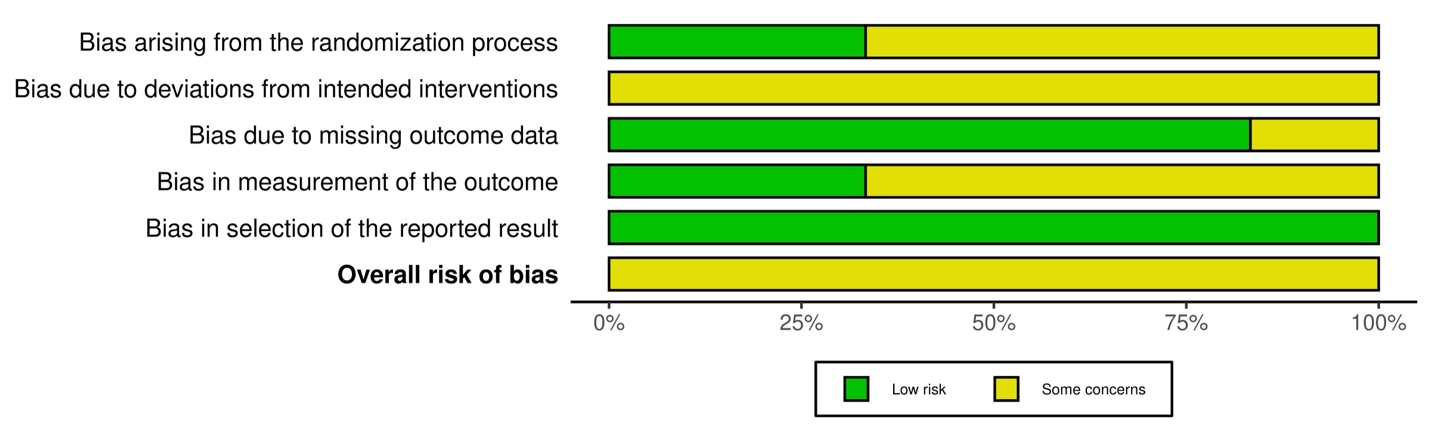


**Figure S18.** Summary plot showing ROB-II assessment of the included studies regarding pain reduction with PRF for oral lesions.


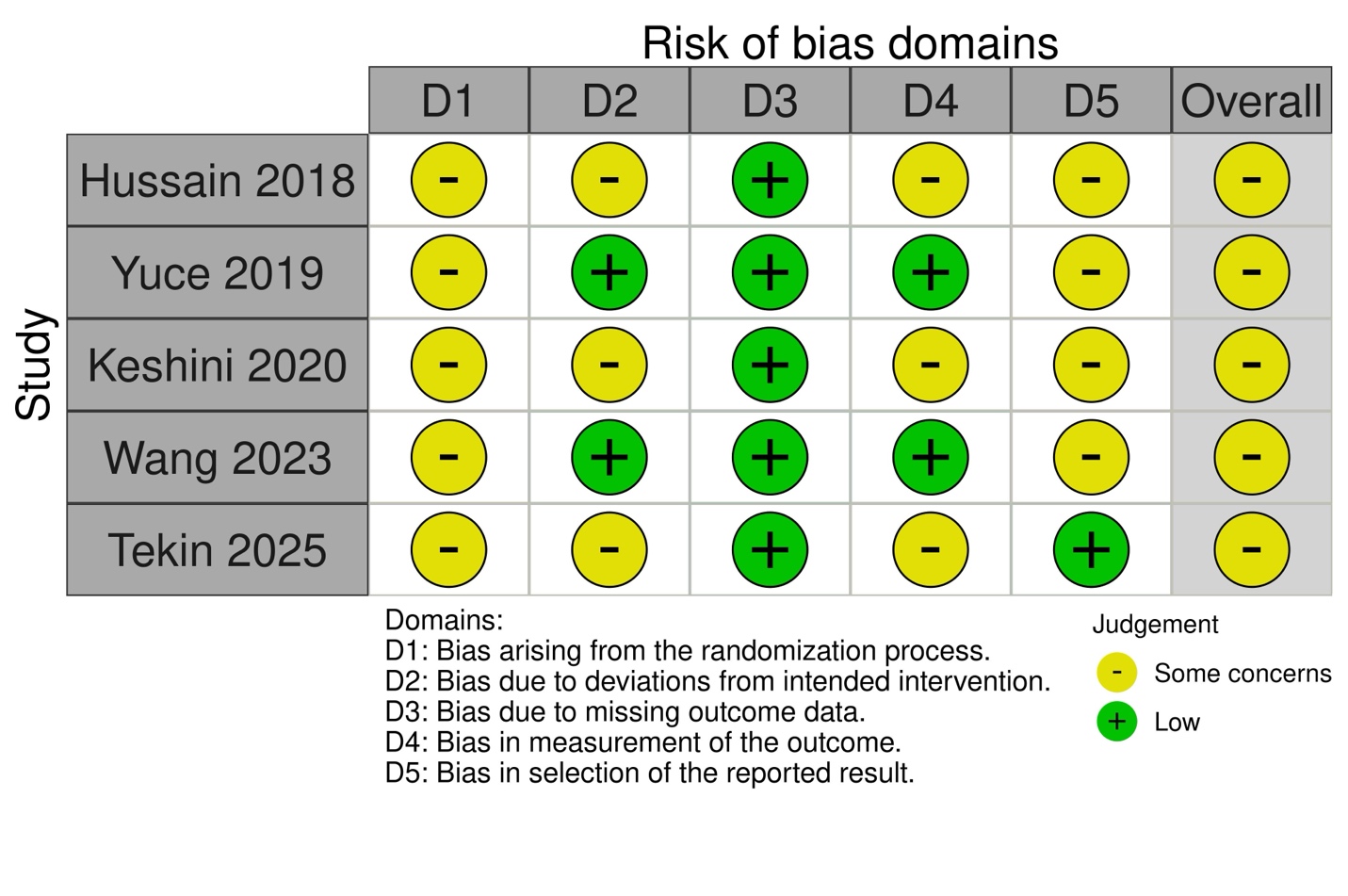


**Figure S19**. Traffic light plot showing ROB-II assessment of the included studies regarding pain reduction with PRF for alveolar osteitis (dry socket).


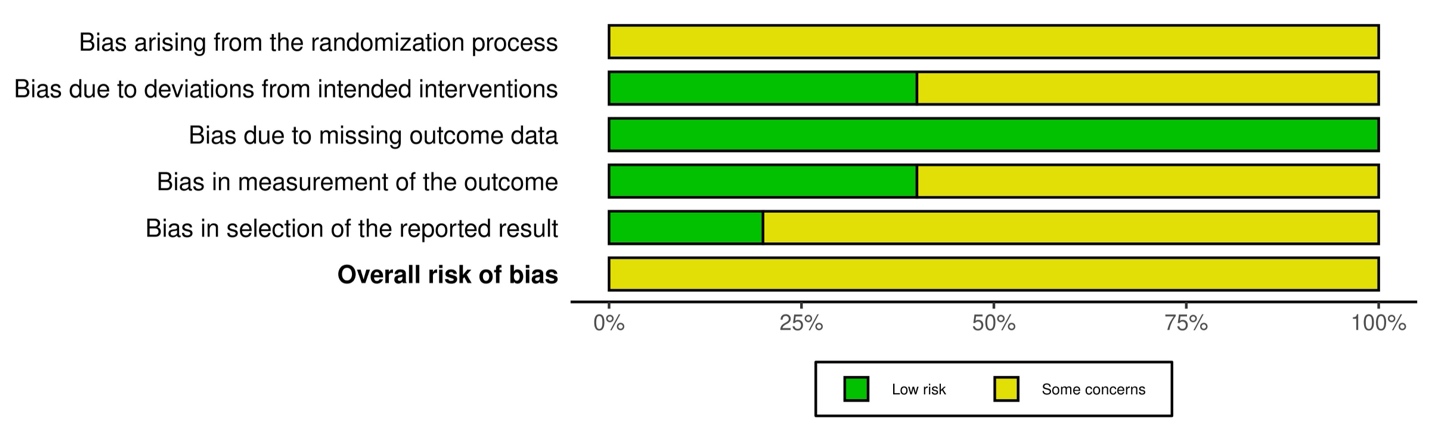


**Figure S20.** Summary plot showing ROB-II assessment of the included studies regarding pain reduction with PRF for alveolar osteitis (dry socket).


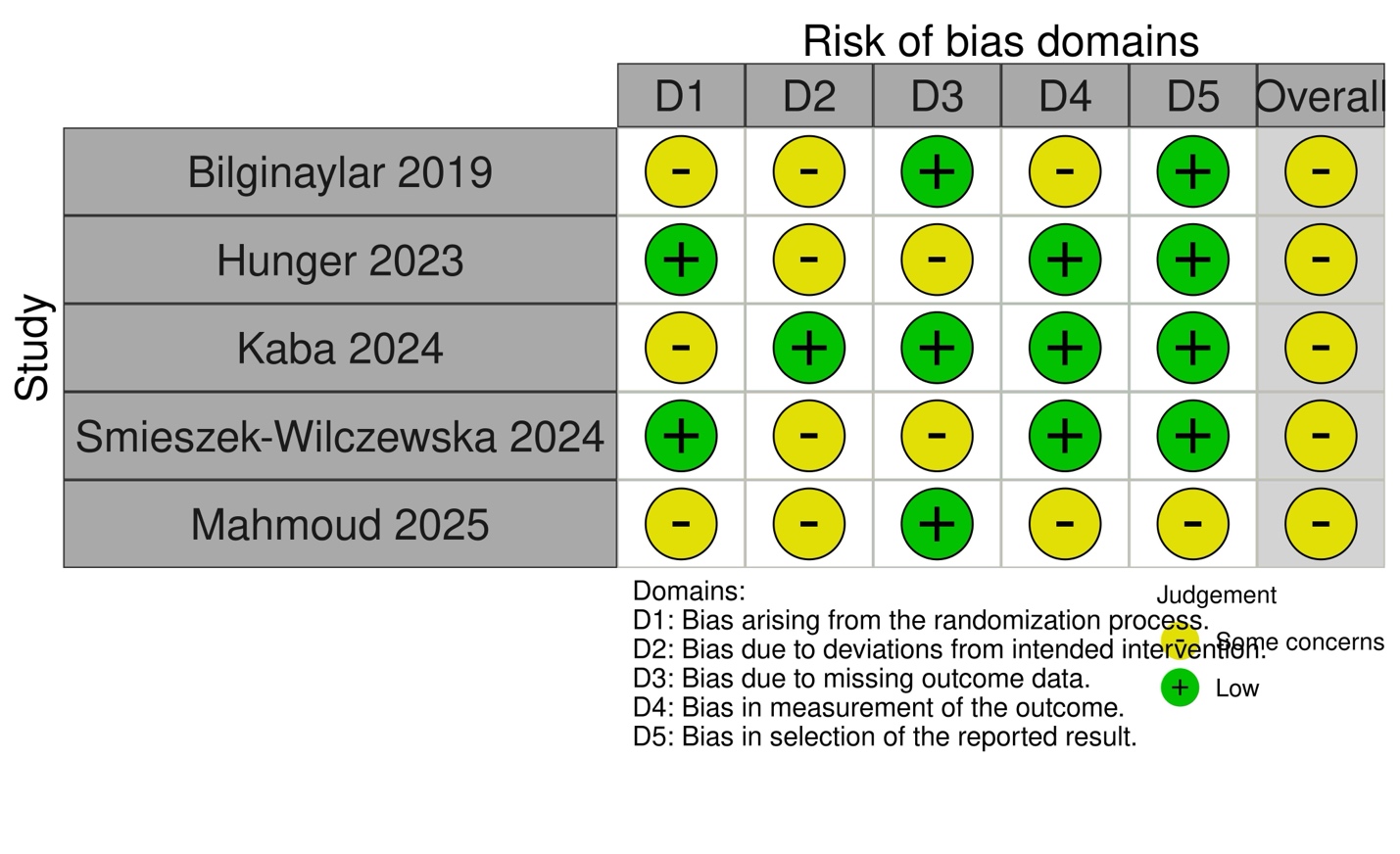


**Figure S21**. Traffic light plot showing ROB-II assessment of the included studies regarding pain reduction with PRF for oro-antral communications.


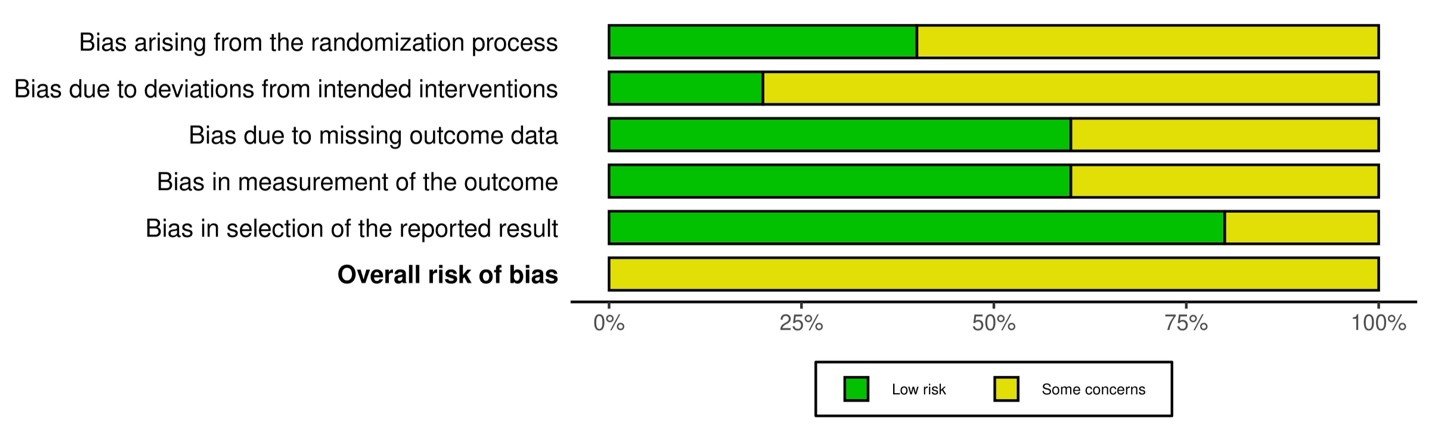


**Figure S22.** Summary plot showing ROB-II assessment of the included studies regarding pain reduction with PRF for oro-antral communications.


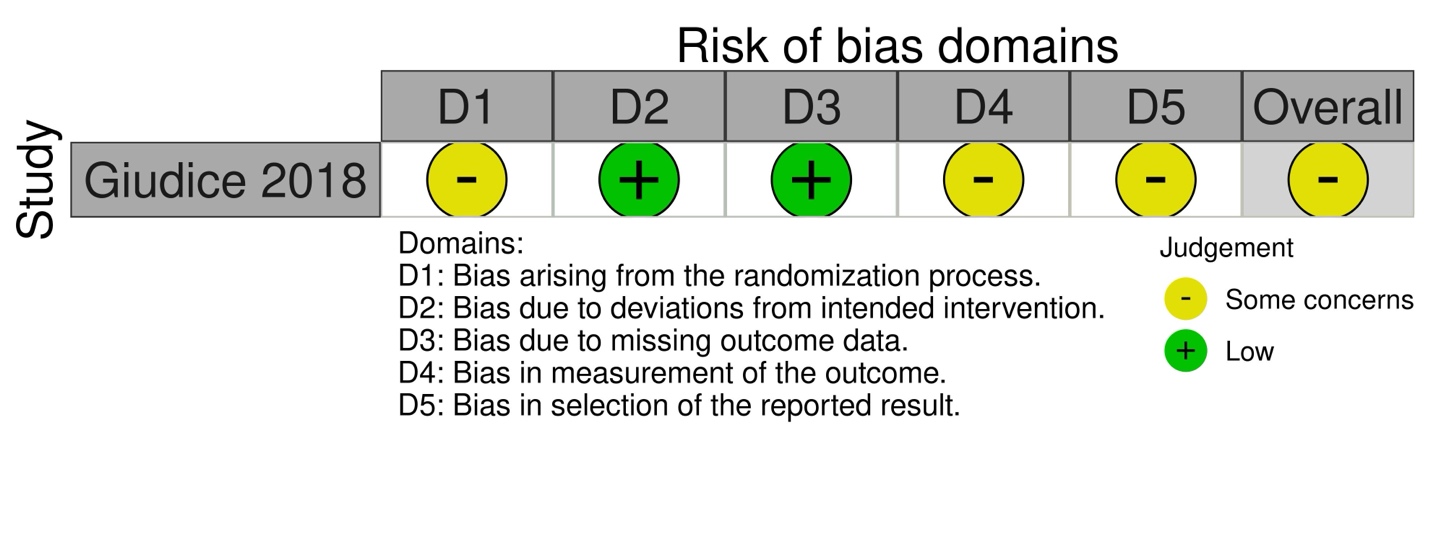


**Figure S23**. Traffic light plot showing ROB-II assessment of the included studies regarding pain reduction with PRF for MRONJ.


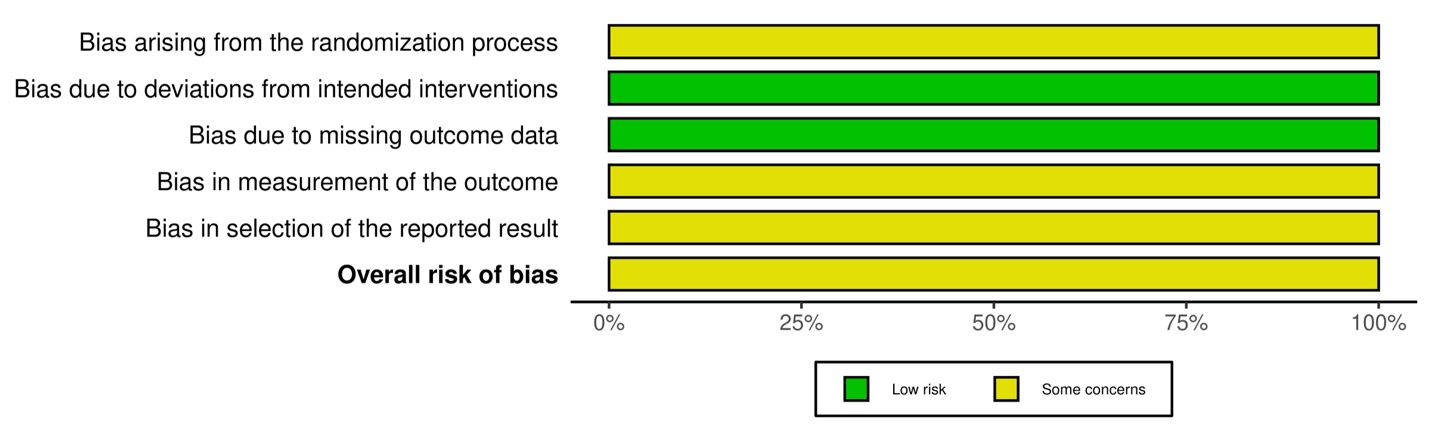


**Figure S24.** Summary plot showing ROB-II assessment of the included studies regarding pain reduction with PRF for MRONJ.


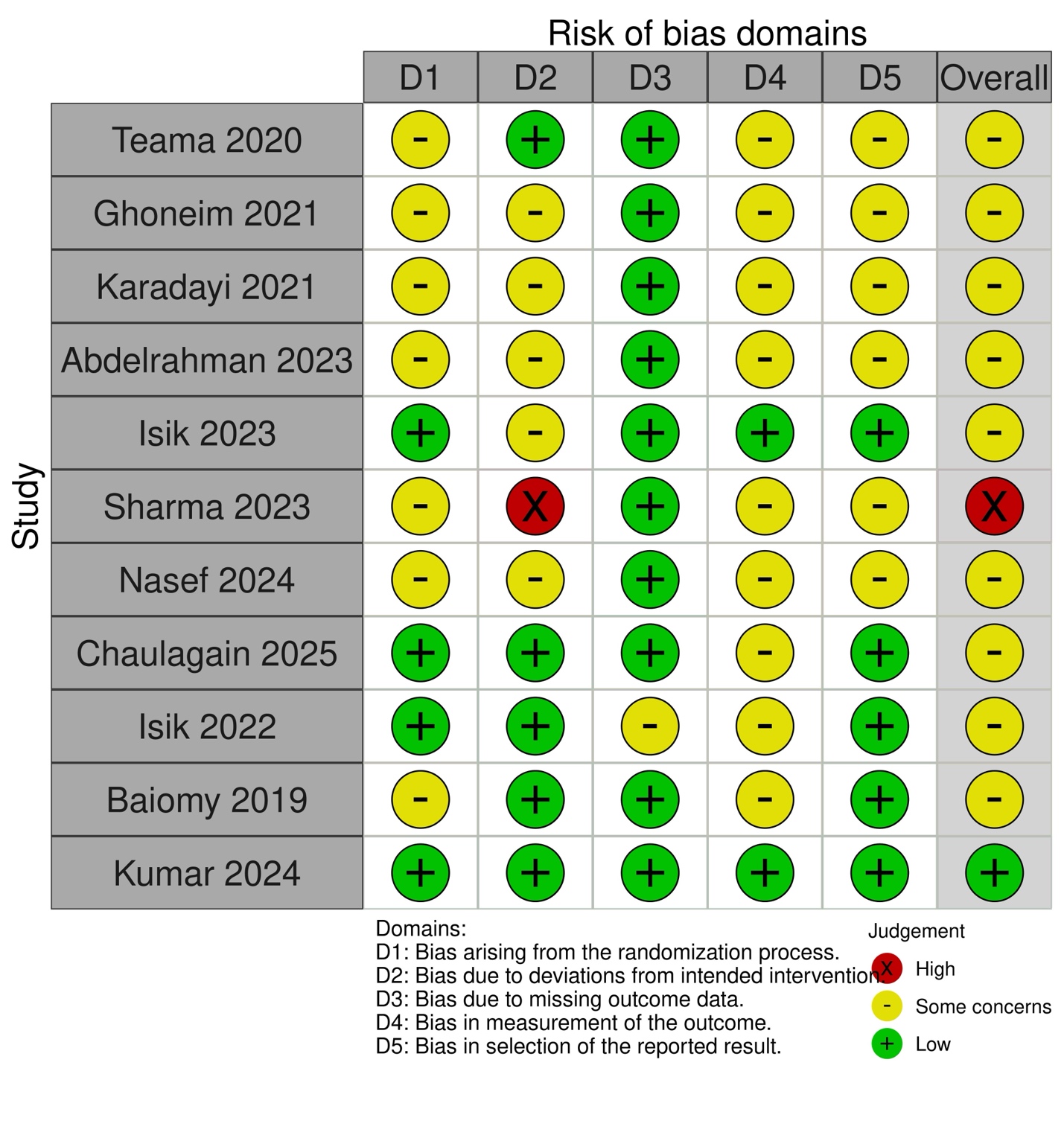


**Figure S25.** Traffic light plot showing ROB-II assessment of the included studies regarding pain reduction with PRF for TMJ disorders.


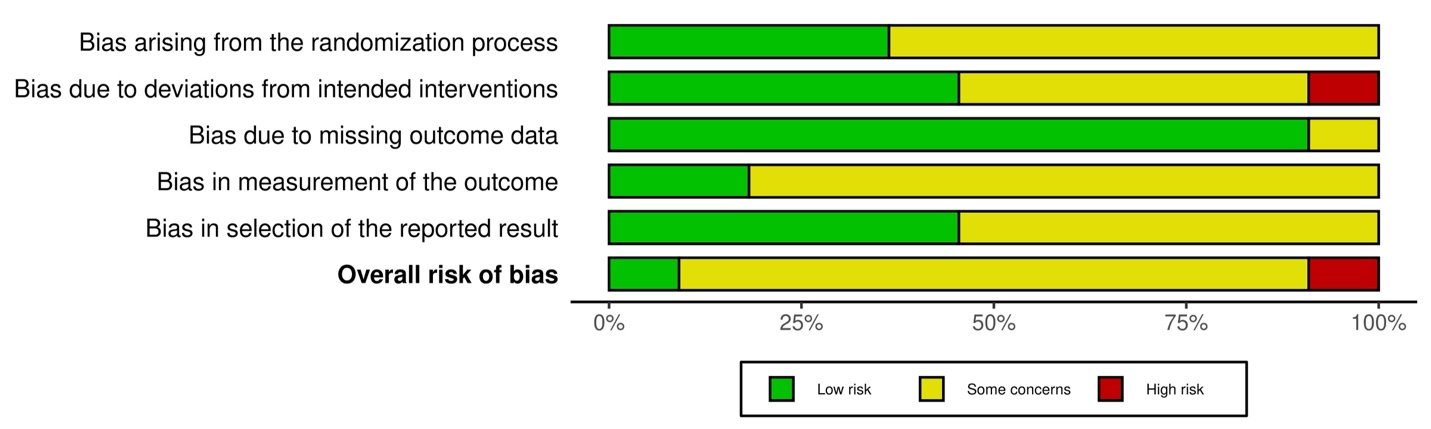


**Figure S26.** Summary plot showing ROB-II assessment of the included studies regarding pain reduction with PRF for TMJ disorders.


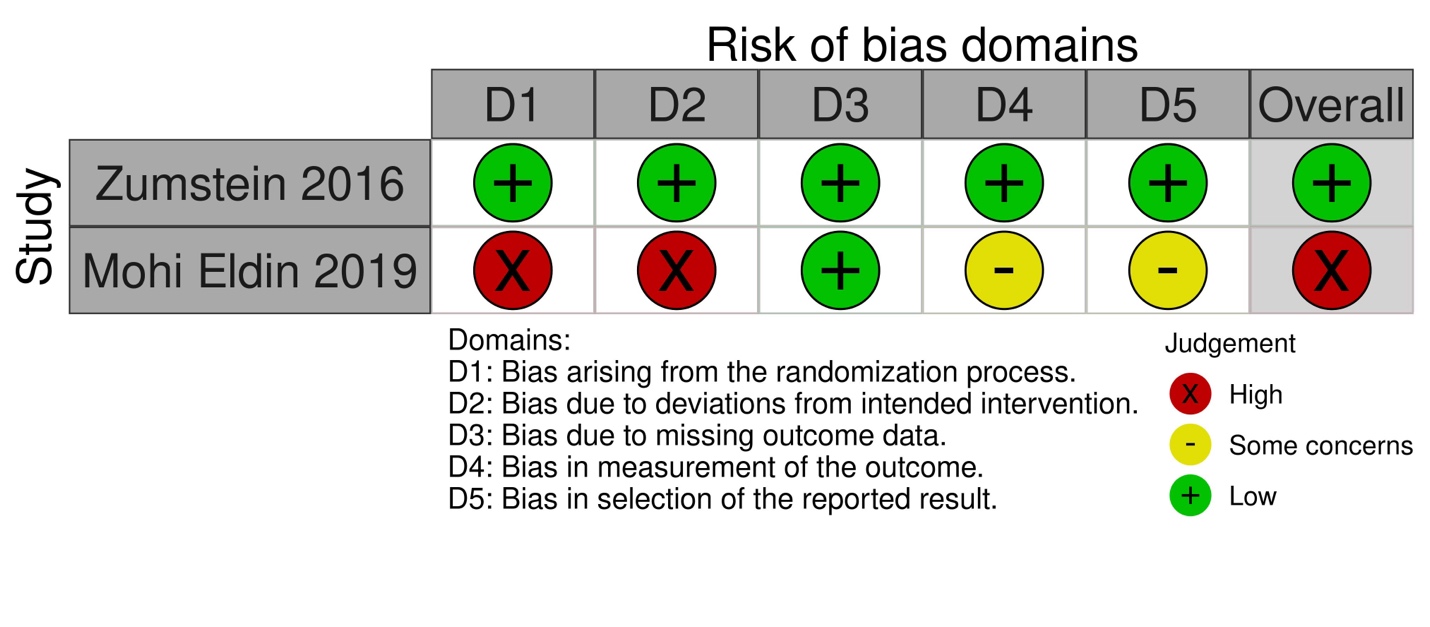


**Figure S27.** Traffic light plot showing ROB-II assessment of the included studies regarding pain reduction with PRF for orthopedics.


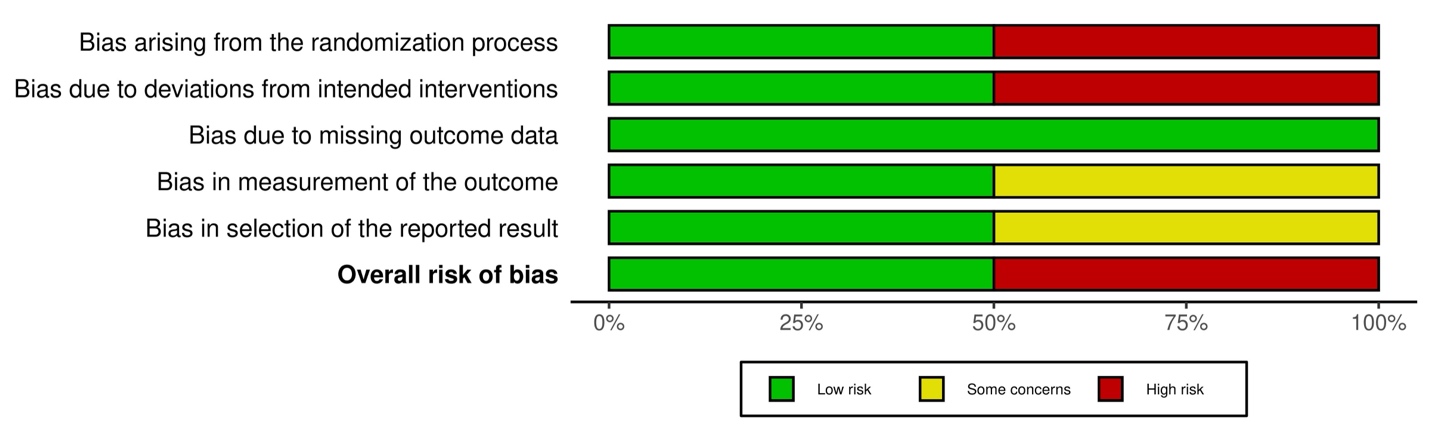


**Figure S28.** Summary plot showing ROB-II assessment of the included studies regarding pain reduction with PRF for orthopedics.


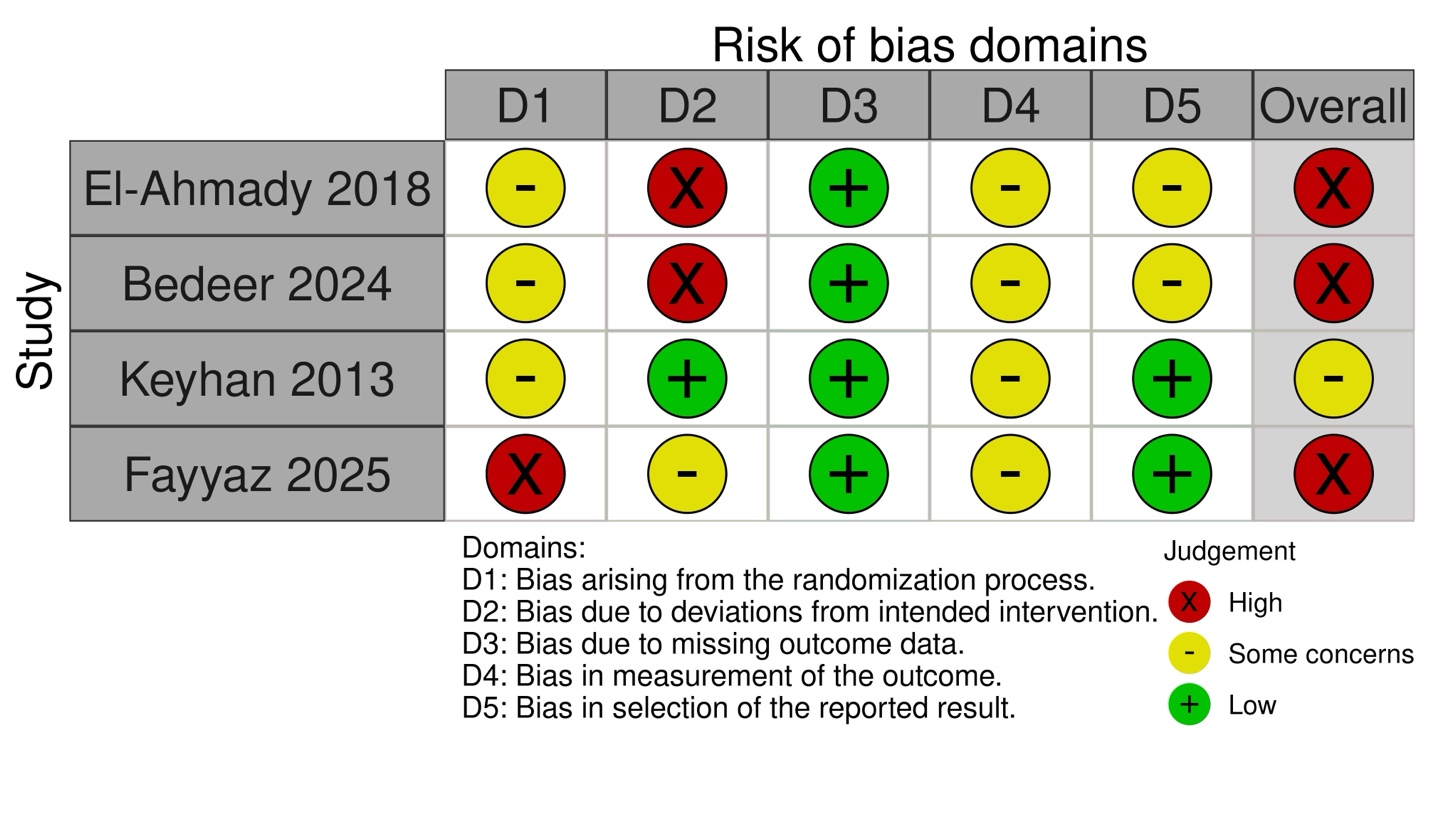


**Figure S29.** Traffic light plot showing ROB-II assessment of the included studies regarding pain reduction with PRF for facial surgery and esthetics.


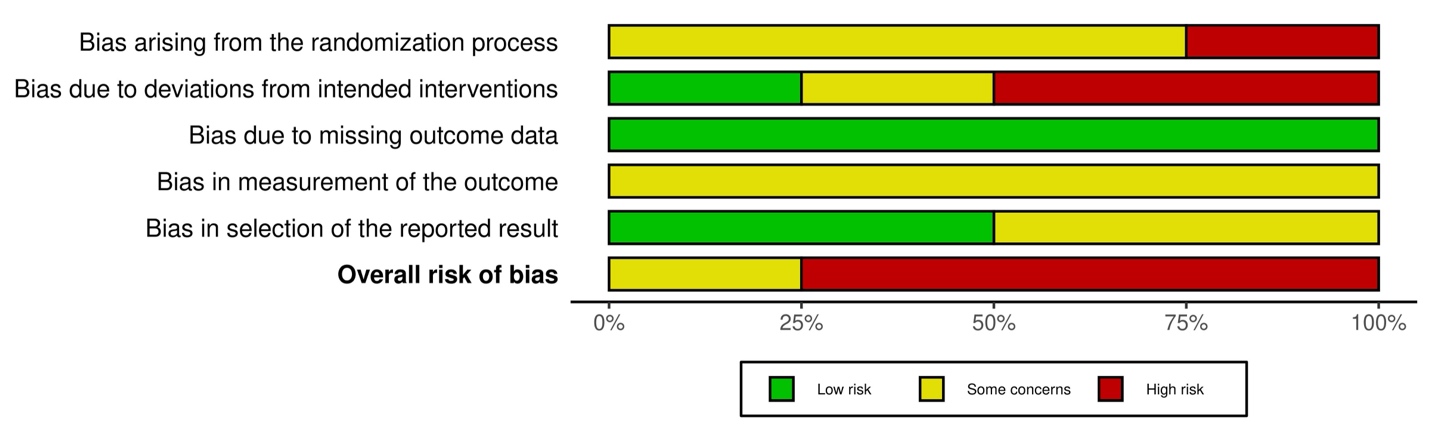


**Figure S30.** Summary plot showing ROB-II assessment of the included studies regarding pain reduction with PRF for facial surgery and esthetics.


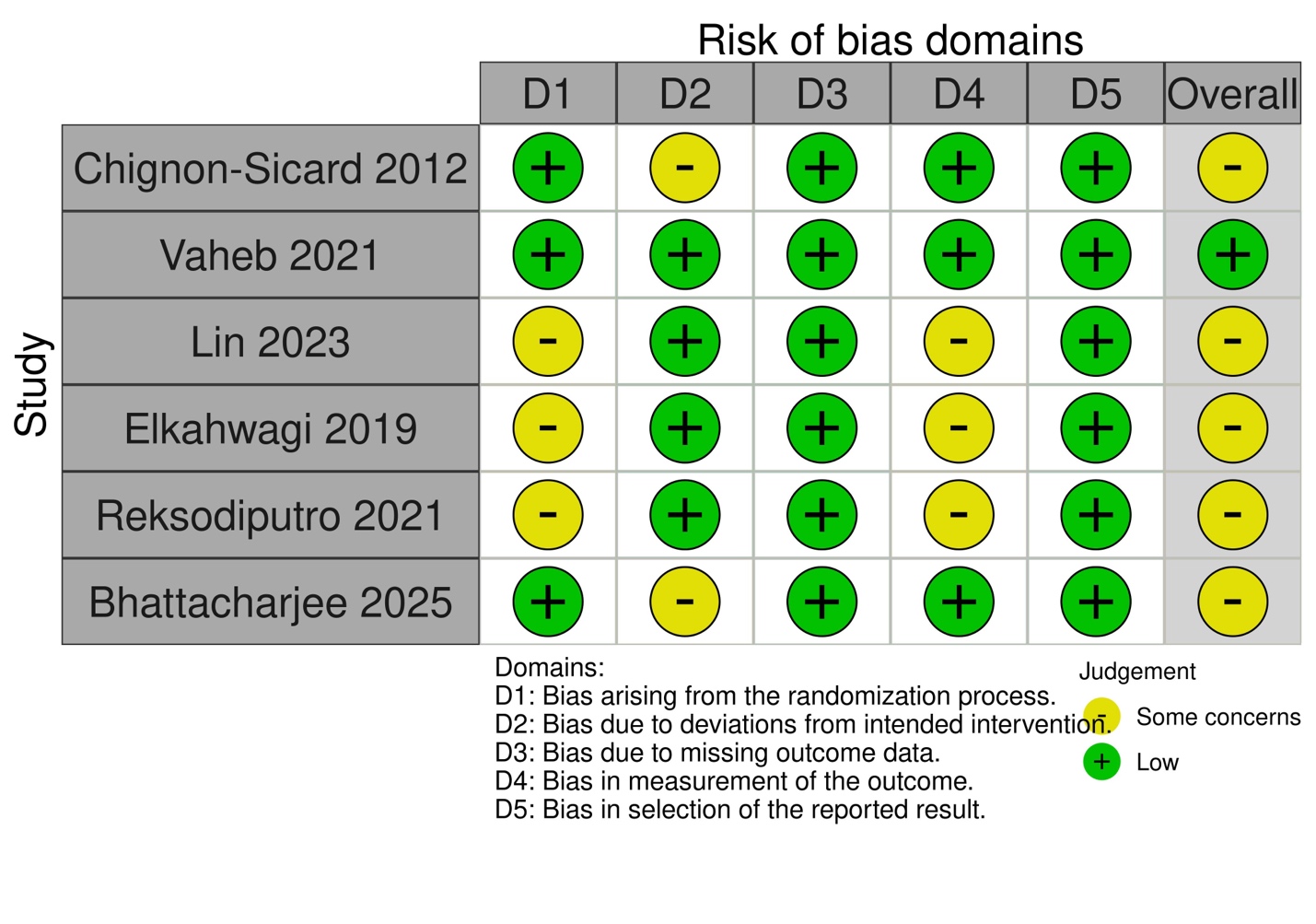


**Figure S31.** Traffic light plot showing ROB-II assessment of the included studies regarding pain reduction with PRF for miscellaneous medical procedures.


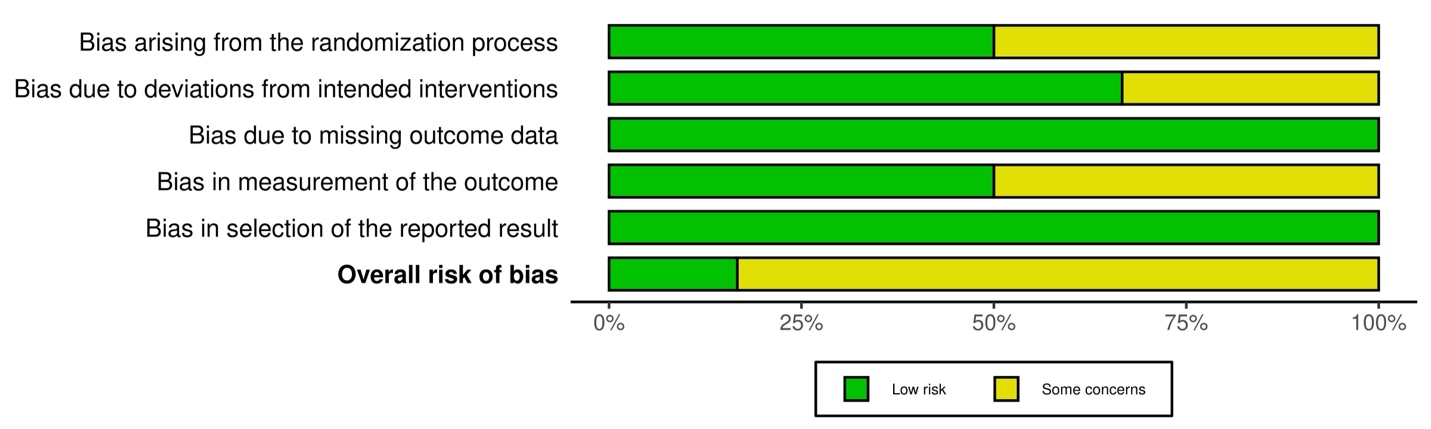


**Figure S32.** Summary plot showing ROB-II assessment of the included studies regarding pain reduction with PRF for miscellaneous medical procedures.


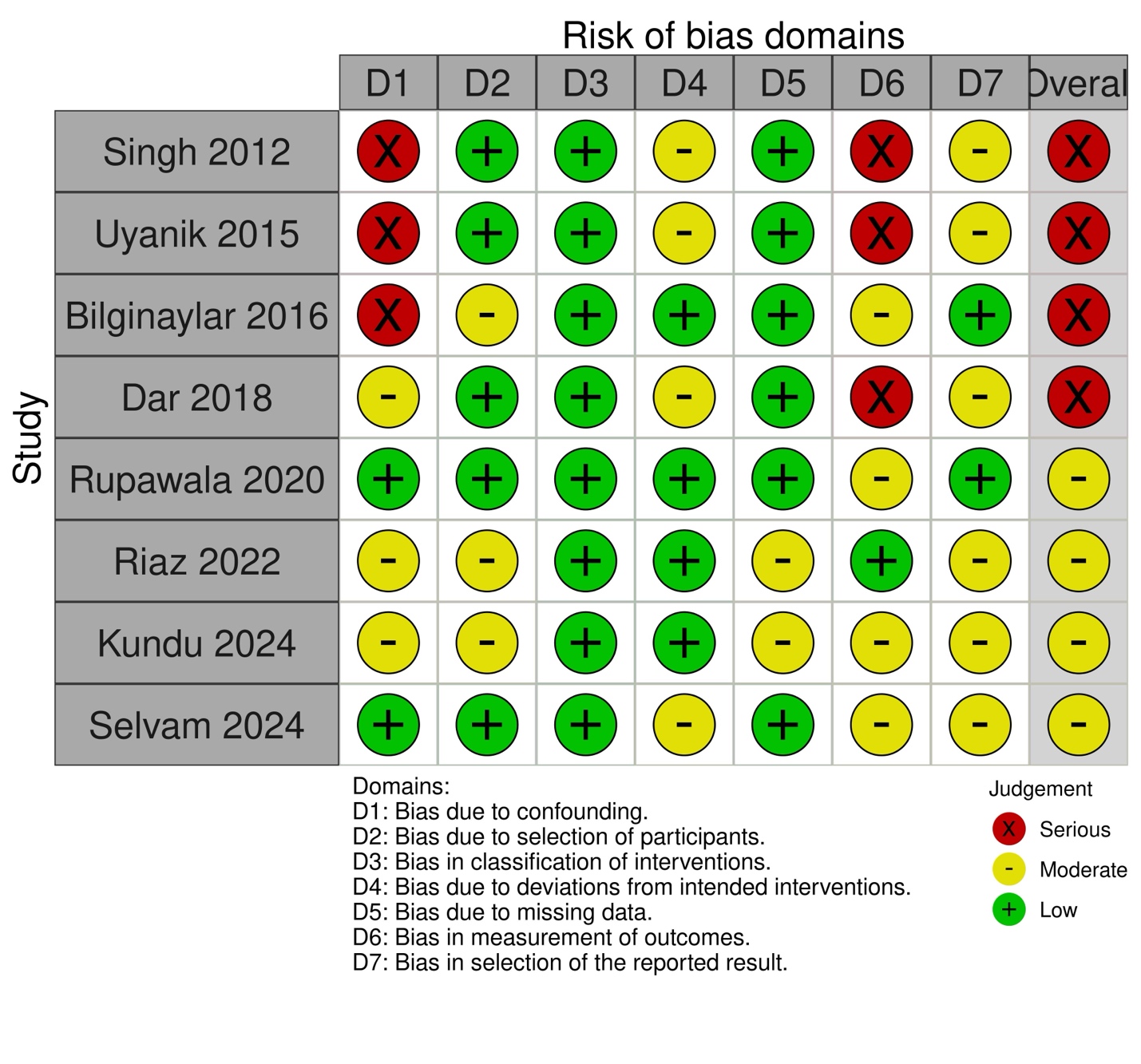


**Figure S33.** Traffic light plot showing ROBINS-I assessment of the included studies regarding pain reduction with PRF for 3^rd^ molar extractions.


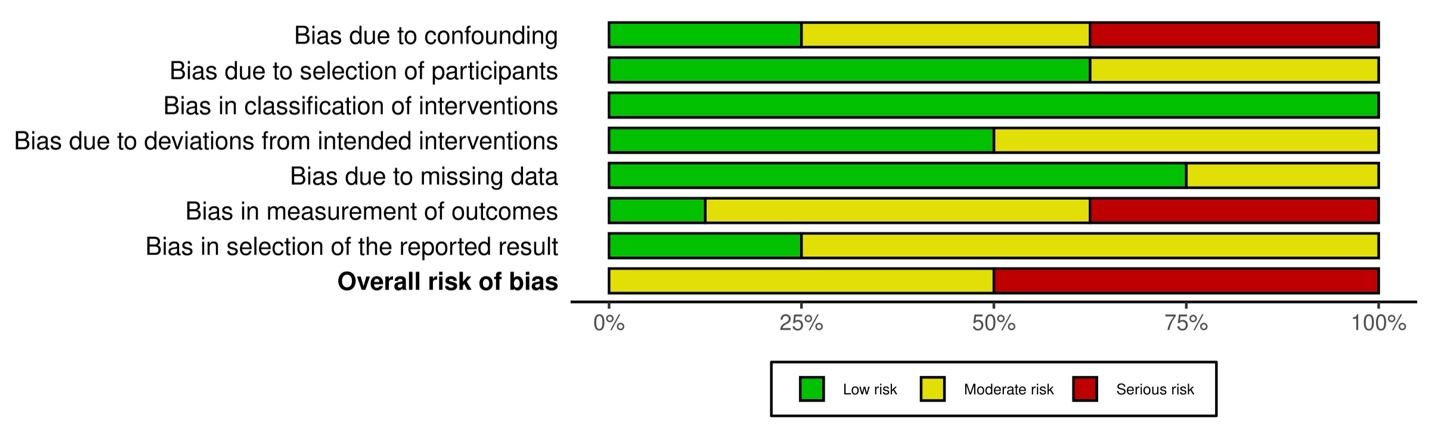


**Figure S34.** Summary plot showing ROBINS-I assessment of the included studies regarding pain reduction with PRF for 3^rd^ molar extractions.


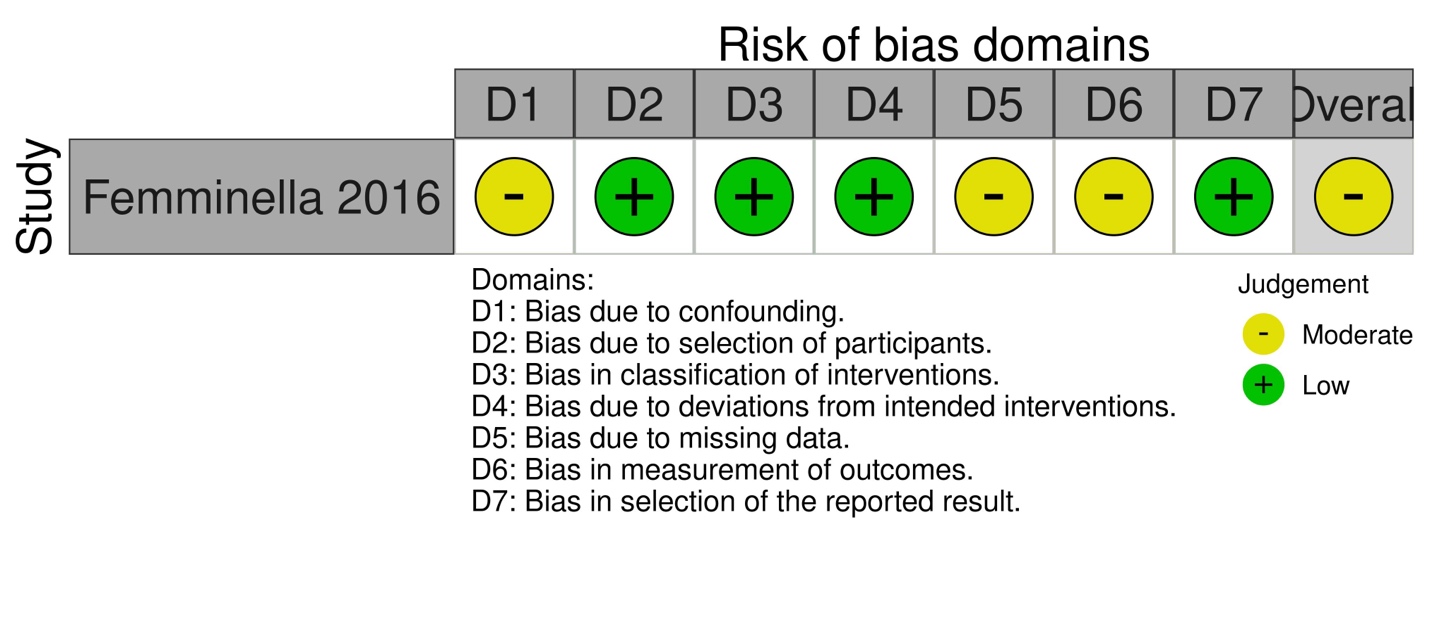


**Figure S35**. Traffic light plot showing ROBINS-I assessment of the included studies regarding pain reduction with PRF for palatal wound healing.


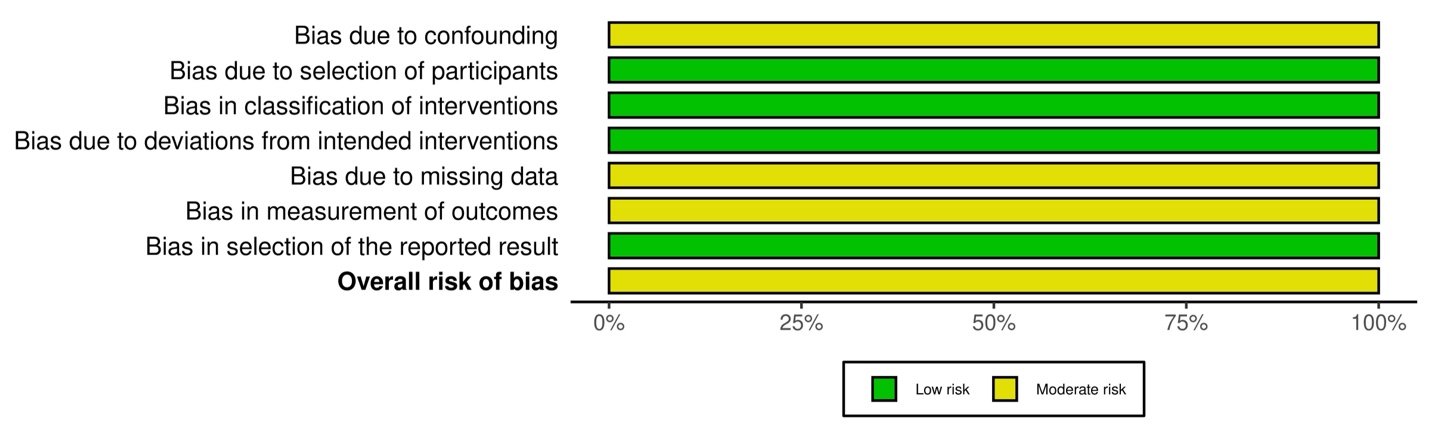


**Figure S36.** Summary plot showing ROBINS-I assessment of the included studies regarding pain reduction with PRF for palatal wound healing.


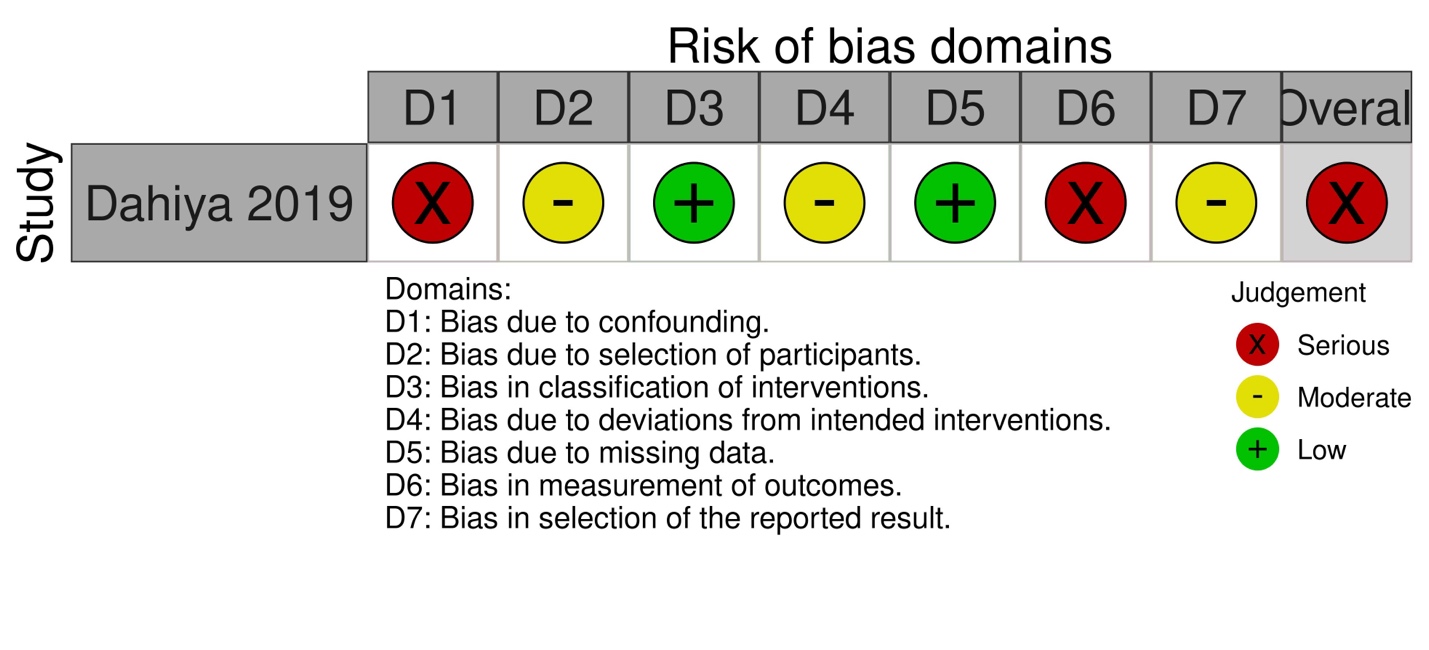


**Figure S37**. Traffic light plot showing ROBINS-I assessment of the included studies regarding pain reduction with PRF for mucogingival conditions.


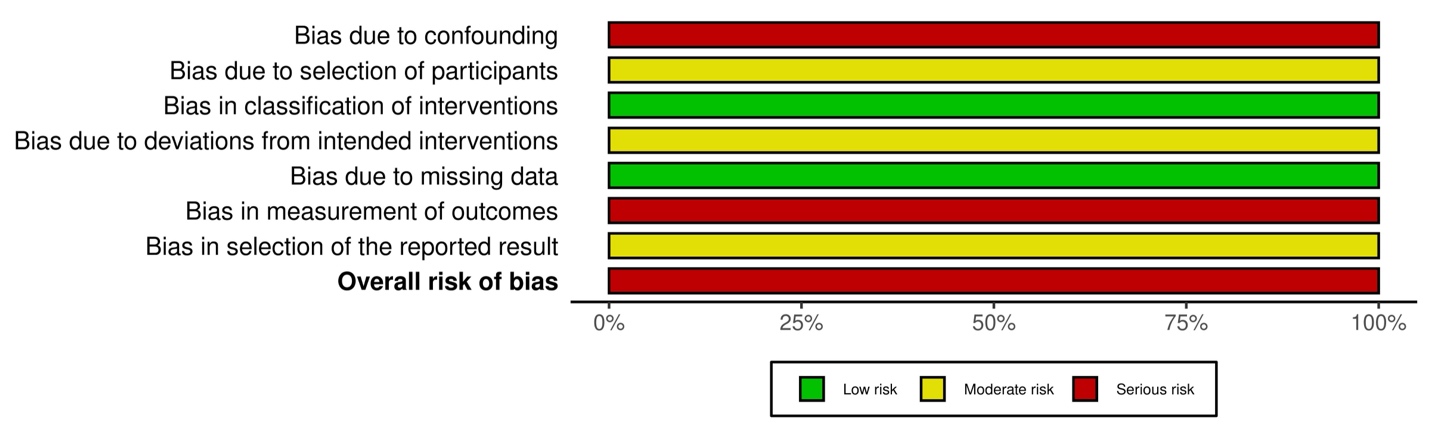


**Figure S38.** Summary plot showing ROBINS-I assessment of the included studies regarding pain reduction with PRF for mucogingival conditions.


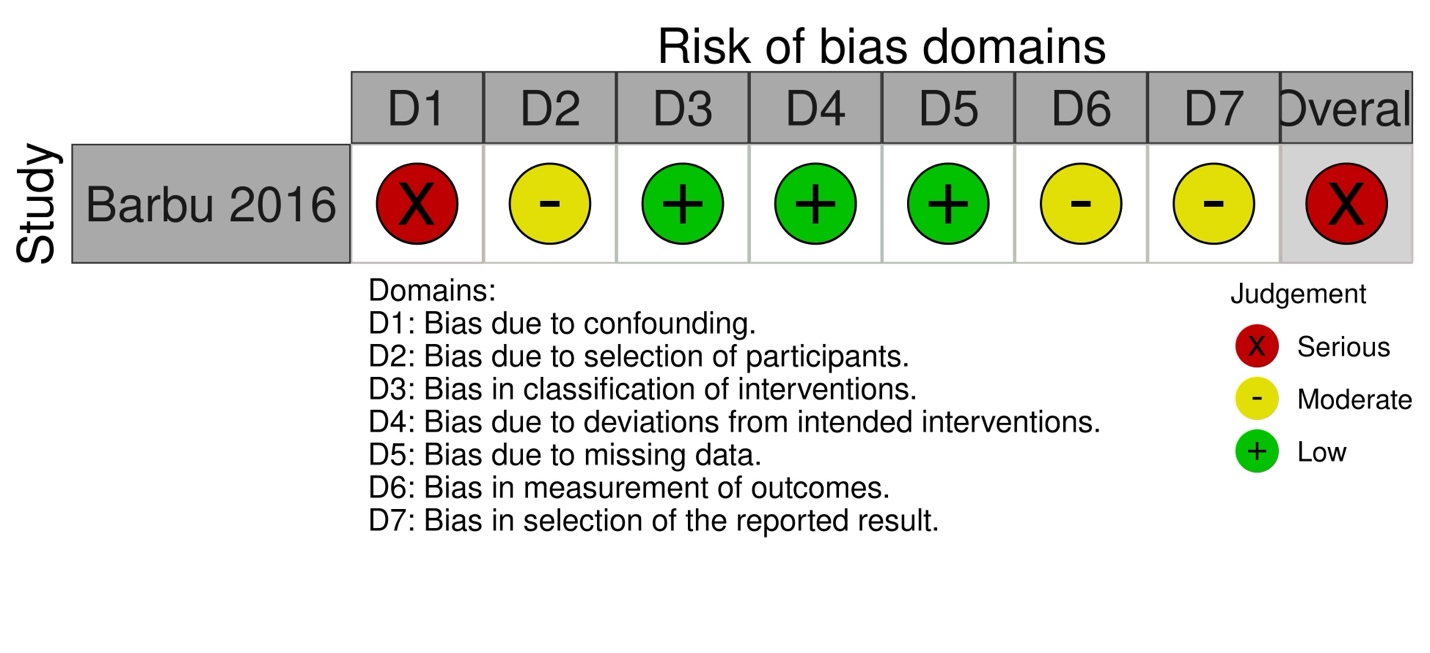


**Figure S39**. Traffic light plot showing ROBINS-I assessment of the included studies regarding pain reduction with PRF for periodontal/bone procedures.


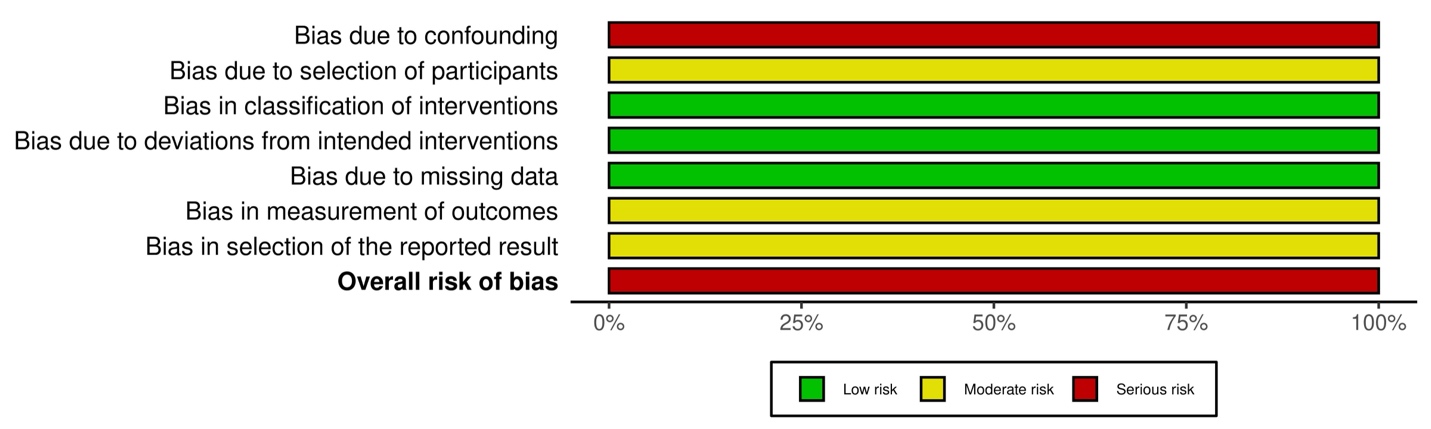


**Figure S40.** Summary plot showing ROBINS-I assessment of the included studies regarding pain reduction with PRF for periodontal/bone procedures


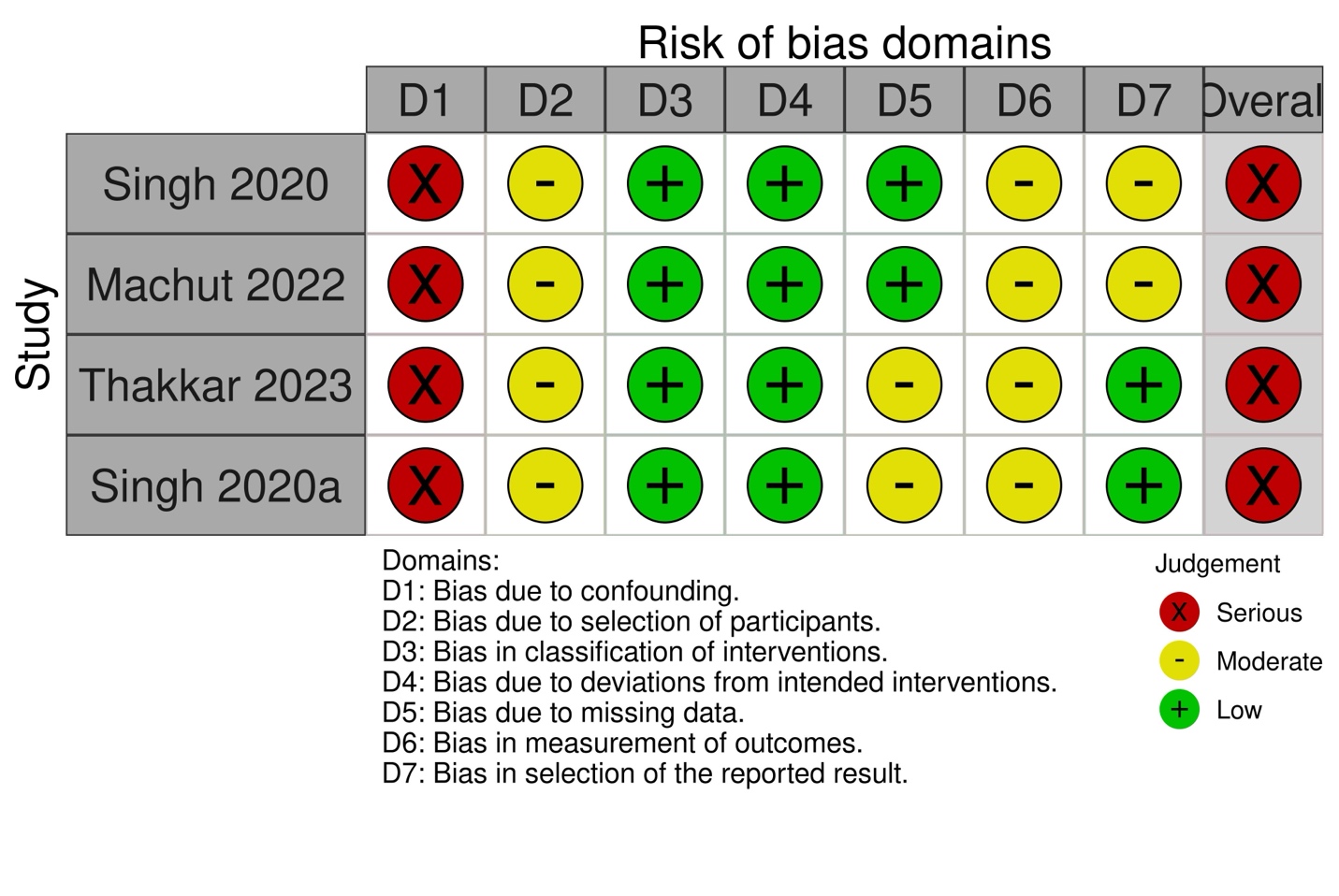


**Figure S41**. Traffic light plot showing ROBINS-I assessment of the included studies regarding pain reduction with PRF for endodontic procedures.


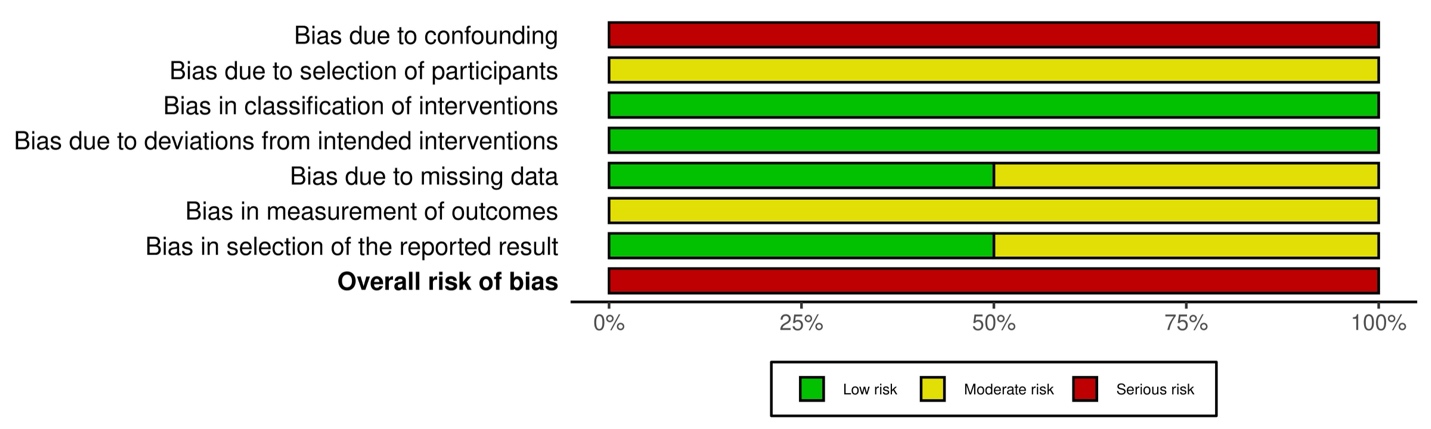


**Figure S42.** Summary plot showing ROBINS-I assessment of the included studies regarding pain reduction with PRF for endodontic procedures.


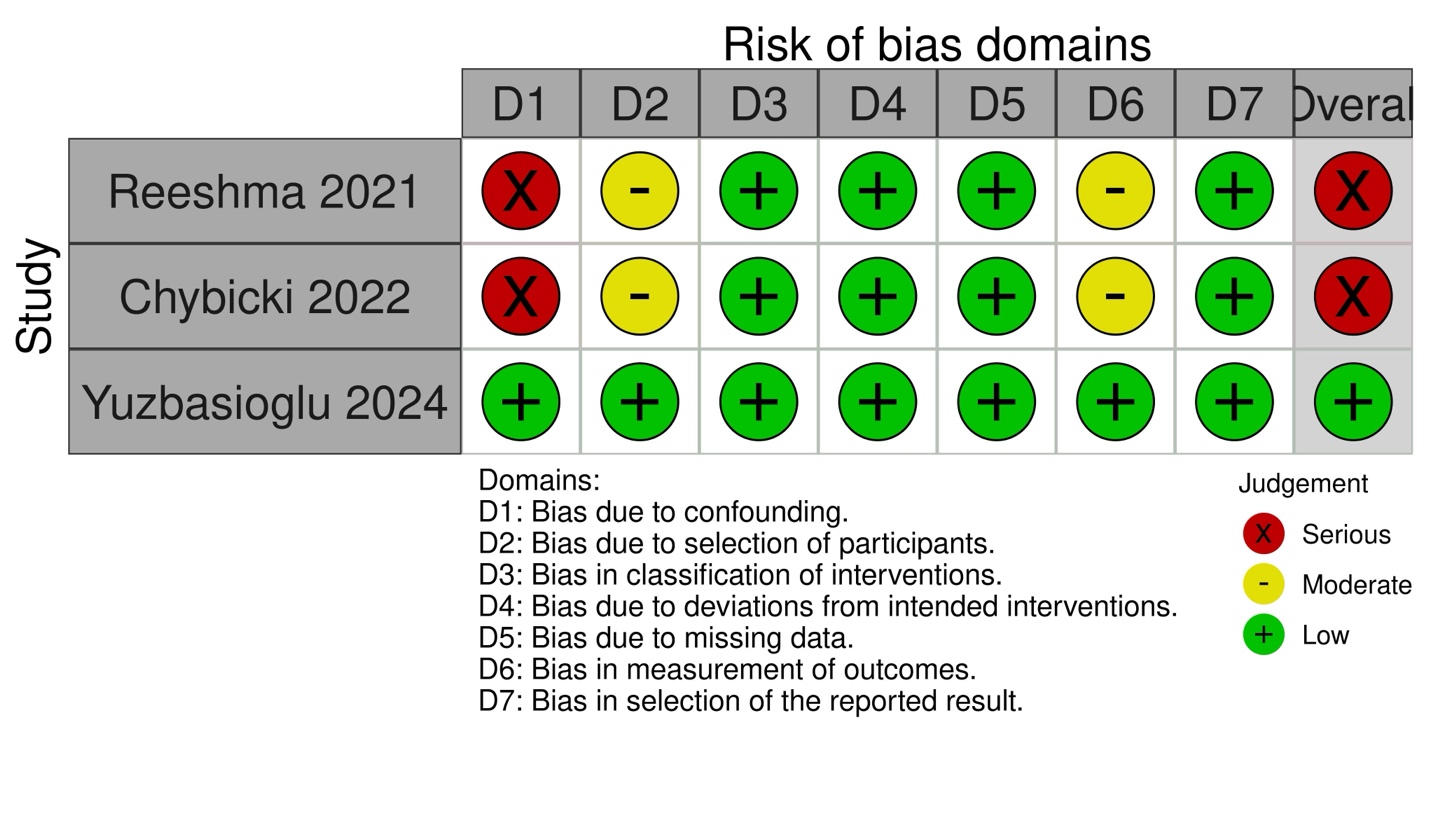


**Figure S43**. Traffic light plot showing ROBINS-I assessment of the included studies regarding pain reduction with PRF for alveolar osteitis (dry socket).


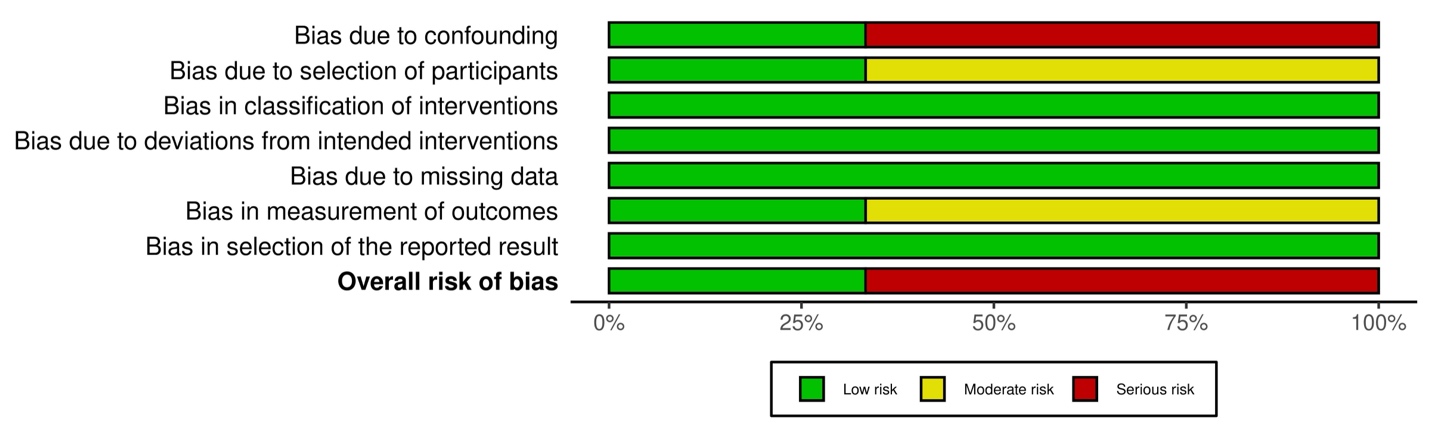


**Figure S44.** Summary plot showing ROBINS-I assessment of the included studies regarding pain reduction with PRF for alveolar osteitis (dry socket).


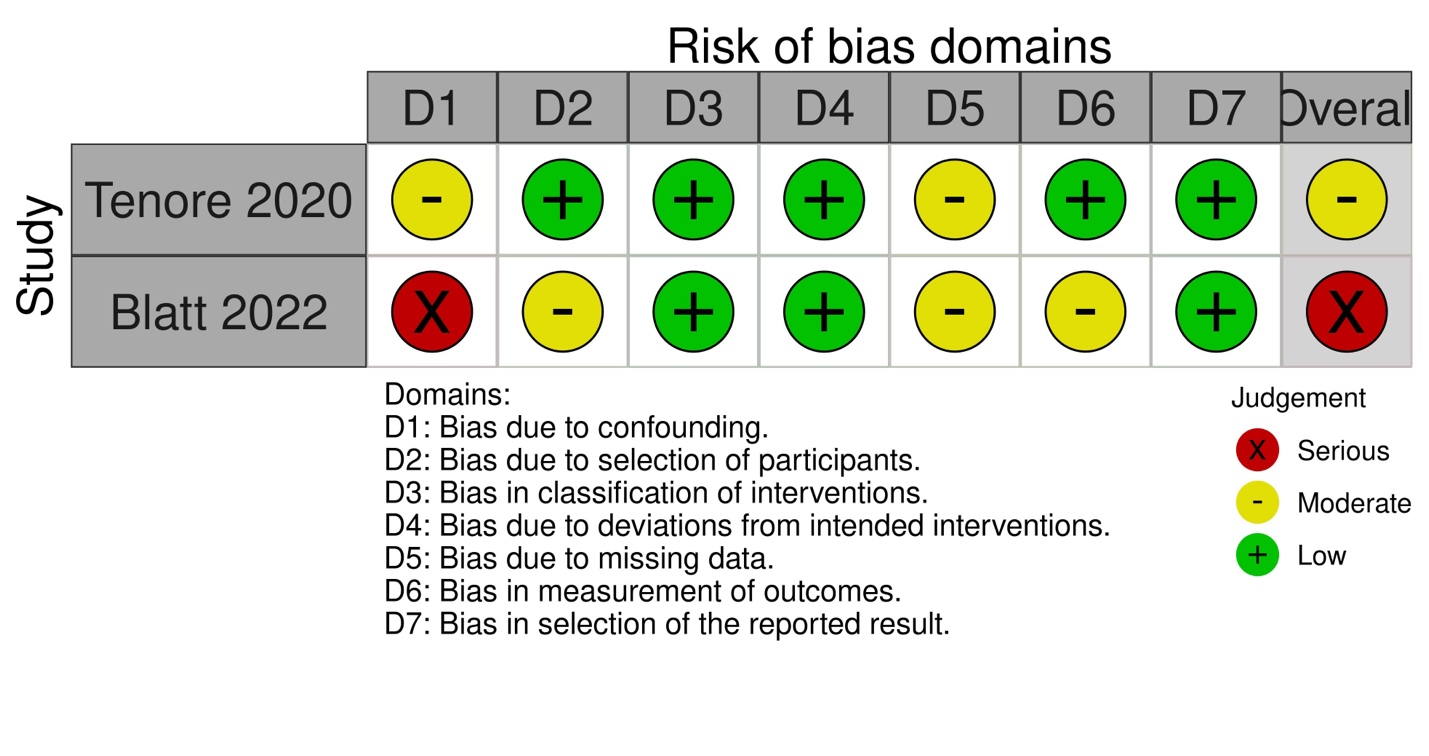


**Figure S45**. Traffic light plot showing ROBINS-I assessment of the included studies regarding pain reduction with PRF for MRONJ.


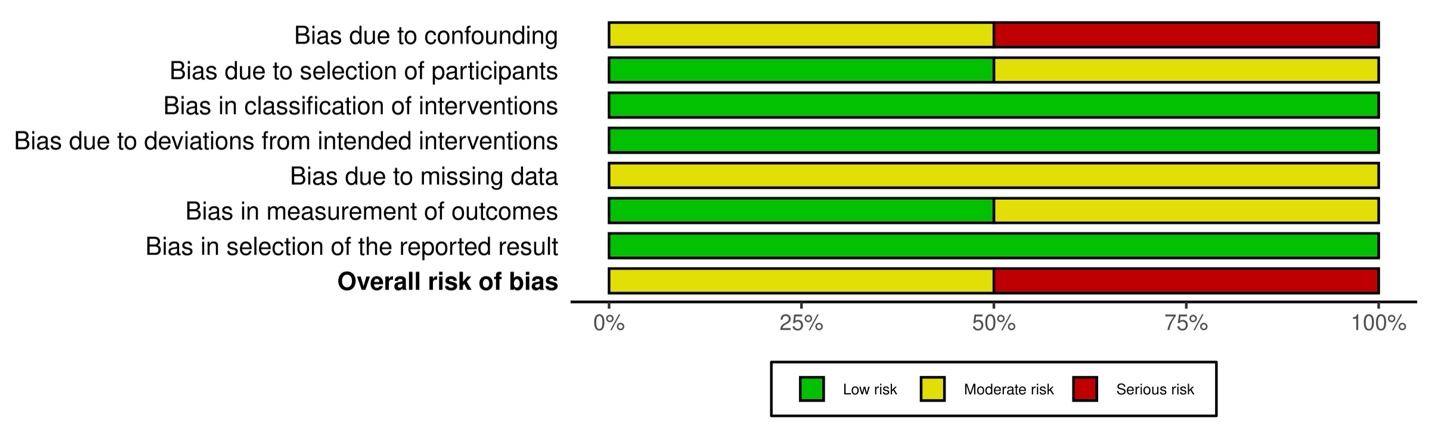


**Figure S46.** Summary plot showing ROBINS-I assessment of the included studies regarding pain reduction with PRF for MRONJ.


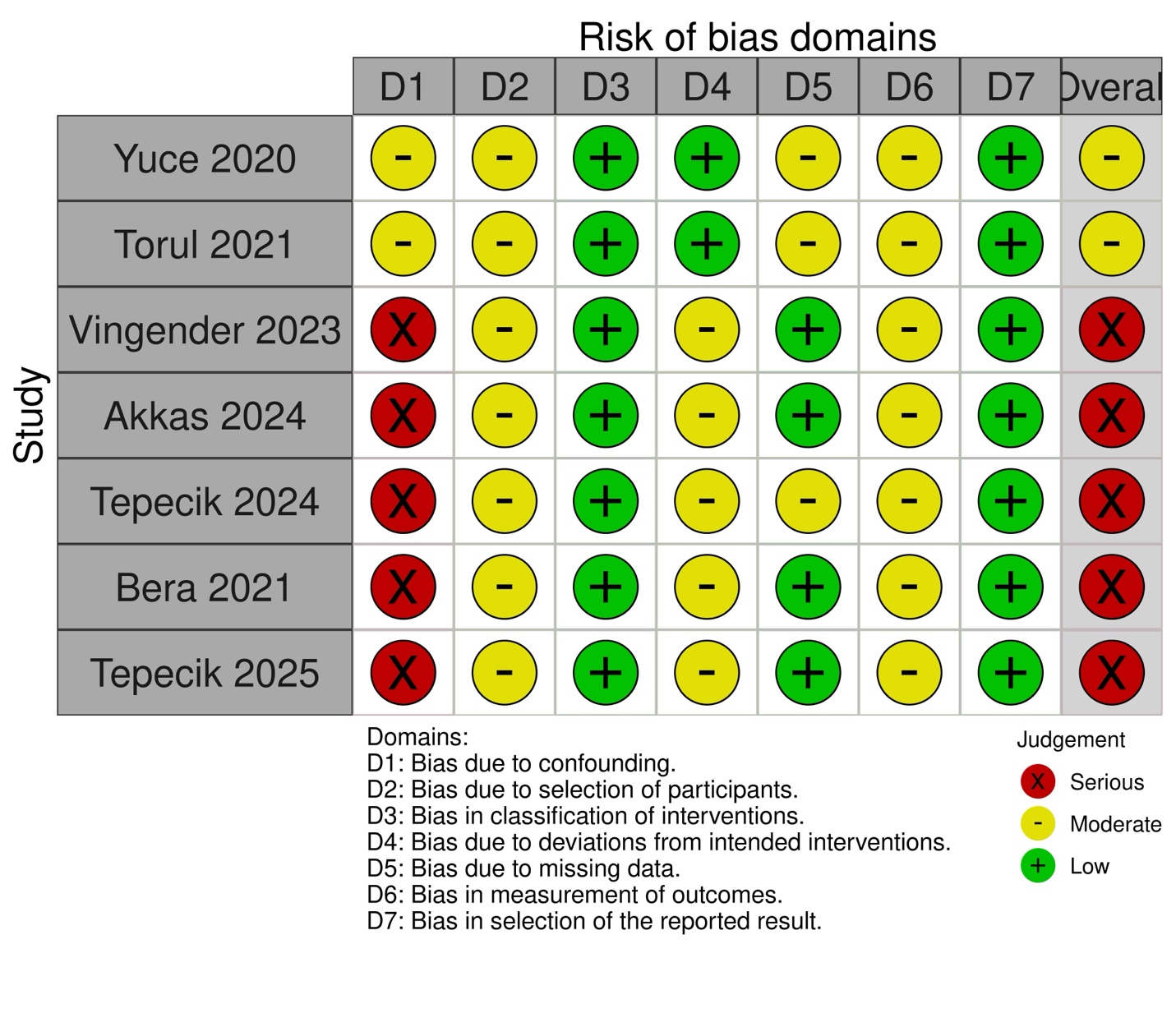


**Figure S47.** Traffic light plot showing ROBINS-I assessment of the included studies regarding pain reduction with PRF for TMJ disorders.


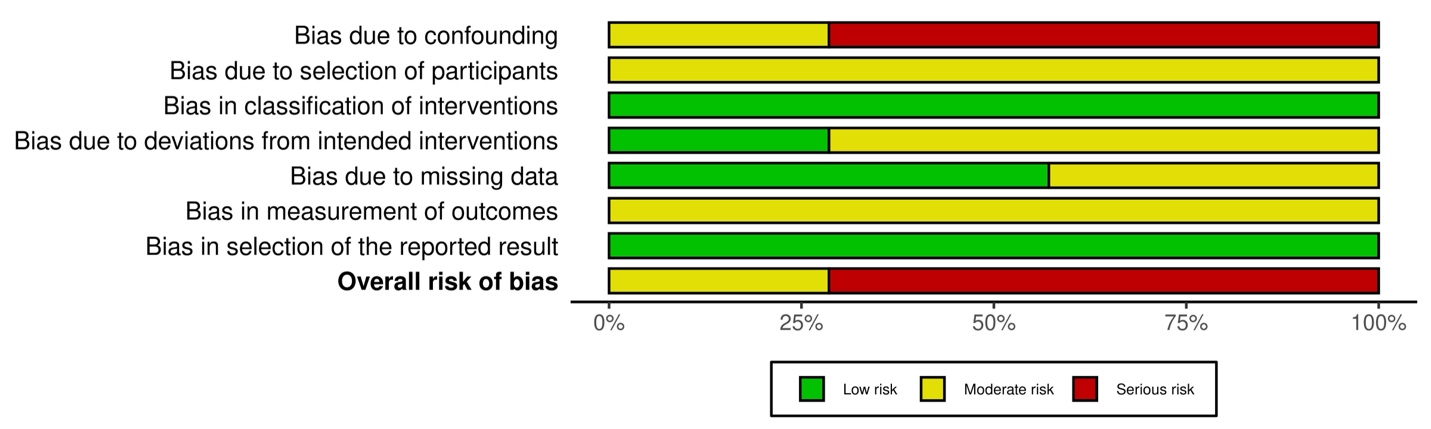


**Figure S48.** Summary plot showing ROBINS-I assessment of the included studies regarding pain reduction with PRF for TMJ disorders.


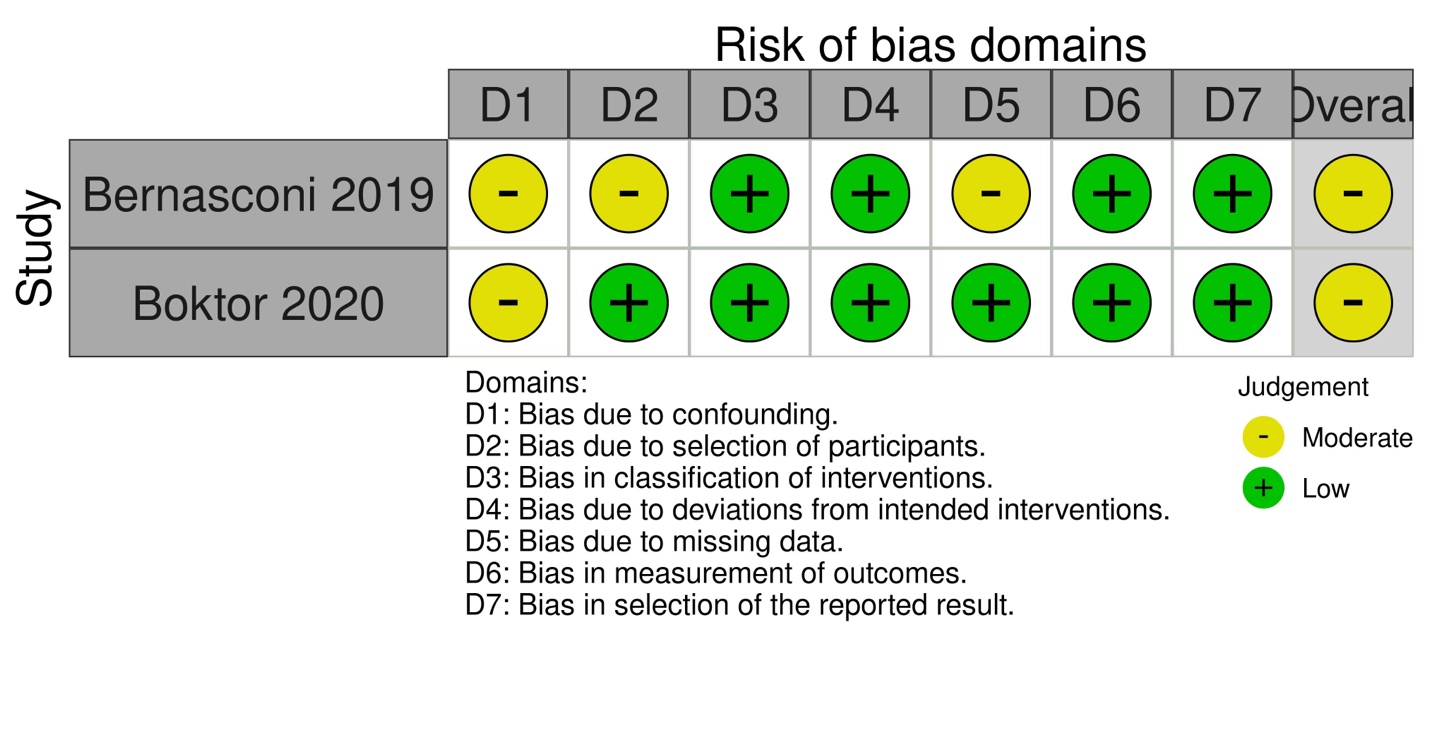


**Figure S49.** Traffic light plot showing ROBINS-I assessment of the included studies regarding pain reduction with PRF for orthopedics.


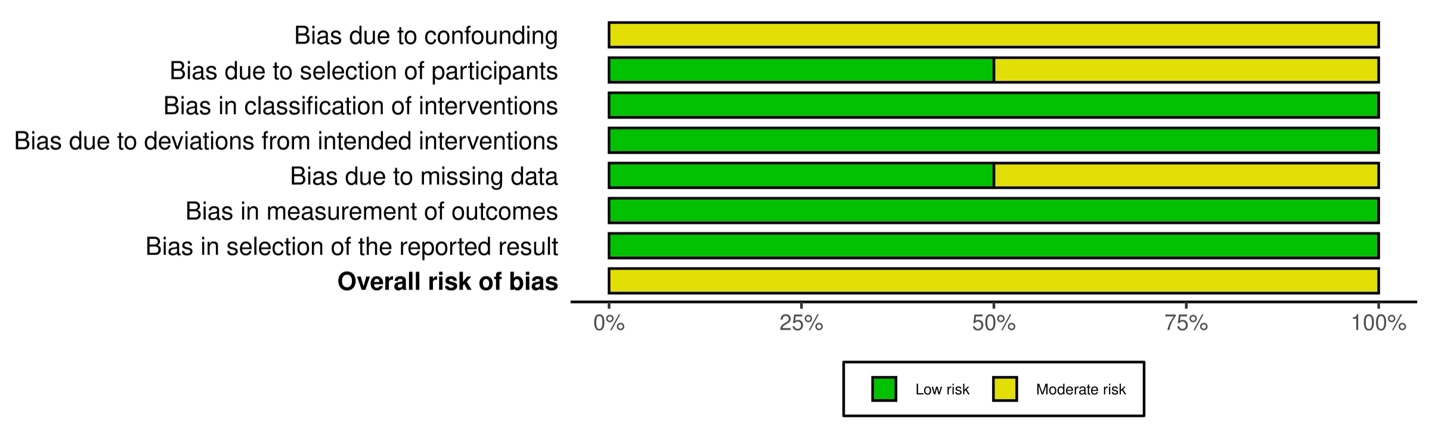


**Figure S50.** Summary plot showing ROBINS-I assessment of the included studies regarding pain reduction with PRF for orthopedics.


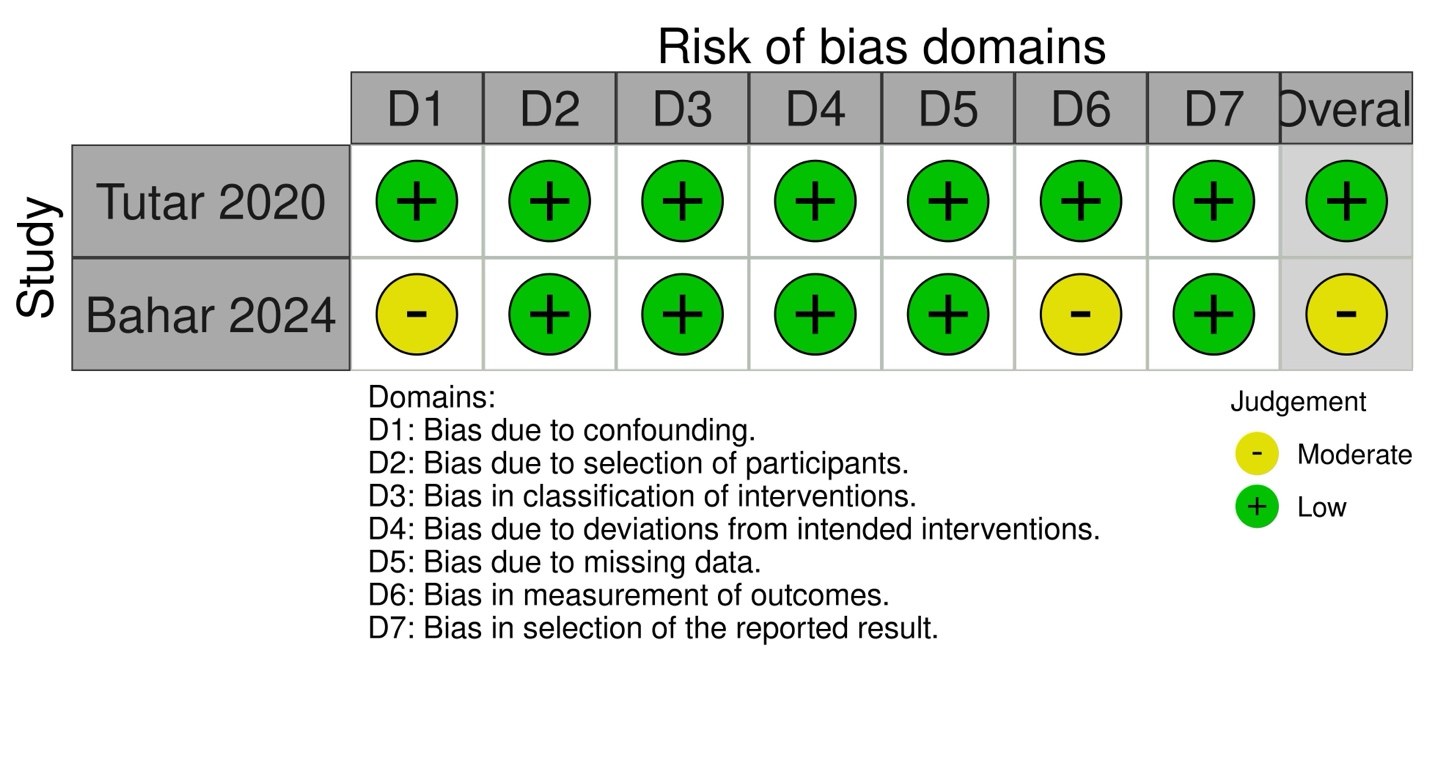


**Figure S51.** Traffic light plot showing ROBINS-I assessment of the included studies regarding pain reduction with PRF for miscellaneous medical procedures.


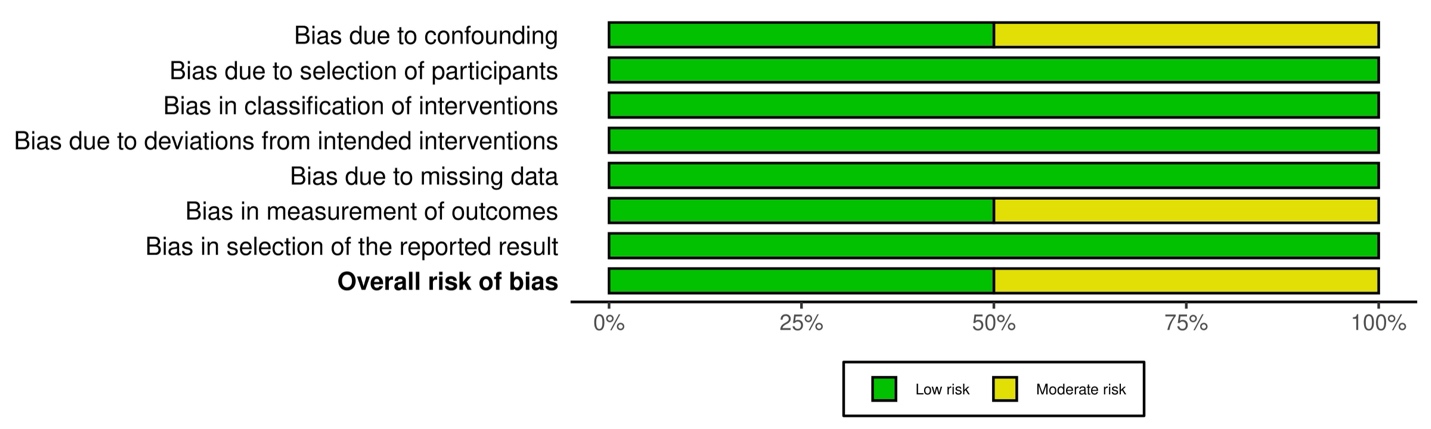


**Figure S52.** Summary plot showing ROBINS-I assessment of the included studies regarding pain reduction with PRF for miscellaneous medical procedures.
